# Bispecific Thio‐Linked Disaccharides as Inhibitors of *Pseudomonas Aeruginosa* Lectins LecA (PA‐IL) and LecB (PA‐IIL): Dual‐Targeting Strategy

**DOI:** 10.1002/chem.202403546

**Published:** 2024-11-28

**Authors:** Lukáš Faltinek, Filip Melicher, Viktor Kelemen, Erika Mező, Anikó Borbás, Michaela Wimmerová

**Affiliations:** ^1^ Department of Biochemistry, Faculty of Science Masaryk University Kotlářská 2 611 37 Brno Czech Republic; ^2^ Central European Institute of Technology Masaryk University Kamenice 5 625 00 Brno Czech Republic; ^3^ National Centre for Biomolecular Research Faculty of Science Masaryk University Kotlářská 2 611 37 Brno Czech Republic; ^4^ HUN-REN-UD Pharmamodul Research Group Egyetem tér 1 4032 Debrecen Hungary; ^5^ Department of Pharmaceutical Chemistry University of Debrecen Egyetem tér 1 4032 Debrecen Hungary

**Keywords:** Lectins, *Pseudomonas aeruginosa*, Synthetic carbohydrates, Inhibition, Agglutination

## Abstract

*Pseudomonas aeruginosa* is a prevalent opportunistic human pathogen, particularly associated with cystic fibrosis. Among its virulence factors are the LecA and LecB lectins. Both lectins play an important role in the adhesion to the host cells and display cytotoxic activity. In this study, we successfully synthesized hardly hydrolysable carbohydrate ligands targeting these pathogenic lectins, including two bispecific glycans. The interactions between LecA/LecB lectins and synthetic glycans were evaluated using hemagglutination (yeast agglutination) inhibition assays, comparing their efficacy with corresponding monosaccharides. Additionally, the binding affinities of bispecific glycans were assessed using isothermal titration calorimetry (ITC). Structural insight into the lectin‐ligand interaction was obtained by determining the crystal structures of LecA/LecB lectins in complex with one of the bispecific ligands using X ray crystallography. This comprehensive investigation into the inhibitory potential of synthetic glycosides against *P. aeruginosa* lectins sheds light on their potential application in antimicrobial therapy.

## Graphical abstract

*Pseudomonas aeruginosa*, a major pathogen linked to cystic fibrosis, relies on the virulence factors, lectins LecA and LecB, responsible for the cytotoxicity and adhesion to host cells. As inhibitors of these lectins, thiodisaccharides were synthesized including bispecific compounds targeting both lectins. Their inhibitory potential was confirmed, and structural insights were gained from ligand‐lectin interactions.

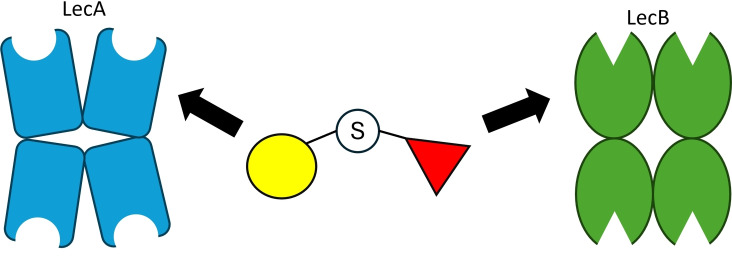

## Introduction

*Pseudomonas aeruginosa* is an opportunistic pathogen notorious for both acute and chronic infections, particularly among cystic fibrosis patients.[^1^, ^2^] Since limited therapeutic options exist due to escalating antibiotic resistance of *P. aeruginosa*,[^3^, ^4^, ^5^] alternative therapeutic strategies such as anti‐adhesion therapy are imperative.[^6^, ^7^] Adherence to host tissues is crucial for *P. aeruginosa* infection initiation, often facilitated by host cell surface glycoconjugates, making them prime targets for bacterial receptors. Oligosaccharide‐mediated bacterium‐cell recognition and adhesion are crucial in early pathogenesis.[^8^, ^9^] In this process, *P. aeruginosa* employs various molecules, including lectins,[^8^, ^10^, ^11^] which are proteins generally involved in numerous physiological and pathophysiological mechanisms.[^12^, ^13^] Lectins are characteristic of lacking catalytic activity; however, they exhibit a remarkable ability to reversibly bind mono‐ and oligosaccharides with exceptional specificity. They are abundantly expressed in viruses, bacteria, and fungi.[^12^, ^13^, ^14^, ^15^]

In *P. aeruginosa*, two soluble lectins, LecA and LecB were initially identified and characterized.^[16]^ Both lectins are calcium‐dependent tetrameric proteins with one binding site per monomer. On the monosaccharide level, LecA is specific towards d‐galactose (Gal), while LecB binds l‐fucose (Fuc) with the highest affinity but can also recognize other monosaccharides such as d‐mannose.[^16^, ^17^, ^18^] Both lectins are considered to be significant virulence factors.^[8]^ Besides their proposed primary function as adhesins, lectins also participate in biofilm formation and exhibit cytotoxic effects.[^19^, ^20^, ^21^, ^22^] Specifically, LecA decreases the growth rate of respiratory epithelial cells,^[23]^ while LecB blocks epithelial cells’ ciliary beating.[^8^, ^24^] Due to their importance in the pathogenesis of *P. aeruginosa*, the LecA and LecB lectins could represent a suitable therapeutic target. For this purpose, several types of carbohydrate inhibitors were designed and tested against these lectins,^[25]^ including multivalent inhibitors such as fullerene‐based glycoclusters,^[26]^ glyco(peptide)dendrimers,[^27^, ^28^, ^29^] resorcin[4]arene‐based,^[30]^ and calix[4]arene‐based glycoclusters.[^31^, ^32^] In addition, polymeric nanoparticles^[33]^ or mono‐/divalent aryl‐glycoconjugates[^19^, ^34^] were prepared. Recently, strategies involving the development of non‐carbohydrate glycomimetics or covalent lectin inhibitors, which target a subpocket located between two carbohydrate‐binding sites, were introduced.[^35^, ^36^]

Native carbohydrates as well as most synthetic ligands documented to date involve linking sugar units via an *O*‐glycosidic bond.[^37^, ^38^, ^39^] *O*‐glycosidic linkages are not ideal for bioassays and therapeutic applications because they are sensitive to degradation by glycosidases.[^40^, ^41^] Therefore, enzymatically stable inhibitors could be utilized as suitable ligands for *P. aeruginosa* lectins, such as those with S‐ and Se inter glycosidic linkages.[^42^, ^43^, ^44^, ^45^]

In this study we focus on non‐reducing homo‐ and heterodisaccharides which can serve as mono‐ or bivalent inhibitors. We designed l‐fucose‐containing ligands selective towards LecB, d‐galactose‐containing ligands with selectivity to LecA, and also envisioned bifunctional/bispecific Gal‐Fuc disaccharides that could potentially bind to both lectins. One of our goals was the systematic investigation of how the stereochemistry of the glycosidic bonds affects the binding affinity to the lectin. The lack of study of the anomeric stereochemistry of lectin ligands is due to the fact that the synthetic availability of the α‐ and β‐glycosidic bonds is very different; while the formation of the 1,2‐trans‐β‐glycosides can be easily obtained in a stereoselective manner by many methods, the synthesis of the 1,2‐cis‐α bonds is very challenging and has not been efficiently achieved by conventional methods.^[46]^ We have shown that the photoinitiated addition of thiols to 2‐substituted pyranosyl glycals affords 1,2‐cis‐α‐thioglycosides efficiently and with complete stereoselectivity, regardless of the sugar configuration.[^47^, ^48^] Here, this thiol‐ene coupling, also known as the thio‐click method, was exploited to prepare α,α‐ and α,β‐linked thiodisaccharides to study their interaction with LecA and LecB lectins.

The interactions between lectins and synthetic compounds were investigated by the agglutination inhibition assay. The most promising compounds, particularly the bispecific ones, underwent further analysis using isothermal titration calorimetry. Additionally, the crystal structures of LecA/LecB lectins complexed with one of the bispecific ligands were determined using X‐ray crystallography to gain a structural understanding of the lectin‐ligand interaction. Our findings revealed that mono‐ and bivalent thiogalactosides and thiofucosides demonstrated potential as inhibitors for biomedically relevant lectins LecA/LecB. Notably, the multifunctional property of bispecific inhibitors is promising as they can bind to both fucose‐ and galactose‐specific lectins. Overall, our investigation into the inhibitory properties of synthetic glycosides against pathogenic lectins holds great promise for their potential application in antimicrobial therapy.

## Results and Discussion

**Figure 1:**
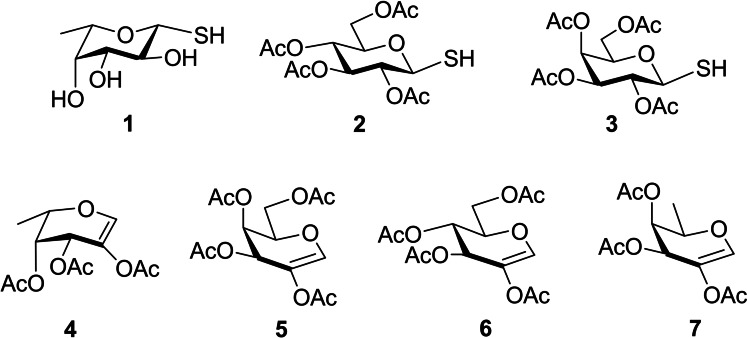
β‐1‐Thiosugars **1**–**3** and glycals **4**–**7** used for the synthesis of non‐reducing α,β‐ and α,α‐thiodisaccharides.

The photochemical thiol‐ene addition reaction is a well‐known thioconjugation process based on the regioselective reaction of an electrophilic thiyl radical and an electron‐rich double bond.^[49]^ We recently demonstrated that, using readily available β‐1‐thiosugars as thiol partners and 2‐substituted hexoglycals as alkene partners, this method provides rapid and fully stereoselective access to α,β‐linked thiodisaccharides.[^50^, ^51^] Accordingly, β‐1‐thiosugars **1**–**3** and 2‐acetoxy‐l‐fucal **4** and 2‐acetoxy‐d‐galactal **5** were used to obtain α,β‐thiodisaccharides containing l‐fucose and d‐galactose. The planned α,α‐thiodisaccharides were prepared from glycals **4**–**7** via two consecutive thiol‐ene coupling reactions (vide infra) (Figure 1).

**Scheme 1:**
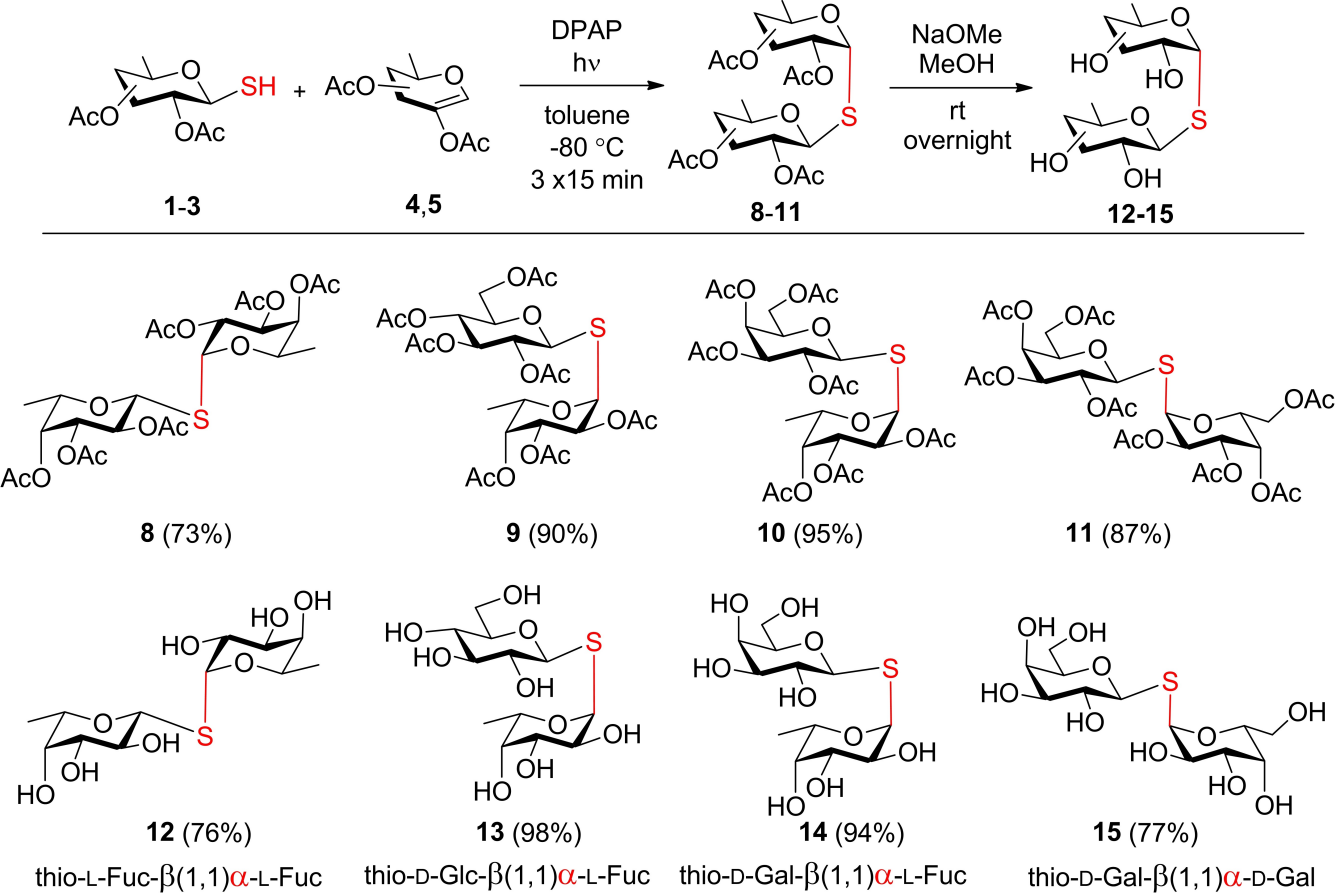
Synthesis of the α,β‐linked thiodisaccharides **12**–**15** by photoinitiated thiol‐ene coupling followed by deacetylation (the α‐glycosidic linkages formed in the thiol‐ene reactions are highlighted in red).

The addition of β‐glycosyl thiols **1**–**3** onto glycals **4** and **5** were carried out upon three UVA‐light (λ_max_ 365 nm) irradiation cycles in the presence of the photoinitiator 2,2‐dimethoxy‐2‐phenylacetophenone (DPAP) at −80 °C; the low temperature increases the stability of the intermediate carbon‐centered radical formed in the first, reversible thiyl addition step of the reaction, thereby preventing its entropy‐favored decomposition and promoting efficient addition.[^50^, ^51^] The reactions proceeded with full 1,2‐cis‐α‐selectivity providing the desired protected α,β‐thiodisaccharides **8**–**11** in high yields (Scheme 1). By removing the acetyl protecting groups under Zemplén conditions, free α,β‐thiodisaccharides **12**–**15**, of which **12** is a bivalent ligand, **13** is a monovalent ligand for LecA, and **15** is a bivalent ligand for LecB, while compound **14** can be considered a bifunctional ligand with affinity for both lectins.

**Scheme 2:**
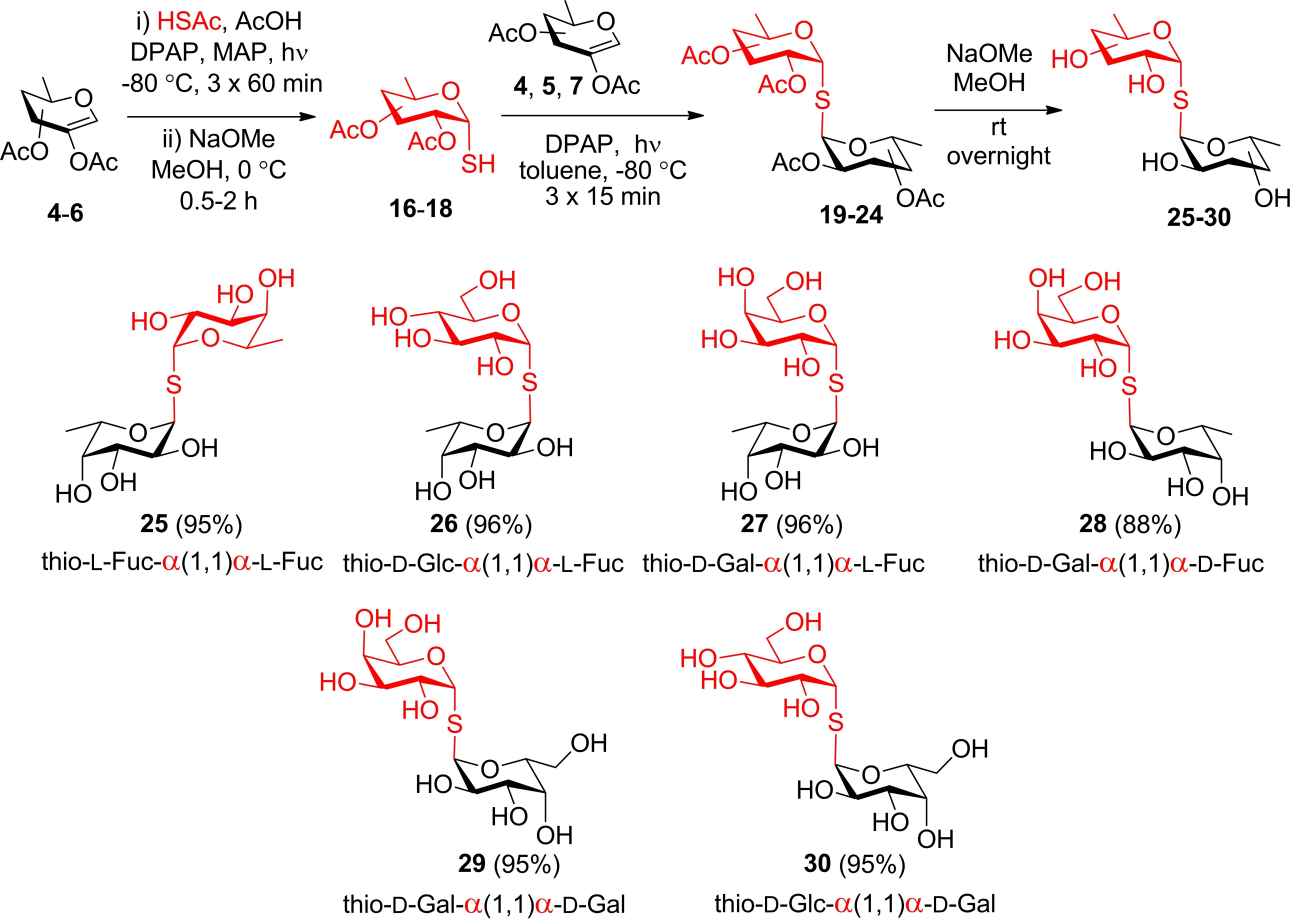
Synthesis of the α,α‐linked thiodisaccharides **25**–**30** by two sequential thiol‐ene couplings followed by deacetylation.

For the synthesis of α,α‐thiodisaccharides the corresponding α‐1‐thiosugars were prepared by the thiol‐ene coupling reaction of glycals and thioacetic acid (HSAc), using recently optimized conditions.^[52]^ Due to the low reactivity of HSAc in the radical reaction, we used a longer reaction time and 4‐methoxyacetophenone (MAP) as a co‐initiator. Under these conditions, the addition gave the desired α‐S‐acetyl derivatives quite efficiently in toluene, but when the solvent was changed to acetic acid, a further 10–20 % increase in yield was achieved (Scheme 2). After selective S‐deacetylation, the obtained α‐configured l‐fucosyl, d‐glucosyl and d‐galactosyl thiols **16**–**18** were subjected to a second thiol‐ene coupling reaction with glycals **4**, **5** and **7** to obtain the protected α,α‐thiodisaccharides **19**–**24**. The final Zemplén deacetylation step provided the free α,α‐thiodisaccharides **25**–**30** with l‐fucose, 6‐deoxy‐d‐galactose (d‐fucose) and d‐galactose content.

**Figure 2:**
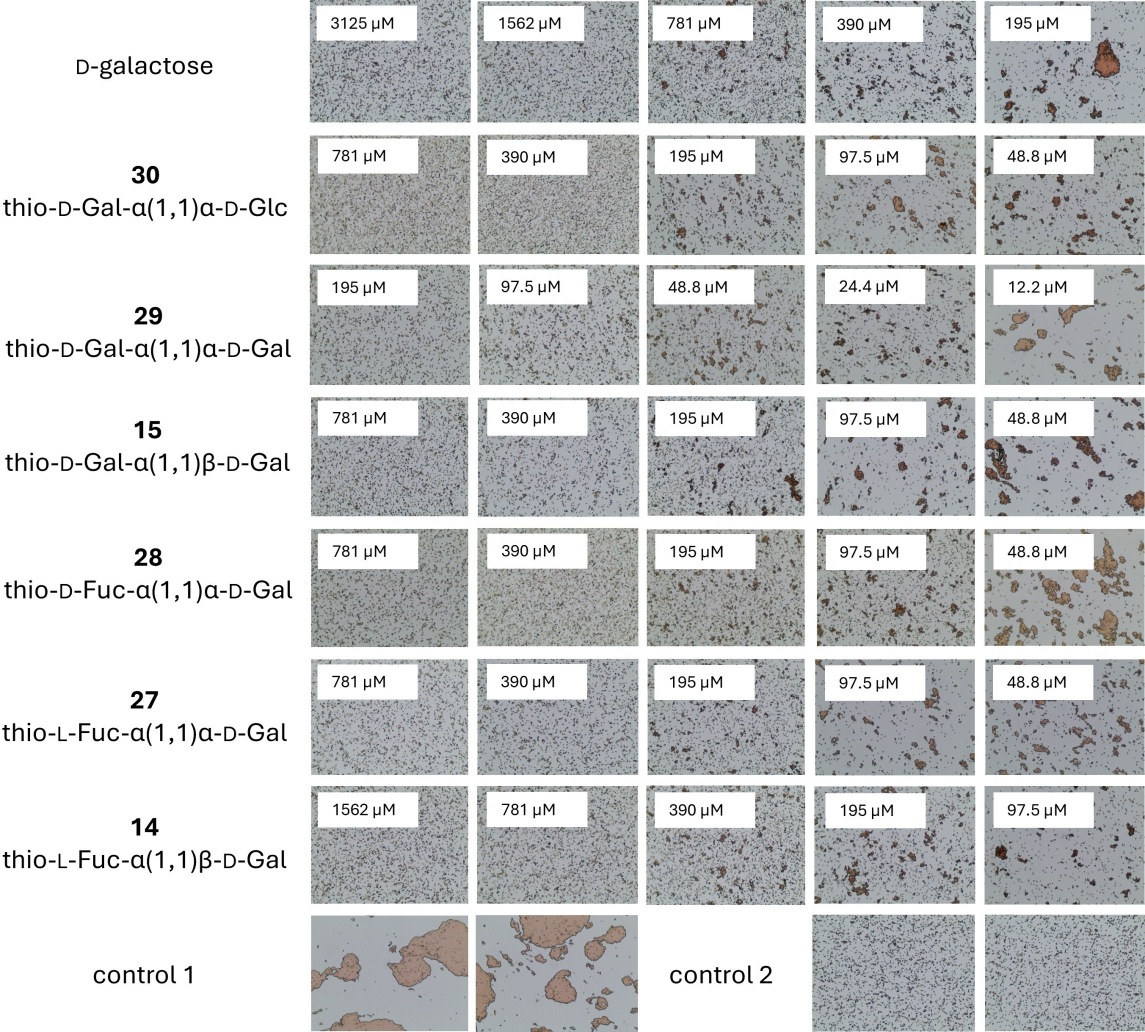
Influence of d‐galactose and disaccharide inhibitors on hemagglutination caused by lectin LecA. Control 1: experiment without inhibitor. Control 2: experiment without lectin LecA.

**Figure 3:**
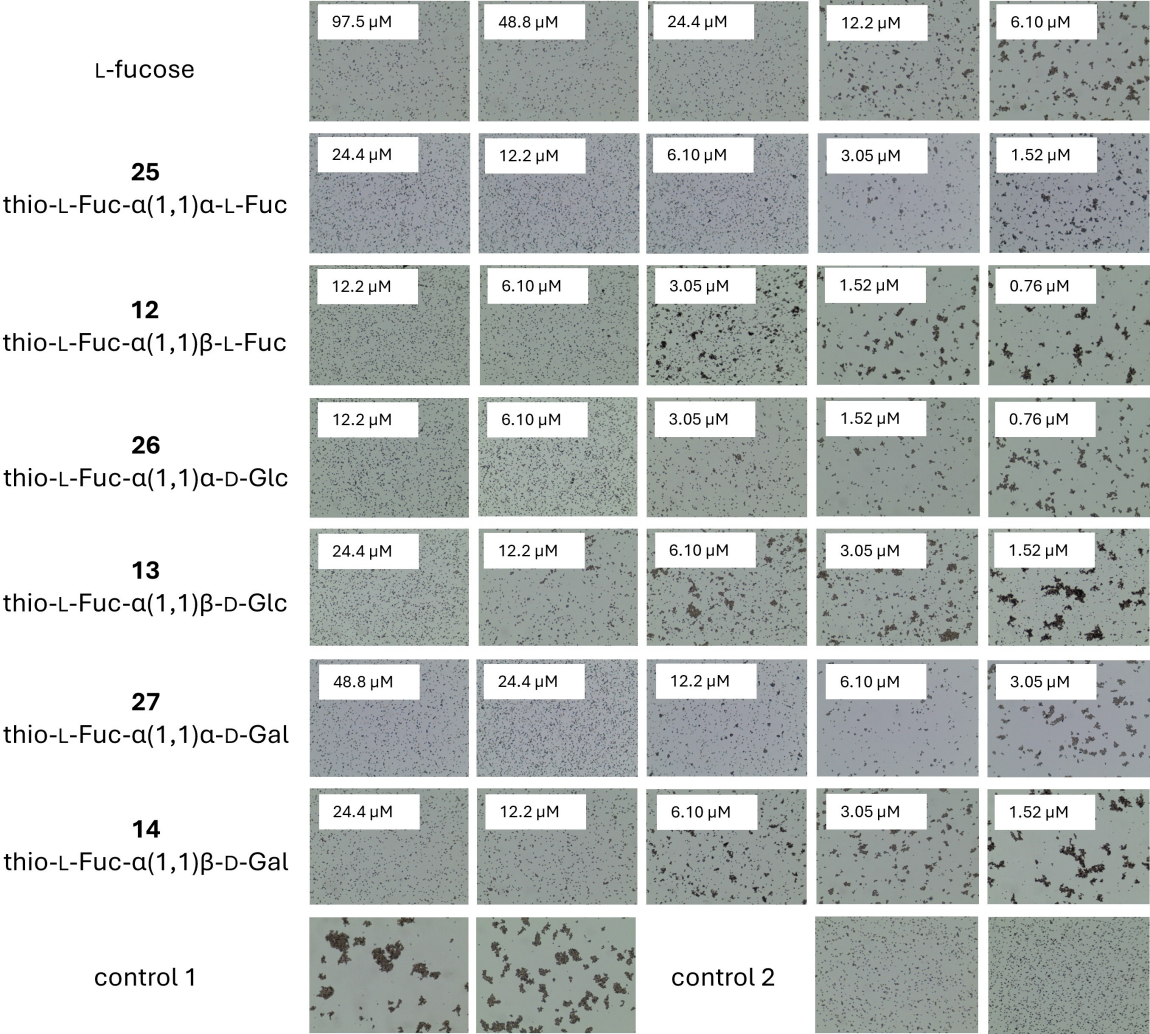
Influence of l‐fucose and disaccharide inhibitors on yeast agglutination caused by lectin LecB. Control 1: experiment without inhibitor. Control 2: experiment without lectin LecB.

Using agglutination inhibition assays, we evaluated the interactions between the synthetic compounds and the lectins. For LecA, the preliminary inhibition assay employing red blood cells (RBCs) of group O in a microtiter plate format was done to see possible inhibition of hemagglutination by the naked eye. Subsequently, microscopy was utilized as the detection method (Figure 2) as described previously.^[53]^ Microscopy format was used also for LecB (Figure 3) where yeast cells were employed since the lectin displays the ability to bind also d‐mannose. The final concentration was 125 μg/ml and 625 μg/ml for LecA and LecB, respectively.

The minimal inhibitory concentrations (MICs) of carbohydrates able to completely inhibit agglutination caused by the lectins were determined and the potencies of the inhibitors were calculated (Table 1) by comparison with the standard (Gal and Fuc for LecA and LecB, respectively). Investigations revealed inhibitory effects for all tested compounds, as evidenced by their ability to inhibit lectin‐induced agglutination. The calculated potency varied from 1 to 4 in comparison to standard monosaccharides. However, compound **29** (α‐d‐Gal‐α‐d‐Gal) was revealed to be one order of magnitude better inhibitor than d‐galactose for LecA. This proves that the divalent presentation of specific sugar units is indeed sufficient to achieve the multivalent effect. Although other divalent glycomimetics have shown higher affinities,^[54]^ this can be largely attributed to differences in the experimental setup, such as the use of surface plasmon resonance since in previous studies, notable differences between methods for the same inhibitor have been observed.^[32]^

**Table 1:** Minimal inhibitory concentrations (MICs) of tested carbohydrates and potencies of tested inhibitors determined by an agglutination inhibition assay.

Carbohydrate ompound		LecA		LecB	
		MIC [μM]	potency	MIC [μM]	potency
–	d‐Gal	1562	1	–	–
–	l‐Fuc	–	–	24.4	1
**25**	Thio‐l‐Fuc‐α(1,1)α‐l‐Fuc	–	–	6.10	4
**12**	Thio‐l‐Fuc‐α(1,1)β‐l‐Fuc	–	–	6.10	4
**26**	Thio‐l‐Fuc‐α(1,1)α‐d‐Glc	–	–	6.10	4
**13**	Thio‐l‐Fuc‐α(1,1)β‐d‐Glc	–	–	24.4	1
**27**	Thio‐l‐Fuc‐α(1,1)α‐d‐Gal	390	4	24.4	1
**14**	Thio‐l‐Fuc‐α(1,1)β‐d‐Gal	781	2	12.2	2
**28**	Thio‐d‐Fuc‐α(1,1)α‐d‐Gal	390	4	–	–
**30**	Thio‐d‐Gal‐α(1,1)α‐d‐Glc	390	4	–	–
**29**	Thio‐d‐Gal‐α(1,1)α‐d‐Gal	97.5	16	–	–
**15**	thio‐d‐Gal‐α(1,1)β‐d‐Gal	390	4	–	–

Furthermore, the results highlight the importance of the configuration of the glycosidic bond on the potency. According to our results, LecA is particularly sensitive to anomeric configuration and highly prefers the α‐interglycosidic bond, as evidenced by the four times higher affinity for the α,α‐di‐Gal ligand **29** compared to the α,β‐di‐Gal ligand **15**. On the other hand, we were unable to investigate any potential preference between α/β‐fucosides for LecB due to the lack of suitable compounds containing β‐interglycosidic bond. Interestingly, the difference in affinity for α/β‐glucosides was observed, although in both cases the compound contained α‐fucose (see the affinity of **13** vs **26**).

Notably, multivalent inhibitors, such as glycoclusters, often exhibit higher potency towards *P. aeruginosa* lectins than monovalent analogues, especially due to their ability to simultaneously engage multiple binding sites on the lectins, leading to enhanced inhibitory effects.[^27^, ^32^, ^44^, ^55^, ^56^] Nevertheless, due to the high molecular weight and complex structure, glycodendrimers and glycoclusters have poor pharmacokinetic properties, which hinders their therapeutic applicability. Therefore, more and more attention has recently been directed towards small‐molecule mono‐ and bivalent inhibitors, which are much more drug‐like molecules.[^57^, ^58^, ^59^] Also, our synthesized glycosides offer advantages in term of excellent solubility in aqueous solutions and higher stability than their natural saccharide counterparts due to the lower reaction rates in both acid and enzyme catalyzed hydrolysis.[^40^, ^60^, ^61^]

Among the tested inhibitors, bispecific carbohydrates designed as the compound **27** (α‐Fuc‐α‐Gal) and **14** (α‐Fuc‐β‐Gal) were assessed against both lectins. It was proven that the bispecific compounds inhibited agglutination in the case of both LecA and LecB lectins, underscoring their potential as versatile therapeutic agents in antimicrobial therapy.

**Figure 4:**
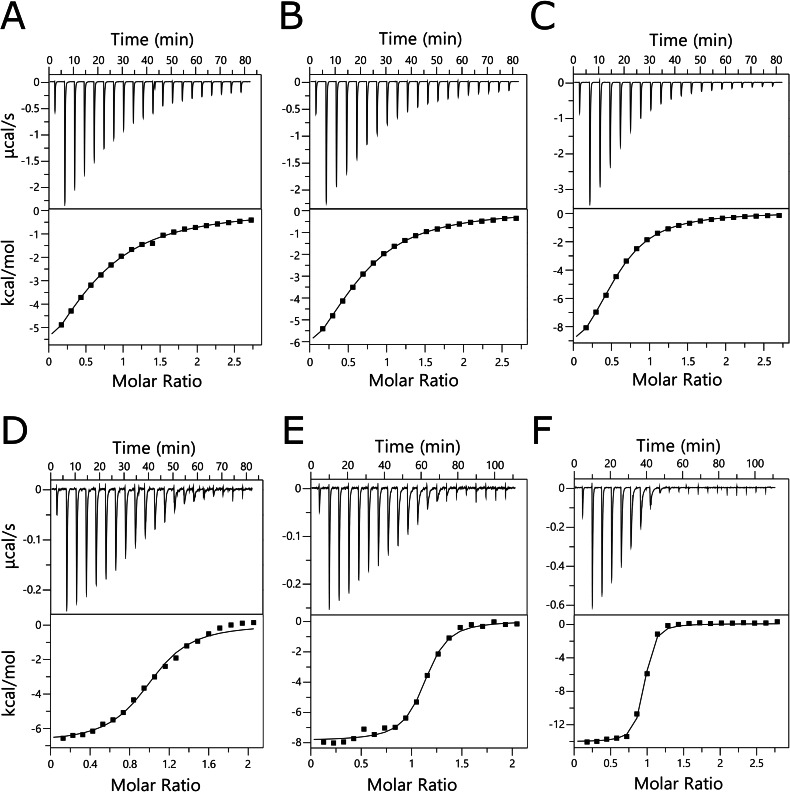
Isothermal titration calorimetry of LecA with d‐galactose (A), compound **14** (B), and compound **27** (C), and LecB with l‐fucose (D), compound **14** (E), and compound **27** (F). For each experiment, the concentration of the lectin was 300 μM and 40 μM, and the concentration of each carbohydrate was 2 mM and 0.4 mM for LecA and LecB, respectively. Bottom plots show the total heat released as a function of total ligand concentration for the titration shown in the upper plots.

Subsequently, the two bispecific compounds underwent further analysis through isothermal titration calorimetry. The experiments in solution confirmed the interaction between bispecific inhibitors and both lectins (Figure 4), yielding stoichiometry and the equilibrium dissociation constants (K_D_), as is shown in Table 2. As control ligands, Gal and Fuc were tested for LecA and LecB, respectively. In the case of LecA, K_D_ of all ligands was in the submillimolar range, whereas, for LecB, K_D_ of bispecific inhibitors reached the submicromolar range. For both lectins, compound **27** (α‐l‐Fuc‐α‐d‐Gal) was discovered as the best binding partner followed by compound **14** (α‐l‐Fuc‐β‐d‐Gal). Compound **27** possessed approximately three times and ten times higher affinity than the corresponding monosaccharide for LecA and LecB, respectively. The observation of significantly increased affinity, despite the monovalency of bispecific inhibitors, is supported by their potency calculation based on agglutination inhibition experiments. Efficiency in inhibiting agglutination is higher in most tested monovalent disaccharide compounds. It's plausible that the interaction between the inhibitor and the lectin is entropically more favorable than in the free monosaccharide case, as Navarra discussed in his work.^[62]^

**Table 2:** Stoichiometry and the calculated K_D_ values (in μM) for the interaction between LecA/LecB and individual ligands determined by isothermal titration calorimetry (ITC).

	LecA		LecB	
Carbohydrate	n*	KD [μM]	n	KD [μM]
d‐Galactose	0.60	175	–	–
l‐Fucose	–	–	0.98	1.9
compound **14**	0.62	115	1.00	0.35
Compound **27**	0.53	55.6	0.90	0.19

*Apparent value due to low affinity.

Since experiments with LecA did not result in the sigmoid shape of ITC curves,^[63]^ stoichiometry is burdened with a larger deviation since its value should theoretically be close to 1. However, the experiments clearly showed the interaction and thus confirmed the agglutination inhibition experiments showing that the bispecific ligands are able to bind both lectins.

Further, we aimed to investigate whether the bispecific inhibitors are capable of binding both lectins simultaneously, as this effect could lead to precipitation, which may not be desirable for anti‐adhesive therapeutics in a biological environment. In contrast to the potential cross‐linking observed with some multivalent inhibitors, which were tested and demonstrated the ability to cross‐link at the cellular level,^[55]^ our goal is to avoid this phenomenon. To this end, we utilized the dynamic light scattering (DLS) method, which did not reveal the formation of larger aggregates in the presence of a mixture of the lectins and compound **14** at any concentration used. Nonetheless, DLS provided results with limited sensitivity (Figure S1) that may not have revealed the presence of other oligomeric states. Therefore, we conducted an analytical ultracentrifugation (AUC) analysis (Figure S2). The AUC experiments, using mixtures of both lectins with various concentrations of compound **14**, showed no difference in the oligomeric state compared to the lectins without the inhibitor, thereby corroborating the results obtained from DLS.

**Figure 5:**
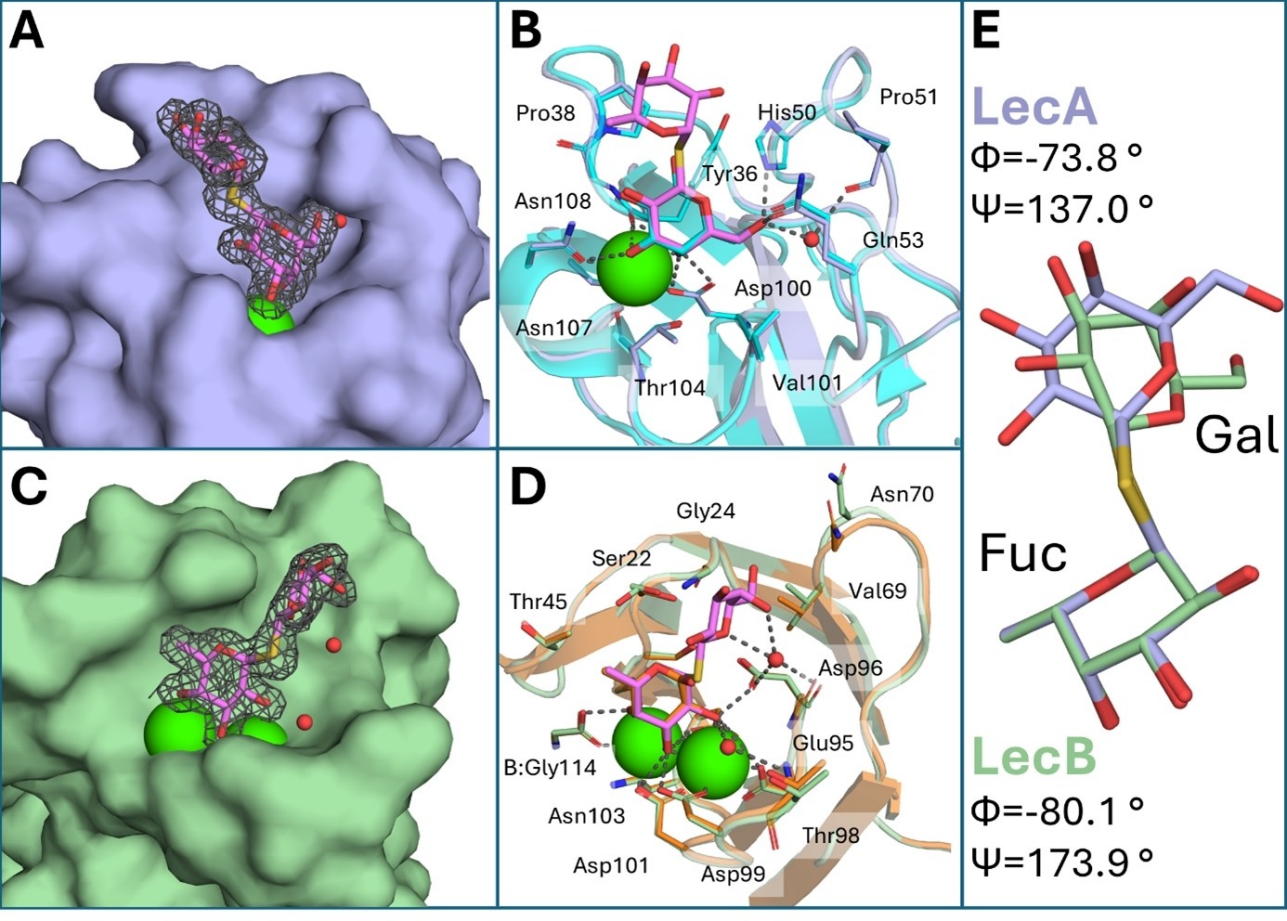
(A) Structure of LecA in complex with compound **14**; (B) Structural alignment of LecA‐**14** (PDB:9G3R, purple) with LecA‐d‐galactose (PDB:1OKO, cyan); (C) Structure of LecB in complex with compound **14**; (D) Structural alignment of LecB‐**14** (PDB:9G3S, pale green) with LecB‐l‐fucose (PDB:1GZT, bright orange). Electron density (blue mesh) is displayed at 1σ, compound **14** and amino acids residues involved in the interaction as sticks, Ca^2+^ ions are green, and water molecules are red spheres. Hydrogen contacts are represented as grey dashed lines. (E) Alignment of the compounds **14** structures via l‐fucose bound with LecA (purple) and LecB (pale green). Torsion angles are defined as Φ: FucO5‐FucC1‐S‐GalC1 and Ψ: FucC1‐S‐GalC1‐GalC2.

Additionally, we successfully co‐crystallized the LecA‐**14** and LecB‐**14** complexes to characterize the structural interactions between bispecific α,β‐thiodisaccharides and lectins. Statistics from the data collection and structure refinement are listed in Table S1. The LecA‐**14** complex exhibited diffraction to 1.78 Å and crystallized in the *P*12_1_1 space group, with 8 protomers in the asymmetric unit (ASU). Electron density mapping at a 3σ level enabled the placement of β‐d‐galactose in all eight monomers. At the same time, the α‐l‐fucose moiety of compound **14** was predominantly solvent‐exposed, observable only in chains A, B, D, G, and H (Figure 5A). Structural alignment of the LecA‐**14** binding site with the previously characterized LecA‐d‐galactose (**PDB : 1OKO**) revealed the preserved position of all amino acids involved in saccharide stabilization. Examination of the binding site affirmed that the substitution of O1 with sulfur S1 in compound **14** did not alter the position of the galactose molecule; the classical orientation of the moiety was maintained, along with calcium coordination of O3 and O4, and polar contacts similar to previously described thio‐galactosides.[^18^, ^19^, ^59^] LecA did not interact with the α‐l‐fucose moiety of compound **14** due to its high solvent exposure (Figure 5B).

The structure of the LecB‐**14** complex was resolved at 1.85 Å in the *P*2_1_2_1_2_1_ space group, with 8 protomers in the ASU. Examination of the Fo−Fc electron density map facilitated the unambiguous placement of 8 copies of compound **14** in the model, one for each of the 8 monomers in the ASU (Figure 5C). Structural comparison of the LecB‐l‐fucose (**PDB : 1GZT**) and LecB‐**14** binding sites revealed no change in the position and orientation of the thiofucoside in the binding pocket. The α‐l‐fucose moiety interacted with calcium ions via O2, O3, and O4, while the C6 methyl group was stabilized with Thr45 via hydrophobic contact.^[64]^ The β‐d‐galactose is situated within a binding groove, anchored by a water bridge connecting its O4 and O5 oxygens to Asp96, and engaged in interaction with the C6 carbon and a hydrophobic patch formed by Val69 and Gly24 (Figure 5D). The structural alignment of compound **14** structures bound to LecA and LecB, respectively, via l‐fucose, unveiled a tilt of the d‐galactose induced by the interaction between compound **14** and the binding groove of LecB (Figure 5E).

Notably, the structural models derived from the lectin‐**14** crystals were used to superimpose both structures via the bispecific inhibitor (Figure S3). Due to the lack of an extensive linker in compound **14**, both lectins have insufficient space to simultaneously bind a single molecule of compound **14**. This finding supports the earlier discussion on the inability of bispecific inhibitors to bind LecA and LecB simultaneously. In summary, these results emphasize the potential of synthetic glycosides as promising candidates for developing new antimicrobial agents targeting pathogenic lectins, offering new strategies to combat *Pseudomonas aeruginosa* infections.

## Conclusions

In summary, in our study, we successfully synthesized hardly hydrolysable homo‐ and heterodisaccharides as mono‐ or bivalent inhibitors to *Pseudomonas aeruginosa* lectins. Specifically, we designed d‐galactose‐containing ligands with selectivity to LecA, l‐fucose‐containing ligands selective towards LecB, and additionally, bifunctional/bispecific Gal‐Fuc disaccharides. Through agglutination inhibition assays, we observed the inhibitory effects of these compounds on lectin‐induced agglutination and demonstrated the inhibitory activity of bispecific compounds against both LecA and LecB, showcasing their versatility in antimicrobial therapy. Isothermal titration calorimetry further confirmed the interaction between bispecific inhibitors and lectins, with increasing affinity observed. Moreover, X‐ray crystallography provided structural insights into their interactions. Experiments employing dynamic light scattering and analytical ultracentrifugation methods revealed that the bispecific inhibitor is incapable of binding both lectins simultaneously, a desirable property for potential utilization in anti‐adhesion therapy since such simultaneous binding could lead to precipitation. These findings highlight the promising role of synthetic glycosides in the development of novel antimicrobial agents targeting pathogenic lectins, offering new strategies for combating *P. aeruginosa* infections.

## Supporting Information Summary

The authors have cited additional references within the Supporting Information (Ref. [65–86]).

## Conflict of Interests

The authors declare no conflict of interest.

1

## Supporting information

As a service to our authors and readers, this journal provides supporting information supplied by the authors. Such materials are peer reviewed and may be re‐organized for online delivery, but are not copy‐edited or typeset. Technical support issues arising from supporting information (other than missing files) should be addressed to the authors.

Supporting Information

## Data Availability

The data that support the findings of this study are available in the supplementary material of this article.
