# Supplementary material for: Bispecific Thio‐Linked Disaccharides as Inhibitors of Pseudomonas Aeruginosa Lectins LecA (PA‐IL) and LecB (PA‐IIL): Dual‐Targeting Strategy
Source: Chemistry. 2024 Nov 28;31(5):e202403546. doi: 10.1002/chem.202403546 (PMC11753388; doi:10.1002/chem.202403546)
Supplement: Supplementary file 1 — Supporting Information [file CHEM-31-e202403546-s001.pdf]

Chemistry–A European Journal

Supporting Information

Bispecific Thio-Linked Disaccharides as Inhibitors of *Pseudomonas Aeruginosa* Lectins LecA (PA-IL) and LecB (PA-IIL): Dual-Targeting Strategy

Lukáš Faltinek, Filip Melicher, Viktor Kelemen, Erika Mező, Anikó Borbás,* and Michaela Wimmerová*

Bispecific Thio-linked Disaccharides as Inhibitors of *Pseudomonas aeruginosa* Lectins LecA (PA-IL) and LecB (PA-IIL): Dual-Targeting Strategy

Lukáš Faltinek, Filip Melicher, Viktor Kelemen, Erika Mező, Anikó Borbás, and Michaela Wimmerová

SUPPORTING INFORMATION

Materials and methods

1. Production of lectins

The LecA and LecB lectins in recombinant forms were produced and purified as previously described [1,2]. In summary, *Escherichia coli* cells transformed with a plasmid encoding the specific lectin were cultured in LB broth supplemented with an appropriate antibiotic at 37 °C. Upon reaching an OD_{600} of ≈0.5, the cells were induced with isopropyl 1-thio-β-D-galactopyranoside (IPTG) to a final concentration of 0.5 mM and further incubated at 30 °C for 3 hours. Subsequently, the cells were harvested by centrifugation, resuspended in a suitable buffer, and sonicated to disrupt the cells. The cytosolic fraction containing the soluble lectin was then separated by centrifugation. Lectins were purified using affinity chromatography, followed by dialysis and additional processing steps as outlined in previously published procedures. Freeze-dried lectins were stored at –20 °C.

2. Synthesis of glycan inhibitors

Chemistry – General information

1-Thio-β-L-fucopyranose (**1**) [3,4], 1-thio-β-D-glucopyranose (**2**) [5,6], 1-thio-β-D-galactopyranose (**3**) [7], 2,3,4,-tri-*O*-acetyl-1,5-anhydro-6-deoxy-L-*lyxo*-hex-1-enitol (**4**) [8], 2,3,4,6-tetra-*O*-acetyl-1,5-anhydro-D-*lyxo*-hex-1-enitol (**5**) [9], 2,3,4,6-tetra-*O*-acetyl-1,5-anhydro-D-*arabino*-hex-1-enitol (**6**) [9], 2,3,4,-tri-*O*-acetyl-1,5-anhydro-6-deoxy-D-*lyxo*-hex-1-enitol (**7**) [10], were prepared according to the literature methods. 2,2-Dimethoxy-2-phenylacetophenone (DPAP) and 4-methoxyacetophenone (MAP) were purchased from Sigma Aldrich Chemical Co., thioacetic acid (97%) was purchased from Alfa Aesar company. Optical rotations were measured at room temperature with a Perkin-Elmer 241 automatic polarimeter. TLC was performed on Kieselgel 60 F_{254} (Merck) with detection by UV-light (254 nm) and

immersing into sulfuric acidic ammonium-molibdenate solution or 5% ethanolic sulfuric acid followed by heating. Ellman's reagent was used for the detection of 1-thiosugars. Flash column chromatography was performed on Silica gel 60 (Merck 0.040-0.063 mm). Organic solutions were dried over Na_2SO_4 and concentrated under vacuum. ^{1}H and J-modulated ^{13}C NMR spectra were recorded with Bruker DRX-360 (^{1}H: 360 MHz; ^{13}C: 90 MHz), Bruker DRX-400 (^{1}H: 400 MHz; ^{13}C: 100 MHz) and Avance II 500 (^{1}H: 500 MHz; ^{13}C: 125 MHz) spectrometers at 25 ºC. Chemical shifts are referenced to Me_4Si (0.00 ppm for ^{1}H) and to the residual solvent signals ($CDCl_3$: 77.16, DMSO-d_6: 39.52, CD_3OD: 49.00 for ^{13}C). MALDI-ToF MS analyses of the compounds were carried out in the positive reflectron mode using a BIFLEX III mass spectrometer (Bruker, Germany) equipped with delayed-ion extraction. 2,5-Dihydroxybenzoic acid (DHB) was used as matrix and $F_3CCOONa$ as cationising agent in DMF. ESI-ToF HRMS spectra were recorded by a microToF-Q type QqToFMS mass spectrometer (Bruker) in the positive ion mode using MeOH as the solvent. The photoinduced reactions were carried out in a borosilicate vessel by irradiation with a 160 W Hg-lamp (Osram Supratec UV, HTC 150-211, 150 W, 230 V, R7s) giving maximum emission at 365 nm.

General method A for addition of 1-thiosugars to 2-OAc-glycals

2-Acetoxy glycal (1.0 equiv.), 1-thiosugar (1.2 equiv.) and DPAP (2,2-dimethoxy-2-phenylacetophenone, 0.1 equiv. / glycal) were dissolved in toluene (0.5 mmol glycal / 1 mL). The reaction mixture was cooled to –80 °C and was irradiated with UV light for 15 minutes. After irradiation, further 0.1 equiv. of DPAP was added and the irradiation was continued for 15 minutes. The addition of 0.1 equiv. of DPAP and the 15 minutes of irradiation was repeated one more time. The solvent was evaporated in vacuo and the crude product was purified by column chromatography.

General method B for addition of HSAc to 2-OAc-glycals

2-Acetoxy glycal (1.0 equiv.), thioacetic acid (6.0 equiv.), MAP (4-methoxyacetophenone, 0.3 equiv.) and DPAP (2,2-dimethoxy-2-phenylacetophenone, 0.1 equiv.) were dissolved in acetic acid (0.5 mmol glycal / 1 mL). The reaction flask was placed directly next to the UV lamp, at a distance of no 3-4 cm from the light source. The reaction mixture was cooled to –80 °C and was irradiated with UV light for 60 minutes. After irradiation, another 6.0 equiv. of HSAc and 0.1 equiv. of DPAP were added and the irradiation continued for 60 min. The addition of 6.0 equiv. of HSAc and 0.1 equiv. of DPAP, and the 60 min irradiation was repeated one more

time. The solvent was evaporated in vacuo and the crude product was purified by flash column chromatography.

General method C for selective *S*-deacetylation

Methanolic NaOMe stock solution was prepared by dissolving elemental sodium (2.3 g, 0.1 mol) in anhydrous methanol (100 mL). The S-acetyl derivative (1.0 equiv.) was dissolved in degassed methanol (1 mmol glycal / 10 mL solvent). The reaction mixture was cooled to 0 °C, stirred vigorously, and 0.95 equiv. of NaOMe was added in the form of the freshly prepared 1 M stock solution. The reaction mixture was stirred at 0 °C until no starting material was observed on TLC (approx. 30 min to 120 min). The solution was neutralised with Amberlite IR-120 H^+ ion exchange resin. The resin was filtered off, the solvent was evaporated in vacuo, the crude product was either used in the following reaction or was purified by flash column chromatography, and the thiol was stored under inert atmosphere until use.

General method D for *O*-deacetylation

The given acetyl-protected derivative was dissolved in dry methanol (1 mmol carbohydrate / 10 mL solvent). Catalytic amount of NaOMe was added (pH ~10). The reaction mixture was stirred at room temperature for 12 hours. The solution was neutralised with Amberlite IR-120 H^+ ion exchange resin. The resin was filtered off, the solvent was evaporated in vacuo and the crude product was purified by flash column chromatography.

2,3,4-Tri-O-acetyl-α-L-fucopyranosyl-2,3,4-tri-O-acetyl-1-thio-β-L-fucopyranoside (8)

2-Acetoxy-L-fucal **4** (272 mg, 1.0 mmol) and β-1-thiofucose **1** (306 mg, 1.2 mmol) and DPAP (25 mg, 0.1 mmol) were coupled according to the general method A. The crude product was purified by flash chromatography (hexane:acetone 8:2) to give compound **8** (420 mg, 73%) as a white powder. Physical properties are identical to the ones described in literature [11].

2,3,4-Tri-*O*-acetyl-α-L-fucopyranosyl-2,3,4,6-tetra-*O*-acetyl-1-thio-β-D-glucopyranoside (9)

2-Acetoxy-L-fucal **4** (200 mg, 0.735 mmol) and β-1-thioglucose **2** (308 mg, 0.882 mmol, 1.2 equiv.) were coupled according to the general method A. The crude product was purified by flash column chromatography (CH_2Cl_2:EtOAc 85:15) to give compound **9** (421 mg, 90%) as yellowish syrup. Physical properties are identical to the ones described in literature [11].

2,3,4-Tri-*O*-acetyl-α-L-fucopyranosyl-2,3,4,6-tetra-*O*-acetyl-1-thio-β-D-galactopyranoside (10)

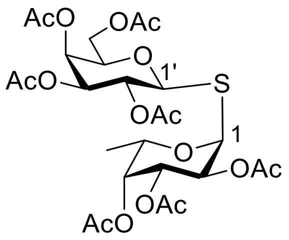

2-Acetoxy-L-fucal **4** (271 mg, 0.995 mmol) and β-1-thiogalactose **3** (446 mg, 1.224 mmol, 1.2 equiv.) were coupled according to the general method A. The crude product was purified using column chromatography (CH_2Cl_2:ethyl acetate 85:15) to give compound **10**, as colourless syrup (601 mg, 95%), $[\alpha]_D^{20}$= −150.1 (*c* 0.30, $CHCl_3$), R_f= 0.62 (CH_2Cl_2:acetone 95:5), ^{1}H NMR (400 MHz, $CDCl_3$) δ 5.88 (d, *J* = 5.6 Hz, 1H, H-1), 5.42 (d, *J* = 1.9 Hz, 1H, H-4'), 5.38 – 5.28 (m, 2H, H-4, H-2), 5.26 (dd, *J* = 10.1, 1.6 Hz, 1H, H-2'), 5.12 (dd, *J* = 10.9, 2.7 Hz, 1H, H-3), 5.07 (dd, *J* = 9.9, 3.3 Hz, 1H, H-3'), 4.67 (d, *J* = 10.2 Hz, 1H, H-1'), 4.37 (q, *J* = 6.3 Hz, 1H, H-5), 4.18 – 4.05 (m, 2H, H-6'$_a$, H-6'$_b$), 3.93 (t, *J* = 6.5 Hz, 1H, H-5'), 2.18 (s, 3H, AcCH_3), 2.16 (s, 3H, AcCH_3), 2.08 (s, 3H, AcCH_3), 2.07 (s, 3H, AcCH_3), 2.06 (s, 3H, AcCH_3), 1.99 (s, 3H, AcCH_3), 1.99 (s, 3H, AcCH_3), 1.19 (d, *J* = 6.4 Hz, 3H, H-6); ^{13}C NMR (101 MHz, $CDCl_3$) δ 170.4, 170.1, 169.9, 169.8 and 169.2 (7C, 7×Ac*C*O), 81.1 (C-1'), 80.3 (C-1), 74.5 (C-5'), 71.9 (C-3'), 70.5 (C-4), 68.5 (C-3), 67.2 and 67.2 (2C, C-2' and C-4'), 67.0 (C-2), 65.6 (C-5), 61.3 (C-6'), 20.7, 20.6, 20.6, 20.5 and 20.5 (7C, 7×Ac*C*H_3), 15.8 (C-6); MALDI-ToF-HRMS: m/z calcd for $C_{26}H_{36}NaO_{16}S$ $[M+Na]^+$ 659.1616, found 659.1613.

2,3,4,6-Tetra-*O*-acetyl-α-D-galactopyranosyl-2,3,4,6-tetra-*O*-acetyl-1-thio-β-D-galactopyranoside (11)

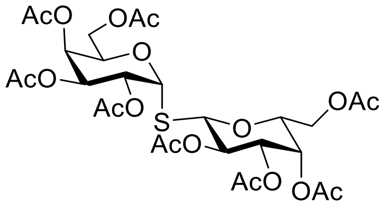

2-Acetoxy-D-galactal **5** (165 mg, 0.5 mmol) and β-1-thiogalactose **3** (218 mg, 0.6 mmol, 1.2 equiv.) were coupled according to the general method A. The crude product was purified using flash chromatography (*n*-hexane:acetone 75:25) to give compound **11**, as colourless syrup (303 mg, 87%). R_f = 0.23 (*n*-hexane:acetone 7:3), $[\alpha]_D^{20}$ = +137.9 (*c* 0.30, MeOH), ^{1}H NMR (500 MHz, $CDCl_3$) δ 5.99 (d, *J* = 5.1 Hz, 1H, H-1), 5.46 (dd, *J* = 3.1, 1.4 Hz, 1H, H-1'), 5.42 (dd, *J* = 3.3, 1.1 Hz, 1H), 5.27 (t, J = 10.0 Hz, 1H), 5.24 – 5.18 (m, 2H), 5.02 (dd, *J* = 10.0, 3.3 Hz, 1H), 4.65 – 4.49 (m, 2H), 4.21 – 4.12 (m, 2H), 4.12 – 4.01 (m, 2H), 4.01 – 3.90 (m, 1H), 2.17 (s, 3H, AcCH_3), 2.15 (s, 3H, AcCH_3), 2.05 (s, 3H, AcCH_3), 2.05 (s, 3H, AcCH_3), 2.04 (s, 6H,

2×AcCH_3), 2.00 (s, 3H, AcCH_3), 1.98 (s, 3H, AcCH_3) ppm. ^{13}C NMR (125 MHz, CDCl$_3$) δ 170.4, 170.3, 170.2, 170.1, 169.8, 169.3 (8C, Ac*C*O), 83.1, 82.8 (2C, C-1, C-1'), 75.0, 72.0, 68.5, 68.1, 67.4, 67.3, 67.2 (8C, skeletal carbons), 61.6, 60.5 (2C, C-6, C-6') ppm. MALDI-ToF-HRMS: *m/z* calcd for $C_{28}H_{38}NaO_{18}S$ $[M+Na]^+$ 717.1671 found 717.1661

α-L-Fucopyranosyl-1-thio-β-L-fucopyranoside (12)

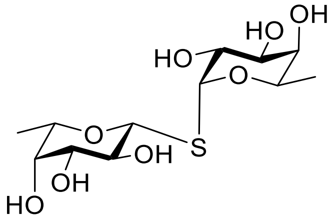

Compound **8** (244 mg, 0.34 mmol) was deprotected according to the general method D. The crude product was purified by flash column chromatography (acetonitrile:water 85:15) to give compound **12** (96 mg, 76%) as colourless crystals. R_f = 0.15 (CH_2Cl_2:MeOH 8:2) $[\alpha]_D^{20}$ = −179.3 (*c* 0.24, $CHCl_3$) mp.: 73−75 °C; ^{1}H NMR (360 MHz, MeOD + D_2O) δ 5.60 (d, *J* = 5.5 Hz, 1H, H-1), 4.44 – 4.38 (m, 1H), 4.36 (d, *J* = 9.6 Hz, 1H), 4.08 (dd, *J* = 10.0, 5.5 Hz, 1H), 3.74 – 3.63 (m, 4H), 3.60 (t, *J* = 9.5 Hz, 1H), 3.50 (dd, *J* = 9.3, 3.3 Hz, 1H), 1.26 (d, *J* = 6.4 Hz, 3H, 3×H-6), 1.22 (d, *J* = 6.5 Hz, 3H, 3×H-6) ppm. ^{13}C NMR (90 MHz, MeOD + D_2O) δ 88.0, 87.5 (2C, C-1, C-1'), 76.3, 76.1, 73.4, 73.1, 72.4, 71.8, 69.6, 68.9 (8C, skeletal carbons), 17.2, 16.6 (2C, C-6, C-6') ppm. MALDI-TOF-MS: *m/z* calcd for $C_{12}H_{22}NaO_8S$ $[M+Na]^+$ 349.0928, found 349.0922

α-L-Fucopyranosyl-1-thio-β-D-glucopyranoside (13)

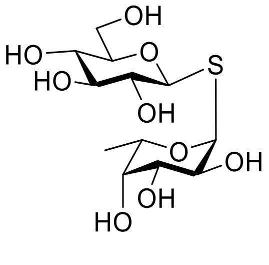

Compound **9** (150 mg, 0.24 mmol) was converted to **13** according to general method D. The crude product was purified by column chromatography (CH_2Cl_2:MeOH:H_2O 7:6:1) to give compound **13** (79 mg, 98%) as colorless syrup; R_f = 0.54 (CH_2Cl_2:MeOH:H_2O 7:6:1); $[\alpha]_D^{20}$ −178.2 (*c* 0.22, H_2O); ^{1}H NMR (400 MHz, D_2O) δ 5.64 (d, *J* = 5.6 Hz, 1H, H-1'), 4.48 (dd, *J* = 9.9, 1.2 Hz, 1H), 4.29 (q, *J* = 6.4 Hz, 1H), 4.05 (ddd, *J* = 10.4, 5.7, 1.0 Hz, 1H), 3.83 (d, *J* =

12.3 Hz, 1H), 3.75 (d, J = 3.3 Hz, 1H), 3.71–3.58 (m, 2H), 3.46–3.28 (m, 3H), 1.16 (d, J = 6.6 Hz, 3H, H-6’) ppm; ^{13}C NMR (101 MHz, D_2O) δ 83.0, 82.3 (2C, C-1, C-1’), 79.8, 77.2, 72.3, 71.6, 70.3, 69.5, 67.8, 67.2 (8C, skeleton carbons), 60.8 (1C, C-6), 15.3 (1C, C-6’) ppm; MALDI-ToF-HRMS: *m/z* calcd for $C_{12}H_{22}NaO_9S$ $[M+Na]^+$ 365.0877 found 365.0884.

α-L-Fucopyranosyl-1-thio-β-D-galactopyranoside (14)

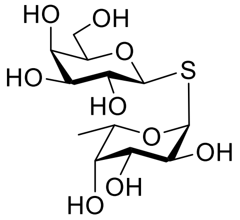

Compound **10** (100 mg, 0.16 mmol) was converted to **14** according to general method D. The crude product was purified by column chromatography (CH_2Cl_2:MeOH:H_2O 7:6:1) to give compound **14** (51 mg, 94%) as colorless syrup; R_f = 0.42 (CH_2Cl_2:MeOH:H_2O 7:6:1); $[\alpha]_D^{20}$ −211.0 (c 0.10, H_2O); ^{1}H NMR (400 MHz, D_2O) δ 5.75 (d, J = 5.7 Hz, 1H), 4.52 (d, J = 9.7 Hz, 1H), 4.40 (q, J = 6.5 Hz, 1H), 4.14 (dd, J = 10.4, 5.7 Hz, 1H), 3.99 (d, J = 2.4 Hz, 1H), 3.87–3.59 (m, 7H), 1.25 (d, J = 6.6 Hz, 3H, C-6’) ppm; ^{13}C NMR (101 MHz, D_2O) δ 83.1, 82.9 (2C, C-1, C-1’), 79.0, 73.9, 71.6, 70.3, 69.7, 68.8, 67.8, 67.2 (8C, skeleton carbons), 61.2 (1C, C-6), 15.4 (1C, C-6’) ppm; MALDI-ToF-HRMS: *m/z* calcd for $C_{12}H_{22}NaO_9S$ $[M+Na]^+$ 365.0877 found 365.0884.

α-D-Galactopyranosyl-1-thio-β-D-galactopyranoside (15)

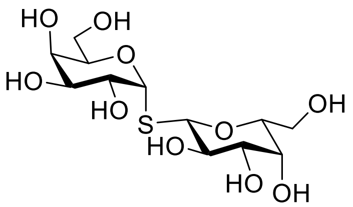

Compound **11** (250 mg, 0.36 mmol) was deprotected according to the general method D. The crude product was purified by flash column chromatography (acetonitrile:water 85:15) to give compound **15** (100 mg, 77%) as colourless foam. R_f = 0.30 (acetonitrile:water 7:3), $[\alpha]_D^{20}$ +194.3 (c 0.35, H_2O); ^{1}H NMR (500 MHz, MeOD + D_2O) δ 5.78 (d, J = 5.6 Hz, 1H, H-1_{alpha}), 4.41 – 4.36 (m, 2H, H-1_{beta}, and skeleton proton), 4.10 (dd, J = 10.2, 5.6 Hz, 1H, H-2_{alpha}), 3.90 – 4.36 (m, 2H), 3.87 – 3.77 (m, 3H), 3.73 (dd, J = 11.4, 4.0 Hz, 1H), 3.68 (dd, J = 11.5, 4.6 Hz, 1H), 3.64 – 3.59 (m, 2H, H-3_{alpha} and skeleton proton), 3.50 (dd, J = 9.4, 3.3 Hz, 1H) ppm.

^{13}C NMR (125 MHz, MeOD + D_2O) δ 86.7, 86.1 (2C, C-1, C-1'), 81.0, 76.1, 73.6, 72.3, 71.5, 71.0, 70.7, 69.6 (4C, skeletal carbons), 63.0, 62.9 (2C, C-6, C-6') ppm. MALDI-ToF-HRMS: *m/z* calcd for $C_{12}H_{22}NaO_{10}S$ $[M+Na]^+$ 381.0826 found 381.0825

2,3,4-Tri-*O*-acetyl-1-*S*-acetyl-1-thio-α-L-fucopyranose (16-SAc)

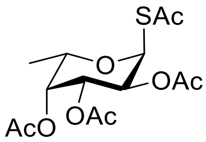

L-Fucal **4** (272 mg, 1.0 mmol), thioacetic acid (6.0 equiv, 6.0 mmol, 0.42 mL), MAP (0.3 equiv., 0.3 mmol, 45 mg), and DPAP (0.1 equiv, 0.1 mmol, 25 mg) were dissolved in acetic acid (1.5 mL). The reaction mixture was irradiated with UV light for 60 minutes at -80 °C. After irradiation, another 6.0 equiv. of thioacetic acid, and 0.1 equiv. of DPAP were added and the irradiation was continued for another 60 min. After irradiation, another 6.0 equiv. of thioacetic acid, and 0.1 equiv. of DPAP were added and the irradiation was continued for another 60 min. The solvent was evaporated in vacuo and the crude product was purified by flash column chromatography (CH_2Cl_2:acetone 98:2) to give compound **16-SAc** (315 mg, 91%) as white crystals. $R_f = 0.32$ (CH_2Cl_2:acetone 98:2) $[\alpha]_D^{20} = -132.5$ (*c* 2.0 in $CHCl_3$), lit. [12] -156.0; m. p.: 121−123°C, lit. [12] 119−120°C; ^1H NMR (400 MHz, $CDCl_3$) δ 6.23 (d, *J* = 5.5 Hz, 1H, H-1), 5.47 (dd, *J* = 11.0. 5.4 Hz, 1H, H-2), 5.29 (dd, *J* = 3.3, 1.3 Hz, 1H, H-4), 5.05 (dd, *J* = 11.0. 3.3 Hz, 1H, H-3), 4.06 (dd, *J* = 6.4, 1.3 Hz, 1H, H-5), 2.42 (s, 3H, SAcC*H*3), 2.18 (s, 3H, OAcC*H*3), 2.01 (s, 3H, OAcC*H*3), 2.00 (s, 3H, OAcC*H*3), 1.15 (d, *J* = 6.4 Hz, 3H, 3×H-6) ppm, ^{13}C NMR (100 MHz, $CDCl_3$) δ 192.3 (1C, SAc*C*O), 170.6, 170.0. 169.7 (3C, 3xOAc, *C*O), 81.3, 70.5, 69.4, 69.2, 66.5 (4C, skeletal carbons), 31.6 (1C, SAc*C*H3), 20.8, 20.7 (3C, 3xOAc, *C*H3), 16.2 (1C, C-6) ppm. MALDI-ToF-HRMS: *m/z* calcd for $C_{14}H_{20}NaO_8S$ $[M+Na]^+$ 371.0771, found 371.0740

2,3,4,6-Tetra-*O*-acetyl-1-*S*-acetyl-1-thio-α-D-glucopyranose (17-SAc)

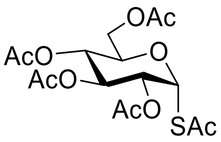

D-Glucal **6** (330 mg, 1.0 mmol), thioacetic acid (6.0 equiv, 6.0 mmol, 0.42 mL), DPAP (0.1 equiv, 0.1 mmol, 25 mg) and MAP (0.3 equiv, 0.3 mmol, 45 mg), were dissolved in acetic acid (1.5 mL). The reaction mixture was cooled to -80 °C and was irradiated with UV light for 60 minutes. After irradiation, another 6.0 equiv. of thioacetic acid and 0.1 equiv. of DPAP were

added and the irradiation was continued for 60 min. The addition of 6.0 equiv. of thioacetic acid and 0.1 equiv. of DPAP, and the 60 min irradiation was repeated one more time. The solvent was evaporated in vacuo and the crude product was purified by flash column chromatography (*n*-hexane:acetone 85:15) to give compound **17-SAc** (328 mg, 81%) as white crystals.

R_f = 0.20 (*n*-hexane:acetone 8:2) $[\alpha]_D^{20}$= +132.8 (*c* 0.28 in $CHCl_3$), lit. [13] +135; m. p.: 128−129 °C, lit. [13] m.p.: 125 °C; ^{1}H NMR (400 MHz, $CDCl_3$) δ 6.22 (d, *J* = 5.2 Hz, 1H, H-1), 5.24 (dd, *J* = 10.1, 5.1 Hz, 1H, H-2), 5.18 (t, *J* = 9.5 Hz, 1H, H-3), 5.10 (t, *J* = 9.5 Hz, 1H, H-4), 4.28 (dd, *J* = 12.5, 4.1 Hz, 1H, H-6a), 4.05 (dd, *J* = 12.5, 2.2 Hz, 1H, H-6b), 3.96 (ddd, *J* = 9.9, 4.0, 2.3 Hz, 1H, H-5), 2.43 (s, 3H, AcC*H*3), 2.08 (s, 3H, AcC*H*3), 2.03 (s, 3H, AcC*H*3), 2.02 (s, 3H, AcC*H*3), 2.02 (s, 3H, AcC*H*3) ppm. ^{13}C NMR (100 MHz, $CDCl_3$) δ 170.7, 170.1, 169.5, 169.4 (4C, 4xAc*C*O), 80.5 (1C, C-1), 71.6, 71.3, 69.2, 68.0 (4C, skeletal carbons), 61.7 (1C, C-6), 31.6 (1C, SAc*C*H3), 20.8, 20.7, 20.7 (4C, 4xOAc*C*H3) ppm.; ESI-HRMS: *m/z* calcd for $C_{16}H_{22}NaO_{10}S$ $[M+Na]^+$ 429.0826, found 429.0826

2,3,4,6-Tetra-*O*-acetyl-1-*S*-acetyl-1-thio-α-D-galactopyranose (18-SAc)

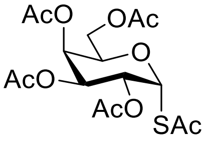

D-Galactal **5** (330 mg, 1.0 mmol), thioacetic acid (6.0 equiv, 6.0 mmol, 0.42 mL), MAP (0.3 equiv., 0.3 mmol, 45 mg) and DPAP (0.1 equiv, 0.1 mmol, 25 mg) were dissolved in acetic acid (1.5 mL). The reaction mixture was cooled to -80 °C and was irradiated with UV light for 60 minutes. After irradiation, another 6.0 equiv. of thioacetic acid and 0.1 equiv. of DPAP were added and the irradiation was continued for 60 min. The addition of 6.0 equiv. of thioacetic acid and 0.1 equiv. of DPAP, and the irradiation was repeated one more time. The solvent was evaporated in vacuo and the crude product was purified by flash column chromatography (dichloromethane:acetone 98:2) to give compound **20** (365 mg, 90%) as white crystals. R_f = 0.28 (CH_2Cl_2:acetone 98:2); $[\alpha]_D^{20}$= +157.1 (*c* 3.50 in $CHCl_3$), lit. [14] +120.9; m.p.: 118−121°C, lit. [14] m.p.: 119−121°C. ^{1}H NMR (400 MHz, $CDCl_3$) δ 6.27 (d, *J* = 5.4 Hz, 1H, H-1), 5.48 (dd, *J* = 11.0, 5.5 Hz, 1H, H-2), 5.44 (d, *J* = 3.1 Hz, 1H, H-4), 5.04 (dd, *J* = 11.0. 3.3 Hz, 1H, H-3), 4.22 – 4.13 (m, 1H, H-5), 4.14 – 4.03 (m, 2H, H-6a,b), 2.43 (s, 3H, SAc*C*H3), 2.16 (s, 3H, OAc*C*H3), 2.03 (s, 3H, OAc*C*H3), 2.02 (s, 3H, OAc*C*H3), 1.99 (s, 3H, OAc*C*H3) ppm. ^{13}C NMR (100 MHz, $CDCl_3$) δ 191.8 (1C, SAc*C*O), 170.4, 170.2, 170.0. 169.7 (4C, 4xOAc*C*O), 81.2

(1C, C-1), 70.3, 68.9, 67.3, 66.4 (4C, skeletal carbons), 61.2 (1C, C-6), 31.6 (1C, SAc*C*H_3), 20.7 (4C, 4xOAc*C*H_3) ppm. MALDI-ToF-MS: *m/z* calcd for $C_{16}H_{22}NaO_{10}S$ $[M+Na]^+$ 429.0826, found 429.0682.

2,3,4-Tri-*O*-acetyl-1-thio-α-L-fucopyranose (16)

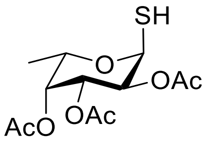

Compound **16-SAc** (348 mg, 1.0 mmol) was selectively deacetylated according to the general method C. The crude product was purified by flash column chromatography (CH_2Cl_2:acetone 98:2) to give compound **16** (275 mg, 90%) as colourless foam. R_f = 0.24 (CH_2Cl_2:acetone 98:2) $[\alpha]_D^{20}$= −189.6 (*c* 0.26 in $CHCl_3$), lit.[12] −162. ^{1}H NMR (400MHz, $CDCl_3$) δ 97 (t, *J* = 4.5 Hz, 1H, H-1), 5.31 (d, *J* = 1.2 Hz, 1H, H-4), 5.27 – 5.24 (m, 2H, H-2 and H-3), 4.53 (q, *J* = 6.5 Hz, 1H, H-5), 2.17 (s, 3H, AcC*H*$_3$), 2.09 (s, 3H, AcC*H*$_3$), 2.00 (s, 3H, AcC*H*$_3$), 1.80 (d, *J* = 5.1 Hz, 1H, S*H*), 1.16 (d, *J* = 6.5 Hz, 3H, 3×H-6) ppm. ^{13}C NMR (100 MHz, $CDCl_3$) *δ* 170.6, 170.1 (3C, 3xAc*C*O), 77.8 (1C, C-1), 70.9, 68.1, 67.7, 65.6 (4C, skeletal carbons), 20.9, 20.8, 20.7 (3C, 3xAc*C*H_3), 16.0 (1C, C-6) ppm. MALDI-ToF-HRMS: *m/z* calcd for $C_{12}H_{18}NaO_7S$ $[M+Na]^+$329.0665, found 329.0643.

2,3,4,6-Tetra-*O*-acetyl-1-thio-α-D-glucopyranose (17)

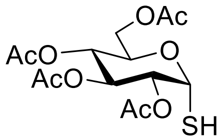

Compound **17-SAc** (406 mg, 1.0 mmol) was selectively deacetylated according to the general method C. The crude product was purified by flash column chromatography (*n*-hexane:acetone 8:2) to give compound **17** (320 mg, 88%) as colourless foam. R_f = 0.26 (*n*-hexane:acetone 8:2), $[\alpha]_D^{20}$= + 170.2 (*c* 0.3 in $CHCl_3$), lit. [15] +168. ^{1}H NMR (400 MHz, $CDCl_3$) δ 5.94 (t, *J* = 5.4 Hz, 1H, H-1), 5.38 (t, *J* = 9.8 Hz, 1H, H-3), 5.14 – 4.98 (m, 2H, H-2, H-4), 4.50 – 4.40 (m, 1H, H-5), 4.30 (dd, *J* = 12.4, 4.2 Hz, 1H, H-6a), 4.11 (dd, *J* = 12.4, 2.3 Hz, 1H, H-6b), 2.09 (s, 6H 2x AcC*H*$_3$), 2.04 (s, 6H, 2x AcC*H*$_3$), 1.98 – 1.90 (m,1H, S*H*) ppm. ^{13}C NMR (100 MHz, $CDCl_3$) δ 170.7, 170.1, 169.8, 169.6 (4C, 4xAc*C*O), 77.2 (1C, C-1), 70.4 (1C, C-2), 70.0 (1C, C-3),

68.4 (2C, C-4, C-5), 61.8 (1C, C-6), 20.8, 20.8, 20.7, 20.7 (4C, 4xAcCH_3) ppm. MALDI-ToF-HRMS: *m/z* calcd for $C_{14}H_{20}NaO_9S$ $[M+Na]^+$ 387.0720, found 387.0710.

2,3,4,6-Tetra-*O*-acetyl-1-thio-α-D-galactopyranose (18)

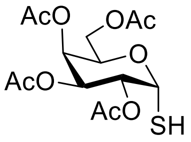

Compound **18-SAc** (230 mg, 0.56 mmol) was selectively deacetylated according to the general method C. The crude product was purified by flash column chromatography (*n*-hexane:acetone 8:2) to give compound **18** (180 mg, 90%) as colourless foam. $R_f = 0.23$ (*n*-hexane:acetone 8:2); $[\alpha]_D^{20}$= +181.0 (*c* 0.2 in $CHCl_3$), lit. [16] +172. ^{1}H NMR (400 MHz, $CDCl_3$) *δ* 6.02 (t, *J* = 5.0 Hz, 1H, H-1), 5.49 – 5.45 (m, 1H, H-4), 5.33 – 5.19 (m, 2H, H-2, H-3), 4.62 (t, *J* = 6.6 Hz, 1H, H-5), 4.15 (dd, *J* = 11.3, 6.4 Hz, 1H, H-6a), 4.07 (dd, *J* = 11.3, 6.7 Hz, 1H, H-6b), 2.15 (s, 3H, AcCH_3), 2.09 (s, 3H, AcCH_3), 2.06 (s, 3H, AcCH_3), 2.01 (s, 3H, AcCH_3), 1.86 (d, *J* = 5.2 Hz, 1H, S*H*) ppm. ^{13}C NMR (100 MHz, $CDCl_3$) δ 170.4, 170.1, 170.0, 169.9 (4C, 4x AcCO), 77.8 (1C, C-1), 67.8 (1C, C-4), 67.6 (1C, C-2), 67.5 (1C, C-3), 67.2 (1C, C-5), 61.5 (1C, C-6), 20.8, 20.7, 20.7, 20.7 (4C, 4x AcCH_3) ppm. MALDI-ToF-HRMS: *m/z* calcd for $C_{14}H_{20}NaO_9S$ $[M+Na]^+$ 387.0720, found 387.099.

2,3,4-Tri-O-acetyl-α-L-fucopyranosyl 2,3,4-tri-*O*-acetyl-1-thio-α-L-fucopyranoside (19)

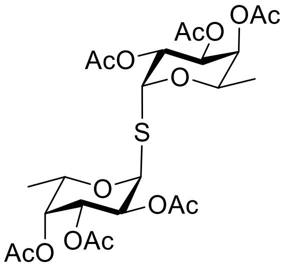

L-Fucal **4** (136 mg, 0.5 mmol) and 1-thio-L-fucose **16** (1.2 equiv, 184 mg, 0.6 mmol) were reacted in a toluene:CH_2Cl_2 3:1 mixture (1 mL) according to the general method A. The crude product was purified by flash column chromatography (*n*-hexane:acetone 85:15) to give compound **19** (201 mg, 71%) as white crystals. $R_f = 0.23$ (*n*-hexane:acetone 8:2); $[\alpha]_D^{20}$= -265.6 (*c* 0.34 in $CHCl_3$), m.p.: 82−85°C; ^{1}H NMR (400 MHz, $CDCl_3$) δ 5.85 (d, *J* = 5.6 Hz, 1H, H-1), 5.30 (dd, *J* = 3.3, 1.2 Hz, 1H), 5.30 – 5.22 (m, 1H), 5.17 (dd, *J* = 10.8, 3.3 Hz, 1H), 4.31 (q,

J = 7.0. 6.5 Hz, 1H), 2.17 (d, J = 3.4 Hz, 3H, AcCH_3), 2.10 (s, 3H, AcCH_3), 2.01 (s, 3H, AcCH_3), 1.15 (d, J = 6.5 Hz, 3H, H-6) ppm. ^{13}C NMR (100 MHz, $CDCl_3$) δ 170.6, 170.1, 170.0 (3C, 3×Ac*C*O), 79.5 (1C, C-1), 70.8, 69.0. 67.8, 65.6 (4C, skeletal carbons), 20.9, 20.8, 20.7 (3C, 3×Ac*C*H_3), 15.9 (1C, C-6) ppm. MALDI-ToF-HRMS: *m/z* calcd for $C_{24}H_{34}NaO_{14}S$ $[M+Na]^+$ 601.1561, found 601.1548.

2,3,4,6-Tetra-*O*-acetyl-α-D-glucopyranosyl 2,3,4-tri-*O*-acetyl-1-thio-α-L-fucopyranoside (20)

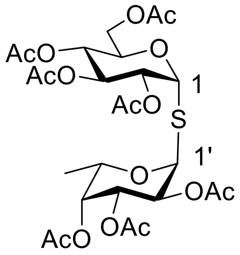

L-Fucal **4** (68 mg, 0.25 mmol) and 1-thioglucose **17** (1.2 equiv., 109 mg, 0.3 mmol) were reacted in a 3:1 toluene:CH_2Cl_2 mixture (0.8 mL) according to the general method A. The crude product was purified by flash column chromatography (*n*-hexane:acetone 7:3) to give compound **20** (120 mg, 75%) as colourless syrup. R_f = 0.27 (*n*-hexane:acetone 7:3); $[\alpha]_D^{20}$= +27.5 (*c* 0.12 in $CHCl_3$); ^{1}H NMR (400 MHz, $CDCl_3$) δ 5.72 (d, J = 5.2 Hz, 1H, H-1*), 5.70 (d, J = 5.6 Hz, 1H, H-1'*), 5.34 (t, J = 9.8 Hz, 1H), 5.30 (d, J = 2.9 Hz, 1H), 5.24 (dd, J = 10.9, 5.2 Hz, 1H), 5.19 (dd, J = 10.9, 3.1 Hz, 1H), 5.07 (t, J = 9.8 Hz, 1H), 5.00 (dd, J = 10.3, 5.6 Hz, 1H), 4.32 – 4.27 (m, 2H), 4.23 – 4.20 (m, 1H), 4.05 (dd, J = 12.4, 2.1 Hz, 1H), 2.17 (s, 3H, AcCH_3), 2.09 (s, 3H, AcCH_3), 2.06 (s, 3H, AcCH_3), 2.05 (s, 3H, AcCH_3), 2.05 (s, 3H, AcCH_3), 2.03 (s, 3H, AcCH_3), 2.01 (s, 3H, AcCH_3), 1.14 (d, J = 6.4 Hz, 3H, 3×H-6') ppm, ^{13}C NMR (100 MHz, $CDCl_3$) δ 170.7, 170.6, 170.2, 170.0, 170.0, 169.9, 169.7 (7C, Ac*C*O), 83.8, 82.9 (2C, C-1, C-1'), 70.9, 70.7, 70.6, 69.5, 68.7, 68.2, 68.1, 66.8 (8C, skeletal carbons), 61.8 (1C, C-6), 20.8, 20.7 (7C, Ac*C*H_3), 16.1 (1C, C-6') ppm. MALDI-ToF-HRMS: *m/z* calcd for $C_{26}H_{36}NaO_{16}S$ $[M+Na]^+$ 659.1616, found 659.1624.

2,3,4,6-Tetra-*O*-acetyl-α-D-galactopyranosyl 2,3,4-tri-*O*-acetyl-1-thio-α-L-fucopyranoside (21)

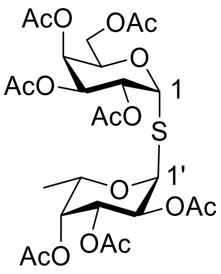

L-Fucal **4** (69 mg, 0.25 mmol) and 1-thiogalactose **18** (1.2 equiv, 110 mg, 0.3 mmol) were reacted in a toluene:CH_2Cl_2 3:1 mixture (0.8 mL) according to the general method A. The crude product was purified by flash column chromatography (*n*-hexane:acetone 75:25) to give compound **21** (78 mg, 82%) as white crystals. R_f = 0.26 (*n*-hexane:acetone 7:3); $[\alpha]_D^{20}$= +21.5 (*c* 3.9 in $CHCl_3$); m.p.: 66−68°C. ^{1}H NMR (400 MHz, $CDCl_3$) δ 5.77 (d, *J* = 5.1 Hz, 1H, H-1), 5.71 (d, *J* = 5.1 Hz, 1H, H-1'), 5.46 (d, *J* = 2.2 Hz, 1H), 5.30 (d, *J* = 1.9 Hz, 1H), 5.28 – 5.18 (m, 4H), 4.41 (t, *J* = 6.4 Hz, 1H), 4.30 (q, *J* = 6.2 Hz, 1H), 4.16 (dd, *J* = 11.3, 6.8 Hz, 1H), 4.01 (dd, *J* = 11.2, 6.3 Hz, 1H), 2.16 (s, 3H, AcC*H*3), 2.14 (s, 3H, AcC*H*3), 2.07 (s, 3H, AcC*H*3), 2.06 (s, 3H, AcC*H*3), 2.04 (s, 3H, AcC*H*3), 2.01 (s, 6H, 2xAcC*H*3), 1.15 (d, *J* = 6.4 Hz, 3H, 3xH-6) ppm. ^{13}C NMR (125 MHz, $CDCl_3$) δ 170.5, 170.3, 170.2, 170.1, 170.0. 169.9 (7C, 7xAc*C*O), 83.4 (2C, C-1, C-1'), 70.7, 68.7, 68.2, 68.1, 68.0, 67.6, 66.8 (8C, skeletal carbons), 61.2 (1C, C-6), 20.7 (7C, 7xAc*C*H3), 16.0 (1C, C-6') ppm. MALDI-ToF-HRMS: *m/z* calcd for $C_{26}H_{36}NaO_{16}S$ $[M+Na]^+$ 659.1616, found 659.1626.

2,3,4-tri-*O*-acetyl-6-deoxy-α-D-galactopyranosyl 2,3,4,6-tetra-*O*-acetyl-1-thio-α-D-galactopyranoside (22)

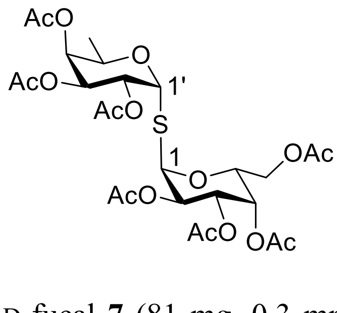

D-fucal **7** (81 mg, 0.3 mmol) and 1-thiogalactose **18** (1.2 equiv, 131 mg, 0.36 mmol) were reacted in a 3:1 toluene-CH_2Cl_2 mixture(1 mL) according to the general method A. The crude product was purified by flash column chromatography (*n*-hexane:acetone 75:25) to give compound **22** (164, 86%) as colourless foam. R_f = 0.28 (*n*-hexane:acetone 7:3), $[\alpha]_D^{20}$= +253.3 (*c* 0.12 in $CHCl_3$). ^{1}H NMR (360 MHz, $CDCl_3$) δ 5.92 (d, *J* = 5.6 Hz, 1H, H-1), 5.82 (d, *J* = 5.6 Hz, 1H, H-1'), 5.44 (d, *J* = 2.3 Hz, 1H), 5.37 – 5.27 (m, 2H), 5.26 – 5.10 (m, 3H), 4.52 – 4.38

(m, 1H), 4.30 (q, J = 6.3 Hz, 1H), 4.08 (ddd, J = 18.9, 11.5, 6.4 Hz, 2H), 2.17 (s, 3H, AcCH_3), 2.14 (s, 3H, AcCH_3), 2.11 (s, 6H, 2xAcCH_3), 2.09 (s, 3H, AcCH_3), 2.02 (s, 3H, AcCH_3), 2.01 (s, 3H, AcCH_3), 1.15 (d, J = 6.5 Hz, 3H, 3xH-6') ppm. ^{13}C NMR (90 MHz, $CDCl_3$) δ = 170.1, 169.9 (7C, 7xAc*C*O), 79.4, 79.3 (2C, C-1, C-1'), 70.6, 68.8, 68.4, 67.8, 67.8, 67.4, 67.4, 65.6 (8C, skeletal carbons), 62.0 (1C, C-6), 20.7 (7C, 7xAc*C*H_3), 15.8 (1C, C-6') ppm. MALDI-ToF-HRMS: *m/z* calcd for $C_{26}H_{36}NaO_{16}S$ $[M+Na]^+$ 659.1616, found 659.1631.

2,3,4,6-Tetra-*O*-acetyl-α-D-galactopyranosyl 2,3,4,6-tetra-*O*-acetyl-1-thio-α-D-galactopyranoside (23)

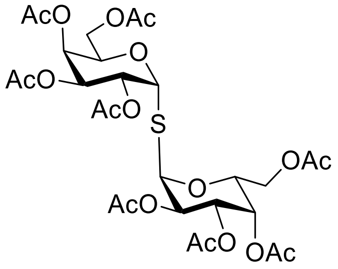

D-Galactal **5** (60 mg, 0.18 mmol) and 1-thiogalactose **18** (1.2 equiv, 80 mg, 0.22 mmol) were reacted according to the general method A. The crude product was purified by flash column chromatography (*n*-hexane:acetone 7:3) to give compound **23** (100 mg, 80%) as colourless syrup. R_f = 0.20 (*n*-hexane:acetone 7:3), $[\alpha]_D^{20}$= +274.2 (*c* 2.4 in $CHCl_3$). ^{1}H NMR (400 MHz, $CDCl_3$) δ 5.87 (d, J = 5.7 Hz, 1H, H-1), 5.45 (d, J = 2.8 Hz, 1H), 5.32 (dd, J = 10.9, 5.7 Hz, 1H, H-2), 5.18 (dd, J = 10.9, 3.3 Hz, 1H, H-3), 4.46 (t, J = 6.6 Hz, 1H), 4.17 – 3.99 (m, 2H, H-6a,b), 2.15 (s, 3H, AcCH_3), 2.12 (s, 3H, AcCH_3), 2.09 (s, 3H, AcCH_3), 2.02 (s, 3H, AcCH_3) ppm, ^{13}C NMR (100 MHz, $CDCl_3$) δ 170.7, 170.1, 170.0, 169.9 (4C, 4xAc*C*O), 79.1 (1C, C-1), 68.3, 67.7, 67.4, 67.2 (4C, skeletal carbons), 61.9 (1C, C-6), 20.8, 20.7, 20.6, 20.6 (4C, 4xAc*C*H_3) ppm. MALDI-ToF-HRMS: *m/z* calcd for $C_{28}H_{38}NaO_{18}S$ $[M+Na]^+$ 717.1671, found 717.231.

2,3,4,6-Tetra-*O*-acetyl-α-D-glucopyranosyl 2,3,4,6-tetra-*O*-acetyl-1-thio-α-D-galactopyranoside (24)

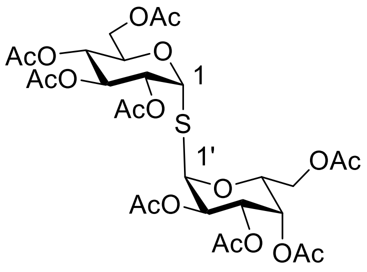

D-Galactal **5** (90 mg, 0.27 mmol) and 1-thioglucose **17** (1.2 equiv, 120 mg, 0.32 mmol) were reacted according to the general method A. The crude product was purified by flash column chromatography (*n*-hexane:acetone 7:3) to give compound **24** (175 mg, 95%) as colourless syrup. R_f = 0.23 (*n*-hexane:acetone 7:3) $[\alpha]_D^{20}$= +261.5 (*c* 0.41 in $CHCl_3$) ^{1}H NMR (400 MHz, $CDCl_3$) δ 5.89 (d, *J* = 5.6 Hz, 1H, H-1*), 5.82 (d, *J* = 5.8 Hz, 1H, H-1'*), 5.44 (d, *J* = 2.8 Hz, 1H), 5.35 (t, *J* = 9.8 Hz, 1H), 5.29 (dd, *J* = 10.9, 5.6 Hz, 1H), 5.18 (dd, *J* = 10.9, 3.3 Hz, 1H), 5.09 (dd, *J* = 10.2, 5.9 Hz, 1H), 5.03 (t, *J* = 9.6 Hz, 1H), 4.43 (t, *J* = 6.6 Hz, 1H), 4.31 – 4.20 (m, 2H), 4.17 – 4.00 (m, 3H), 2.15 (s, 3H, AcC*H*3), 2.12 (s, 6H, 2xAcC*H*3), 2.11 (s, 3H, AcC*H*3), 2.07 (s, 3H, AcC*H*3), 2.05 (s, 3H, AcC*H*3), 2.04 (s, 3H, AcC*H*3), 2.02 (s, 3H, AcC*H*3) ppm, *interchangeable signals. ^{13}C NMR (100 MHz, $CDCl_3$) δ 170.9, 170.7, 170.2, 170.1, 170.0. 169.7, 169.6 (8C, 8x*C*O, Ac), 79.1, 78.7 (2C, C-1, C-1'), 70.6, 70.1, 68.6, 68.5, 68.3, 67.7, 67.5 (8C, skeletal carbons), 61.9, (2C, C-6, C-6'), 20.9, 20.7, 20.7 (8C, 2xAc*C*H3) ppm. MALDI-ToF-HRMS: *m/z* calcd for $C_{28}H_{38}NaO_{18}S$ $[M+Na]^+$ 717.1671 found 717.1668.

α-L-Fucopyranosyl 1-thio-α-L-fucopyranoside (25)

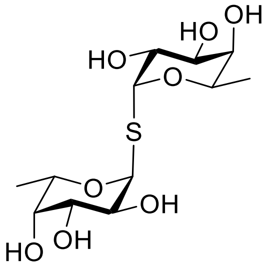

Compound **19** (289 mg, 0.5 mmol) in MeOH (10 mL) was deacetylated according to the general method D. The crude product was purified by flash column chromatography (acetonitrile:water 8:2) to give compound **25** (155 mg, 95%) as colourless syrup. R_f = 0.26 (acetonitrile:water 85:15), $[\alpha]_D^{20}$= −42.8 (*c* 0.13 in DMSO). ^{1}H NMR (400 MHz, DMSO-*d*6 + D_2O) δ 5.48 (d, *J* = 5.6 Hz, 1H, H-1), 4.41 (q, *J* = 6.2 Hz, 1H, H-5), 4.14 (dd, *J* = 10.3, 5.6 Hz, 1H, H-2), 3.85 (dd, *J* = 3.4, 1.1 Hz, 1H, H-4), 3.77 (dd, *J* = 10.3, 3.4 Hz, 1H, H-3), 1.29 (d, *J* = 6.5 Hz, 3H, 3xH-6) ppm. ^{13}C NMR (100 MHz, DMSO-*d*6 + D_2O) δ 84.2 (1C, C-1), 73.0. 72.0. 69.0. 68.7 (4C, skeletal carbons), 17.1 (1C, *C*-6) ppm. MALDI-ToF-HRMS: *m/z* calcd for $C_{12}H_{22}NaO_8S$ $[M+Na]^+$ 349.0928, found 349.0943.

α-D-Glucopyranosyl 1-thio-α-L-fucopyranoside (26)

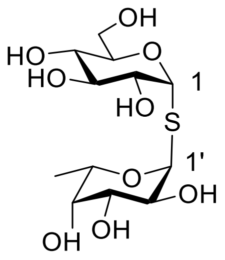

Compound **20** (83 mg, 0.135 mmol) was deprotected according to the general method D. The crude product was purified by flash column chromatography (acetonitrile:water 8:2) to give compound **26** (45 mg, 96%) as colourless foam. R_f = 0.35 (acetonitrile:water 75:25), $[\alpha]_D^{20}$= −27.9 (*c* 0.14 in DMSO). ^{1}H NMR (400 MHz, MeOD) δ 5.53 (d, *J* = 5.5 Hz, 1H, H-1), 5.47 (d, *J* = 5.4 Hz, 1H, H-1'), 4.27 (q, *J* = 6.4 Hz, 1H), 4.09 (dd, *J* = 10.4, 5.5 Hz, 1H), 3.95 – 3.71 (m, 6H), 3.56 (t, *J* = 9.3 Hz, 1H), 3.49 (t, *J* = 9.3 Hz, 1H), 1.22 (d, *J* = 6.5 Hz, 3H, 3xH-6') ppm. ^{13}C NMR (100 MHz, MeOD) δ 89.0. 88.5 (2C, C-1, C-1'), 75.0. 74.2, 72.6, 72.2, 71.4, 70.1, 69.3, 68.9 (8C, skeletal carbons), 61.1 (1C, C-6), 16.4 (1C, C-6') ppm. MALDI-ToF-HRMS: *m/z* calcd for $C_{12}H_{22}NaO_9S$ [M+Na]$^+$ 365.0877 found 365.0846.

α-D-Galactopyranosyl 1-thio-α-L-fucopyranoside (27)

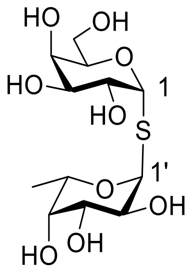

Compound **21** (127 mg, 0.2 mmol) was deprotected according to the general method D. The crude product was purified by flash column chromatography (acetonitrile:water 8:2) to give compound **27** (65 mg, 96%) as colourless foam. R_f = 0.34 (acetonitrile:water 7:3), $[\alpha]_D^{20}$= +70.0 (*c* 0.12 in H_2O). ^{1}H NMR (360 MHz, DMSO-*d*$_6$) δ 5.25 (t, *J* = 5.8 Hz, 2H, H-1, H-1'), 4.94 (s, 2H), 4.72 (s, 2H), 4.49 (s, 2H), 4.00 (d, *J* = 6.6 Hz, 2H), 3.97 – 3.69 (m, 5H), 3.68 – 3.48 (m, 4H), 1.09 (d, *J* = 6.4 Hz, 3H, H-6') ppm. ^{13}C NMR (90 MHz, DMSO-*d*$_6$) δ = 87.5, 87.4 (2C, C-1, C-1'), 72.5, 71.6, 70.9, 68.3, 68.2, 68.1, 68.0 (8C, skeletal carbons), 59.9 (1C, C-6), 16.6 (1C, C-6') ppm. MALDI-ToF-HRMS: *m/z* calcd for $C_{12}H_{22}NaO_9S$ [M+Na]$^+$ 365.0887 found 381.0856.

6-Deoxy-α-D-galactopyranosyl-1-thio-α-D-galactopyranoside (28)

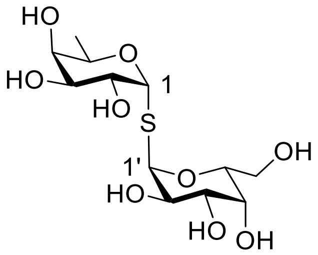

Compound **22** (75 mg, 0.12 mmol) was deacetylated according to the general method D. The crude product was purified by flash column chromatography (CH_2Cl_2:MeOH 7:3) to give compound **28** (35 mg, 88%) as a yellow amorphous solid. R_f = 0.2 (CH_2Cl_2:MeOH 6:4), $[\alpha]_D^{20}$= +150.0 (*c* 1.3 in MeOH) ^{1}H NMR (400 MHz, D_2O) δ 5.55 (d, *J* = 5.6 Hz, 1H, H-1 overlapping with H-1'), 5.55 (d, *J* = 5.6 Hz, 1H, H-1' overlapping with H-1), 4.43 (q, *J* = 6.5 Hz, 1H, H-5), 4.31 (t, *J* = 6.0 Hz, 1H, H-5'*), 4.17 (dd, *J* = 10.3, 5.7 Hz, 1H, H-2 overlapping with H-2'), 4.14 (dd, *J* = 10.6, 5.7 Hz, 1H, H-2' overlapping with H-2), 4.02 (d, *J* = 2.8 Hz, 1H, H-4'*), 3.85 (d, *J* = 2.7 Hz, 1H, H-4), 3.79 – 3.73 (m, 4H, H-6'a,b, H-3, H-3'), 1.23 (d, *J* = 6.6 Hz, 3H, CH_3) ppm. ^{13}C NMR (100 MHz, D_2O) δ 82.6, 82.5 (2C, C-1, C-1'), 72.0 (1C, C-5*). 71.6 (1C, C-4), 70.5, 70.3 (2C, C-3, C-3′), 69.1 (1C, C-4′*), 67.8 (1C, C-5), 67.5, 67.3 (2C, C-2, C-2′), 61.2 (1C, CH_2), 15.4 (1C, CH_3) ppm. *Interchangeable signals. MALDI-ToF-HRMS: *m/z* calcd for $C_{12}H_{22}NaO_9S$ $[M+Na]^+$ 365.0877 found 365.0874

α-D-Galactopyranosyl 1-thio-α-D-galactopyranoside (29)

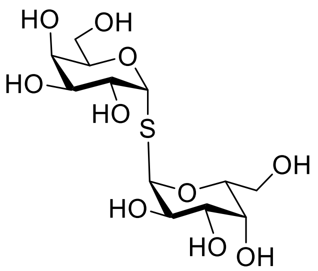

Compound **23** (77 mg, 0.11 mmol) was deprotected according to the general method D. The crude product was purified by flash column chromatography (acetonitrile:water 8:2) to give compound **29** (40 mg, 95%) as colourless foam. R_f = 0.30 (acetonitrile:water 7:3), $[\alpha]_D^{20}$= +314.5 (*c* 0.22 in H_2O). ^{1}H NMR (400 MHz, D_2O) δ 5.59 (d, *J* = 5.7 Hz, 1H, H-1), 4.31 (t, *J* = 6.1 Hz, 1H, H-5), 4.18 (dd, *J* = 10.3, 5.7 Hz, 1H, H-2), 4.00 (d, *J* = 2.9 Hz, 1H, H-6a), 3.81 – 3.72 (m, 3H, H-3, H-4, H-6b) ppm. ^{13}C NMR (100 MHz, D_2O) δ 82.1 (1C, C-1), 72.0. 70.3,

69.1, 67.5 (4C, skeletal carbons), 61.2 (1C, C-6) ppm. MALDI-ToF-HRMS: *m/z* calcd for $C_{12}H_{22}NaO_{10}S$ $[M+Na]^+$ 381.0826 found 381.0830.

α-D-Glucopyranosyl 1-thio-α-D-galactopyranoside (30)

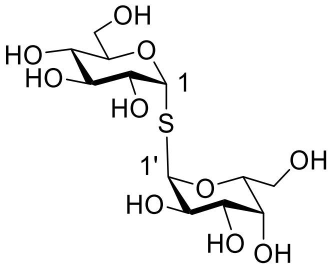

Compound **24** (138 mg, 0.2 mmol) was deacetylated according to the general method D. The crude product was purified by flash column chromatography (acetonitrile:water 8:2) to give compound **30** (68 mg, 96%) as colourless foam. R_f = 0.31 (acetonitrile:water 7:3), $[\alpha]_D^{20}$= +117.0 (*c* 0.27 in DMSO). ^{1}H NMR (400 MHz, DMSO-d_6 + D_2O) δ 5.49 (d, *J* = 5.4 Hz, 1H, H-1), 5.45 (d, *J* = 5.1 Hz, 1H, H-1'), 4.25 – 4.16 (m, 1H), 4.07 (dd, *J* = 10.1, 5.7 Hz, 1H), 4.03 – 3.93 (m, 1H), 3.91 (s, 1H), 3.84 – 3.79 (m, 1H), 3.74 –3.61 (m, 4H), 3.53 (t, *J* = 9.3 Hz, 1H), 3.33 (t, *J* = 9.5 Hz, 1H) ppm. ^{13}C NMR (100 MHz, DMSO-d_6 + D_2O) δ 83.5, 83.1 (1C, C-1, C-1'), 75.3, 74.2, 73.4, 72.1, 71.8, 71.1, 70.5, 68.9 (8C, skeletal carbons), 62.5, 62.1 (2C, C-6, C-6') ppm. MALDI-ToF-HRMS: *m/z* calcd for $C_{12}H_{22}NaO_{10}S$ $[M+Na]^+$ 381.0826 found 381.0800.

3. Agglutination inhibition assay

Both lectin LecA and LecB were dissolved in the buffer (20 mM Tris, 150 mM NaCl, 5 mM $CaCl_2$, pH = 7.5). For LecA, hemagglutination inhibition assay was conducted using red blood cells (RBCs) of group O (purchased from the Transfusion and Tissue Department, The University Hospital Brno, Brno, Czech Republic) stabilized with 3.8% sodium citrate. The RBCs were washed three times by the buffer and diluted to 50% solution, subsequently they were stabilized by 0.01% (w/v) NaN_3 and stored at 4 °C until further use. Before the experiment, RBCs were treated with 0.1% papain for 30 min at 37 °C and then washed three times by the buffer. For inhibition experiments involving lectin LecB, the commercially available baker's yeast (UNIFERM) dissolved in the buffer was used.

Firstly, the cells were mixed with serially diluted lectin solutions in a 1:1 (v/v) ratio to the final concentration of 10 % for the erythrocytes and 2.5 % for the yeast cells. After 10 min incubation at room temperature, mixtures were transferred to a microscope slide and observed using the Levenhuk D2L NG Digital Microscope (Levenhuk). Subsequently, the concentration of the lectin in the condition with the last clearly visible agglutination was chosen for the inhibition tests.

For both lectins, the agglutination inhibition experiments were performed using plastic microtubes according to the procedure described previously [17]. The mixtures of the lectin and serially diluted carbohydrate inhibitors were prepared in a ratio of 1:1 (v/v) in the buffer. Papain-treated, azid-stabilized RBCs group O were added to the LecA-inhibitor mixture in a ratio of 1:1 (v/v). The final concentration was 125 μg/ml for the lectin and 10 % for the RBCs. Similarly, commercially available baker's yeast (UNIFERM) was added to the LecB-inhibitor mixture in a ratio of 1:1 (v/v). The final concentration was 625 μg/ml for the lectin and 2.5 % for the yeast. The mixture was incubated for 10 min at room temperature, then mixed and transferred to a microscope slide for examination. The observation was conducted using the Levenhuk D2L NG Digital Microscope (Levenhuk). Images were obtained with a Levenhuk D2L digital camera (Levenhuk) using the software ToupView for Windows (Levenhuk). As positive control of the inhibition, RBCs or yeast were mixed with the buffer in a ratio of 1:1. As negative control of inhibition, RBCs were mixed with LecA and yeast cells were mixed with LecB to the final concentration of 125 μg/ml and 625 μg/ml, respectively. The minimal inhibitory concentration (MIC) for each tested compound including the standard (Gal for LecA and Fuc for LecB) was determined. Then, the potency of the inhibitor as the ratio between MIC of standard and MIC of ligand was calculated.

4. Isothermal Titration Calorimetry (ITC)

ITC experiments were carried out using an AutoITC200 calorimeter (Malvern, UK) at 25 °C. Freeze-dried lectins LecA and LecB were dissolved in the working buffer (100 mM Tris-HCl, 5 mM $CaCl_2$, pH 7.5) and filtrated by centrifugation (3 min, 8 000 g) in microtubes with 0.22 μm filter (*Avantor*). Carbohydrate ligands (Fuc, Gal, and compounds **27** and **14**) were dissolved in the same buffer. Both lectins (300 μM LecA and 40 μM LecB) in the cell was titrated by the successive addition (20 injections of 2.0 μL) of the ligand in the syringe (4 mM carbohydrates for LecA, 0.4 mM carbohydrates for LecB). Experiments were performed at stirring 500 rpm with a reference power of 5 μcal.s^{-1}. Spacing between individual injections was 180 s and 320 s for LecA and LecB, respectively. Only in the case of LecB and Fuc, the

spacing was 240 s. For each carbohydrate, the blank measurement was performed: the titration of a given ligand into the cell with buffer. Blank measurements were subtracted from collected data. The data were evaluated in software MicroCal PEAQ-ITC Analysis Software (Malvern, UK) by using the global fit method. Integrated heat effects were analyzed by nonlinear regression. The fitted data yielded the equilibrium dissociation constant ($K_D = 1/K_a$).

5. Dynamic light scattering (DLS)

DLS measurements were conducted using a SpectroLight 600 (Xtal Concepts) in plate-based format. The LecA and LecB solutions were dissolved in the working buffer (100 mM Tris-HCl, 5 mM $CaCl_2$, pH 7.5) and filtrated by centrifugation (5 min, 8 000 g) in microtubes with 0.22 μm pore size filter (*Avantor*). Both lectins were mixed with serially diluted carbohydrate compound **14** in a ratio of 1:1 (v/v) in the working buffer. The final concentration of both lectins was 400 μM. The highest concentration of the compound **14** was 25 mM. Additionally, a mixture of both lectins in a molar ratio 1:1 (200 μM for each lectin) was mixed with serially diluted carbohydrate compound **14** in a ratio of 1:1 (v/v) in the working buffer. As control, lectin LecA, LecB, or mixture of the lectins were diluted by the working buffer in a ratio of 1:1 (v/v) without the presence of the inhibitor.

The experiment was carried out at 20 °C. Scans were collected every 90 minutes to a total number of eight scans per condition. The refractive index and viscosity values were taken for the water as provided by the software (SpectroLight 600).

6. Analytical ultracentrifugation (AUC)

AUC method of sedimentation velocity was used to examine possible changes in the oligomeric state of a mixture of lectins LecA and LecB in the presence of the compound **14**. As controls, LecA, LecB, and a mixture of both lectins were tested in the concentration of 20 μM. Subsequently, the oligomeric states of a mixture containing both lectins (20 μM) and five distinct inhibitor concentrations (0.4 μM, 4 μM, 20 μM, 100 μM and 1000 μM) were assessed.

7. X-ray crystallography

The crystallization of lectin complexes with α,β-thiodisaccharides **14** was optimized based on previously described conditions [18,19], utilizing the sitting drop vapor diffusion technique in 24-well plates. LecA, at a concentration of 8.5 mg/mL in a solution containing 2.0 mM compound 14 and 0.1 mM $CaCl_2$, and LecB, at a concentration of 7.6 mg/mL in a solution

containing 2.0 mM compound **14** and 1.0 mM $CaCl_2$, were incubated for 1 hour prior to crystallization. Drops were set up in ratios of 2:1, 1:1, and 1:2 for protein to precipitant solution and stored at 17 °C. Crystals of lectin complexes were mounted using a litho-loop. LecA-**14** crystals were directly flash-frozen in liquid nitrogen, while LecB-**14** crystals were cryoprotected in a 1:1 solution of 80% PEG400 and mother liquor before vitrification. The most promising diffracting crystals for the LecA-**14** complex were obtained under condition 2:1 ratio of protein to precipitant solution (11.5% PEG 8000, 11.5% PEG 1000, 200 mM $MgCl_2$, and 100 mM Tris-HCl pH 8.5), and for the LecB-**14** complex, under condition 1:2 ratio of protein to precipitant solution (30% PEG 8000, 150 mM $(NH_4)_2SO_4$, and 100 mM Tris-HCl pH 8.5).

Diffraction data were collected at the synchrotron PETRAIII beamline P13 using an EIGER16M detector [20] using mxCube v2 [21]. Data processing was performed using autoproc [22] and merged using Scala [23]. The coordinates of PDB models 4LJH (LecA) and 5A3O (LecB) served as initial models for molecular replacement using PHASER [24]. Structural refinement was conducted in REFMAC5 [25], supplemented with manual model building and correction in COOT [26]. Cross-validation analysis was performed using five percent of the observations. Waters were added automatically and verified manually. Ligands were assigned based on Fo – Fc electron density maps with peak heights exceeding 3σ. Topology files for thiosaccharide ligands were generated using the JLigand [27] program from the CCP4 suite [28]. The final models were validated in PDBe validation server (https://validate-rcsb-1.wwpdb.org/) and deposited with PDB IDs: 9G3R, 9G3S. The refinement statistics are given in **Table S1**.

Results and discussion

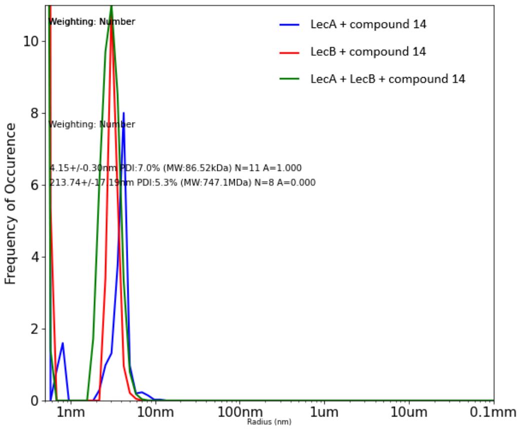

Figure S1: Dynamic light scattering (DLS) analysis showing the particle size distribution based on frequency of occurrence in the working buffer (100 mM Tris-HCl, 5 mM $CaCl_2$, pH 7.5). The final concentration is 400 µM and 3 125 µM for the protein(s) and the compound **14**, respectively. LecA-compound **14** sample (blue curve), LecB-compound **14** sample (red curve), and mixture of both lectins with the compound **14** (green curve) are shown.

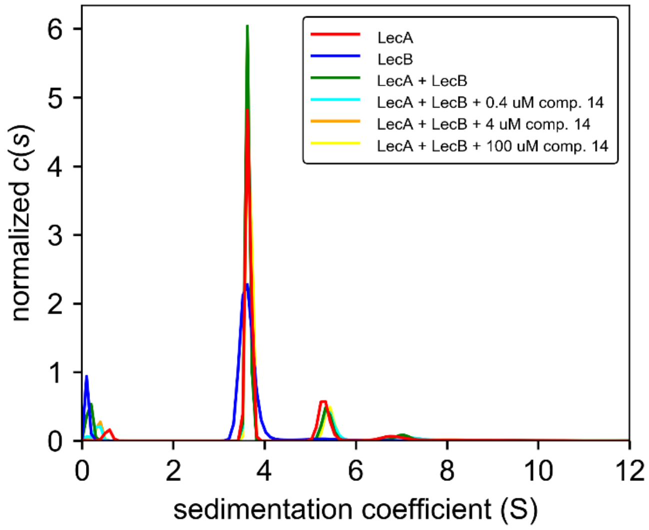

Figure S2: Analytical ultracentrifugation of LecA, LecB and mixture of both lectins with and without the presence of the compound **14**. Absorbance (280 nm) sedimentation velocity data obtained from continuous c(s) analysis. The corresponding c(s) distribution predominantly showing peaks with the sedimentation coefficient of 3.7 S and 3.6 S corresponding to the tetrameric form of LecA and LecB, respectively. The figure was created in GUSSI [29].

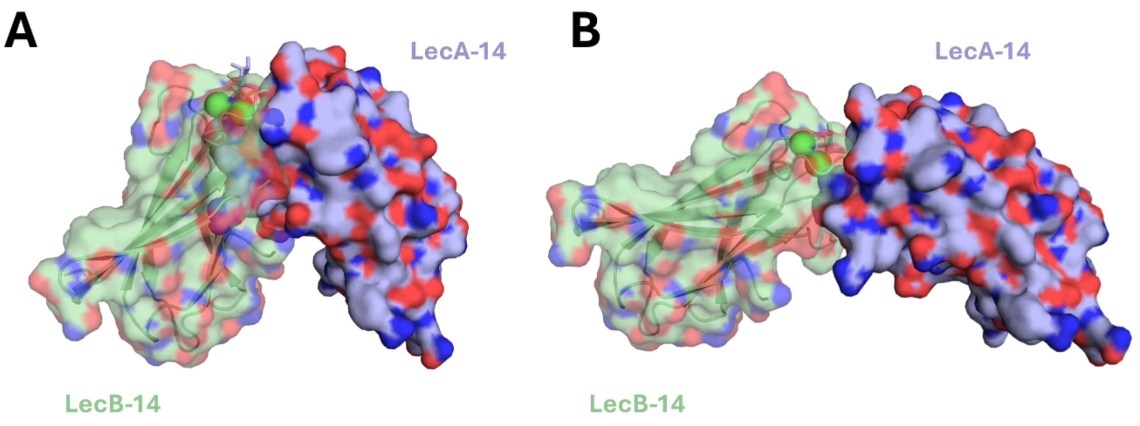

Figure S3: Visualization of a potential simultaneous binding complex of LecA-**14** and LecB-**14** by structural superposition through D-galactose (A) or L-fucose (B) moieties.

Table S1: Data collection and refinement statistics.

	LecA-14<br>PDB:9G3R	LecB-14<br>PDB:9G3S
Beam line	PETRAIII P13	PETRAIII P13
WaveLength (Å)	0.9760	0.9760
SpaceGroup	$P12_11$	$P2_12_12_1$
Unit-cell parameters<br>a/b/c (Å)<br>α/β/γ (°)	50.90/99.82/86.84<br>90.00/90.00/90.00	74.49/96.95/128.68<br>90.00/90.00/90.00
Resolution range (Å)<br>(Outershell)	49.91 – 1.70<br>1.79 – 1.70	48.69 – 1.85<br>1.95 – 1.85
Total reflections measured	544654<br>82912	1091077<br>161398
Unique reflections	93512<br>13554	80145<br>11547
I/Sig (I)	8.5<br>2.7	12.0<br>2.2
CC1/2 (%)	99.2<br>83.4	99.9<br>75.3
Completeness (%)	98.3<br>98.3	99.9<br>99.9
R merge	0.129<br>0.515	0.202<br>1.847
Multiplicity	6.7<br>7.0	13.6<br>14.0
R_{work} (%)	20.6	21.9
R_{free} (%)	23.4	22.4
RMSD bond lengths	0.0081	0.0075
RMSD bond angles	1.5640	1.4774
No. waters	353	336
No. of non-H atoms	7758	7177
Ramachandran outliers	0	0

NMR spectra:

^{1}H and ^{13}C NMR spectra of compound **10**

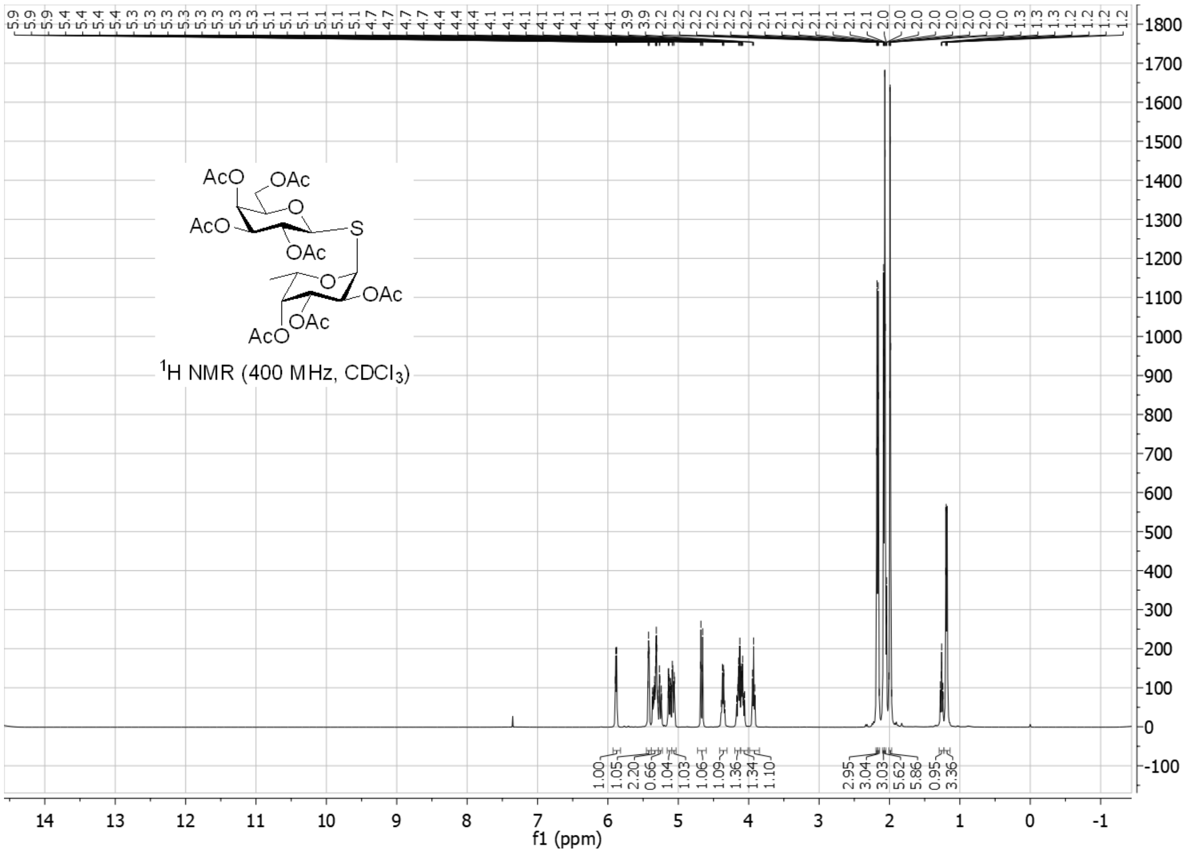

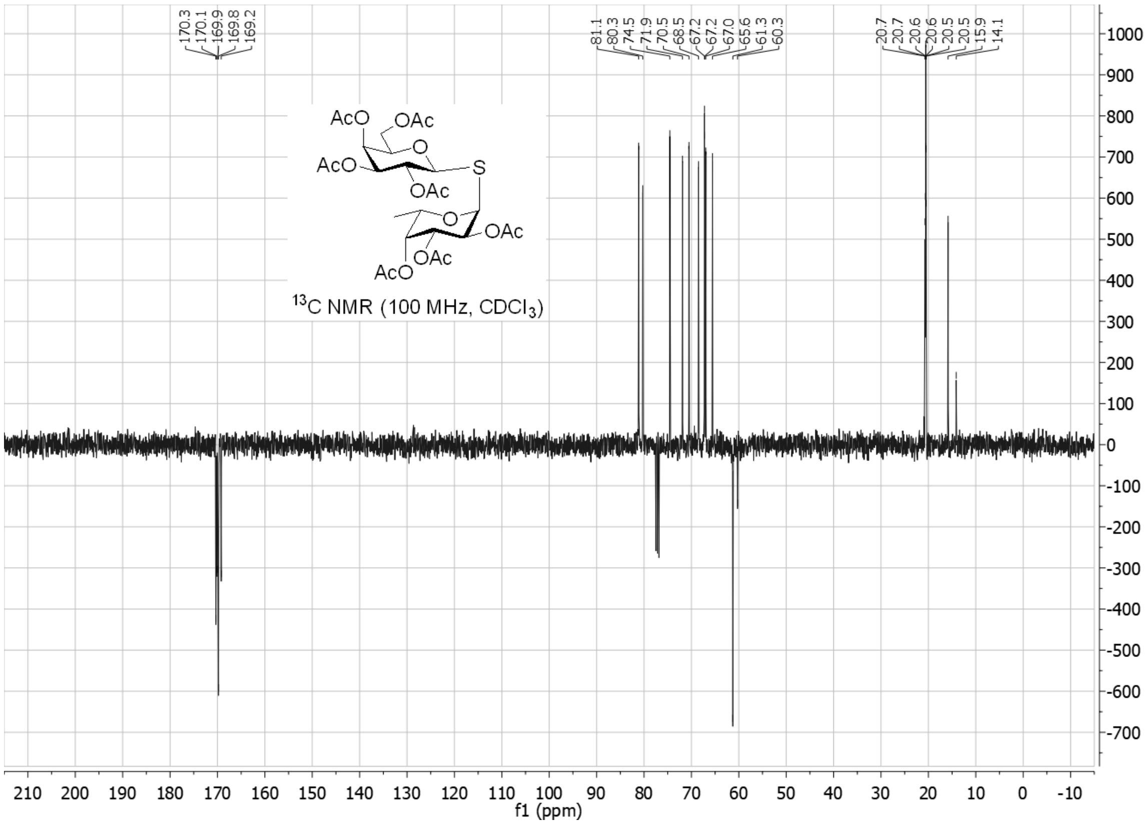

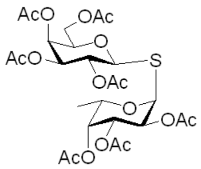

^{1}H-^{1}H COSY NMR (400 MHz, $CDCl_3$)

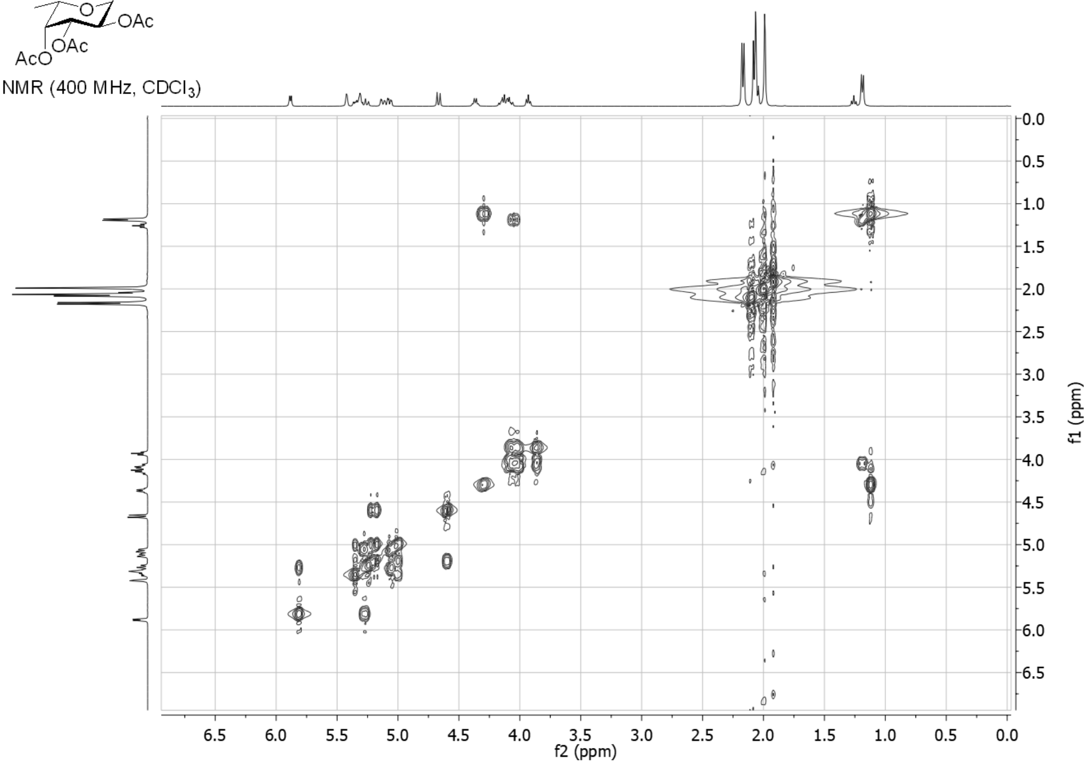

^{1}H and ^{13}C NMR spectra of compound **11**

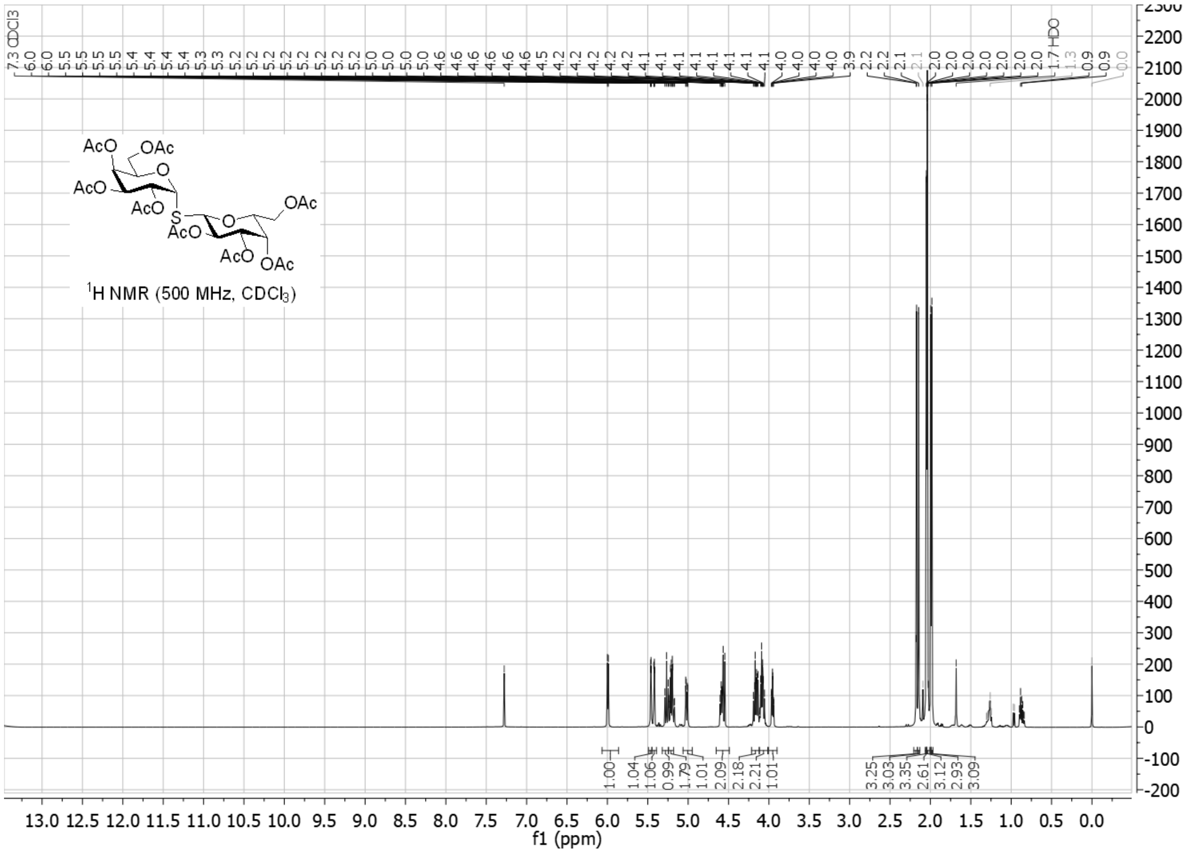

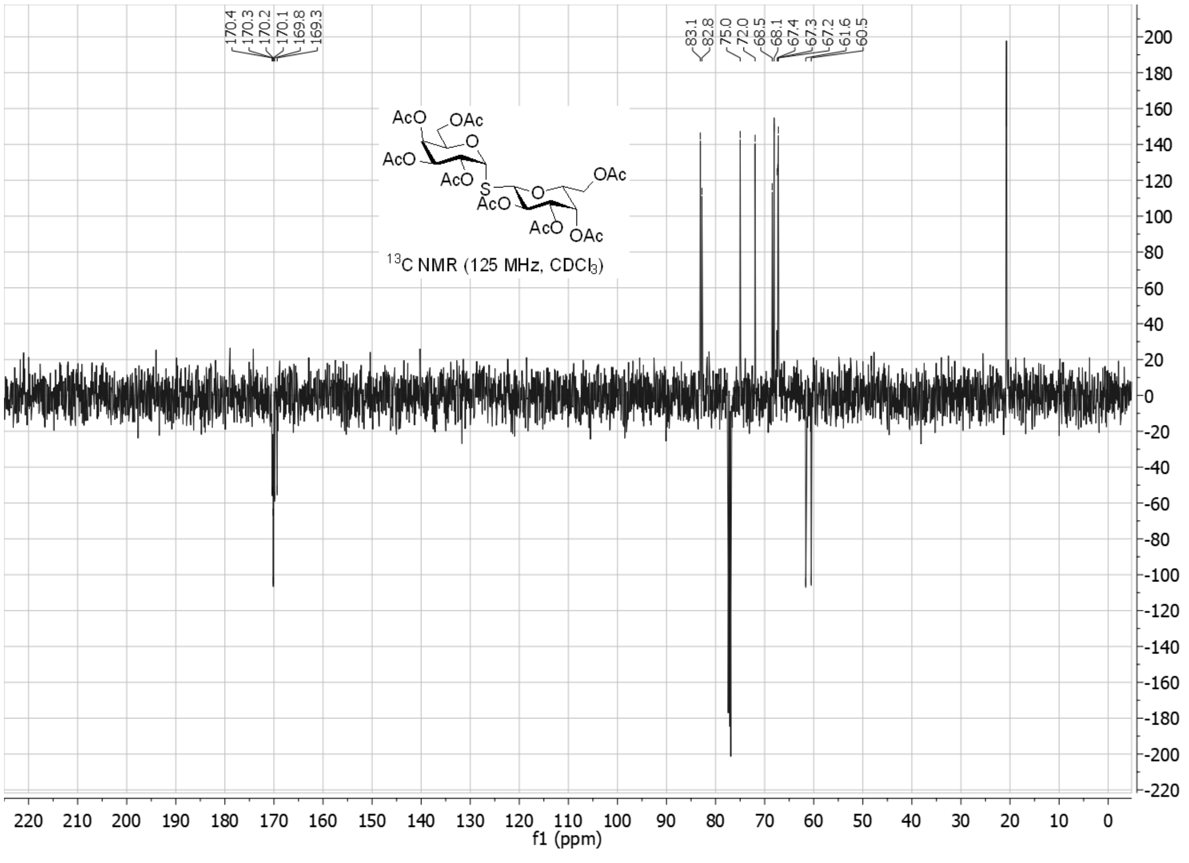

^{1}H and ^{13}C NMR spectra of compound **12**

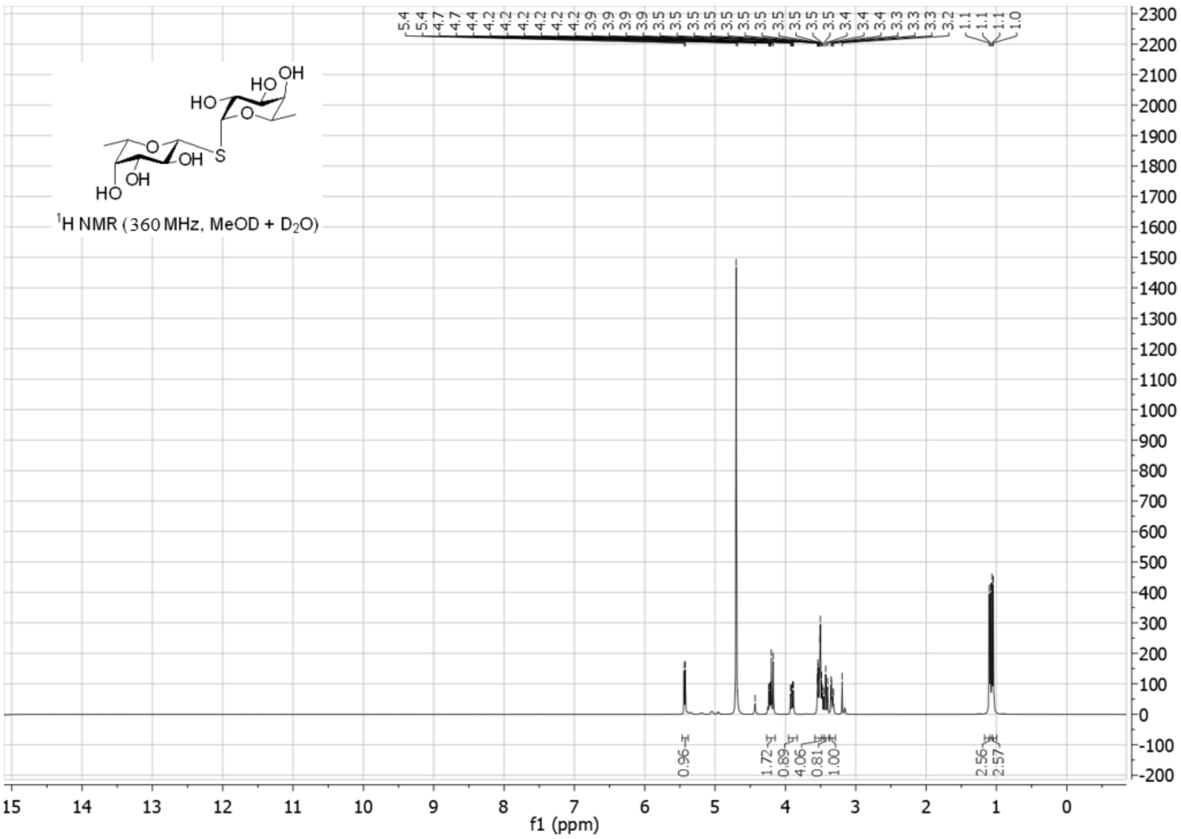

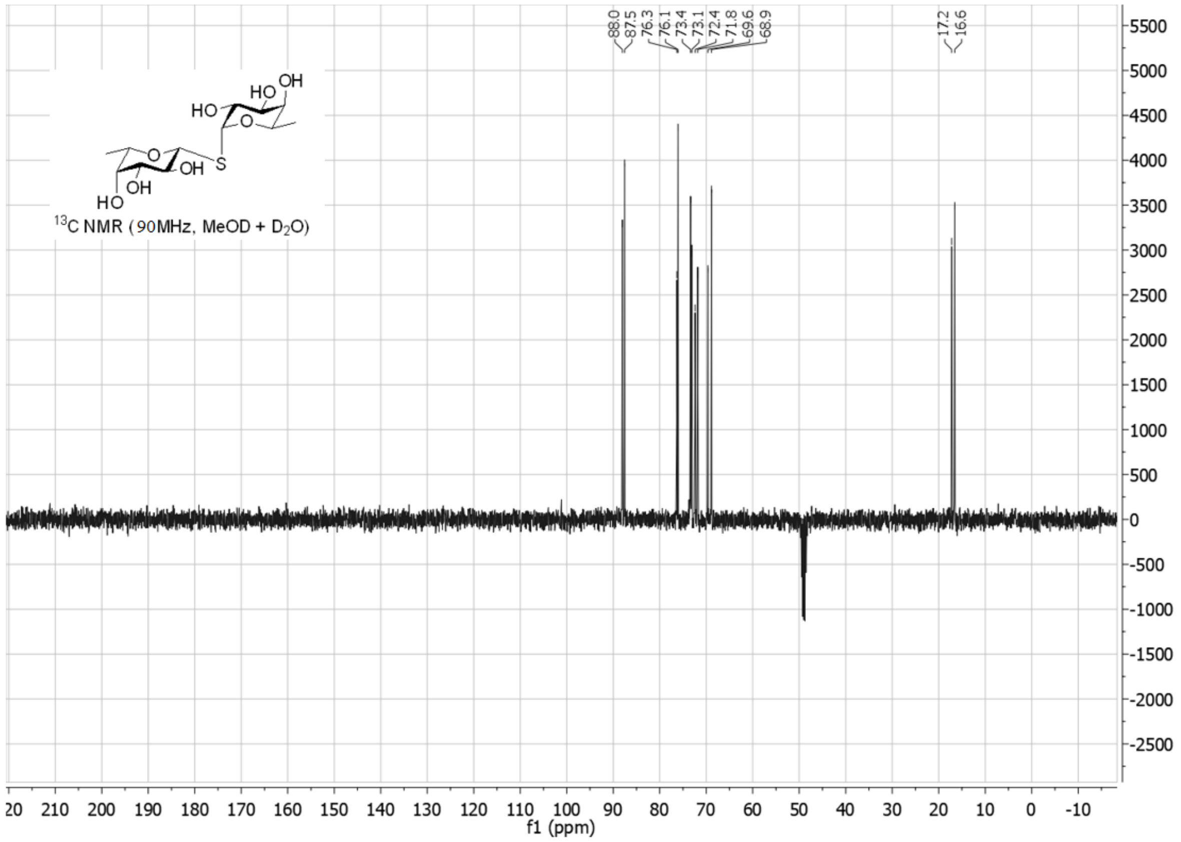

^{1}H and ^{13}C NMR spectra of compound **13**

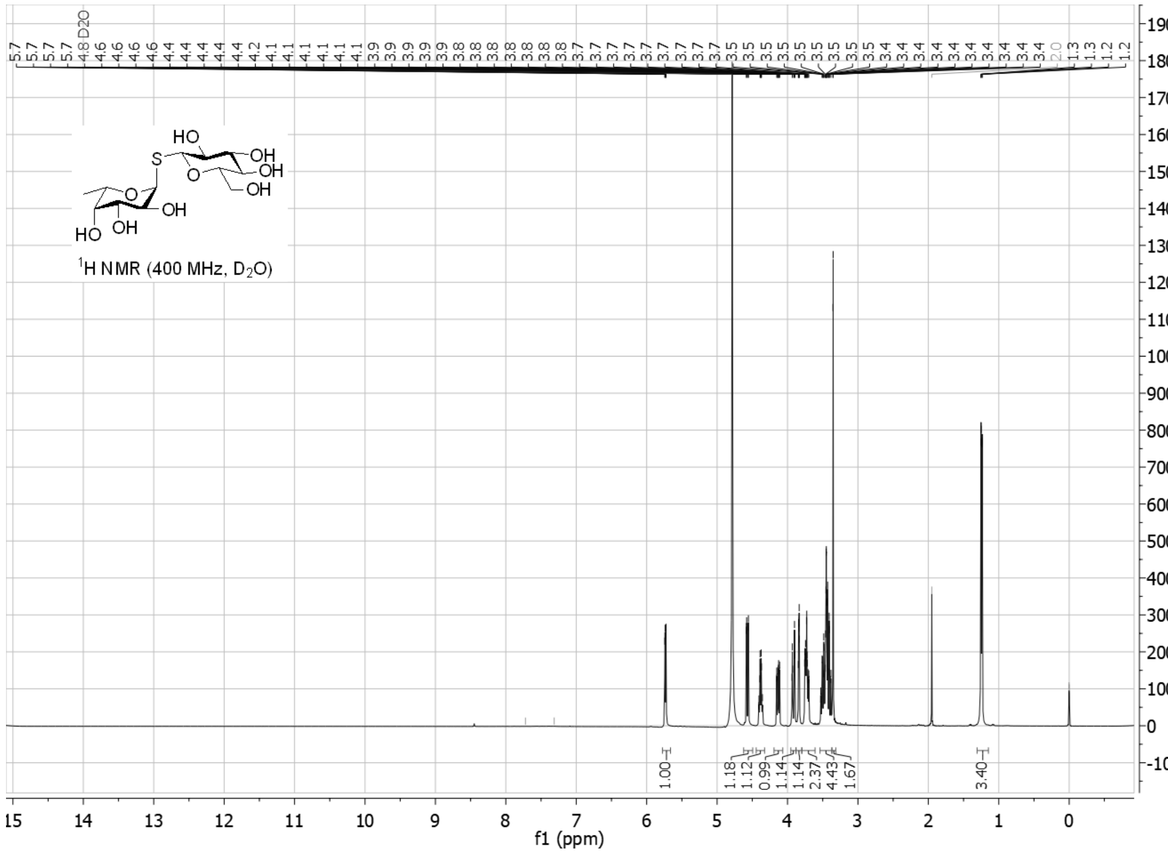

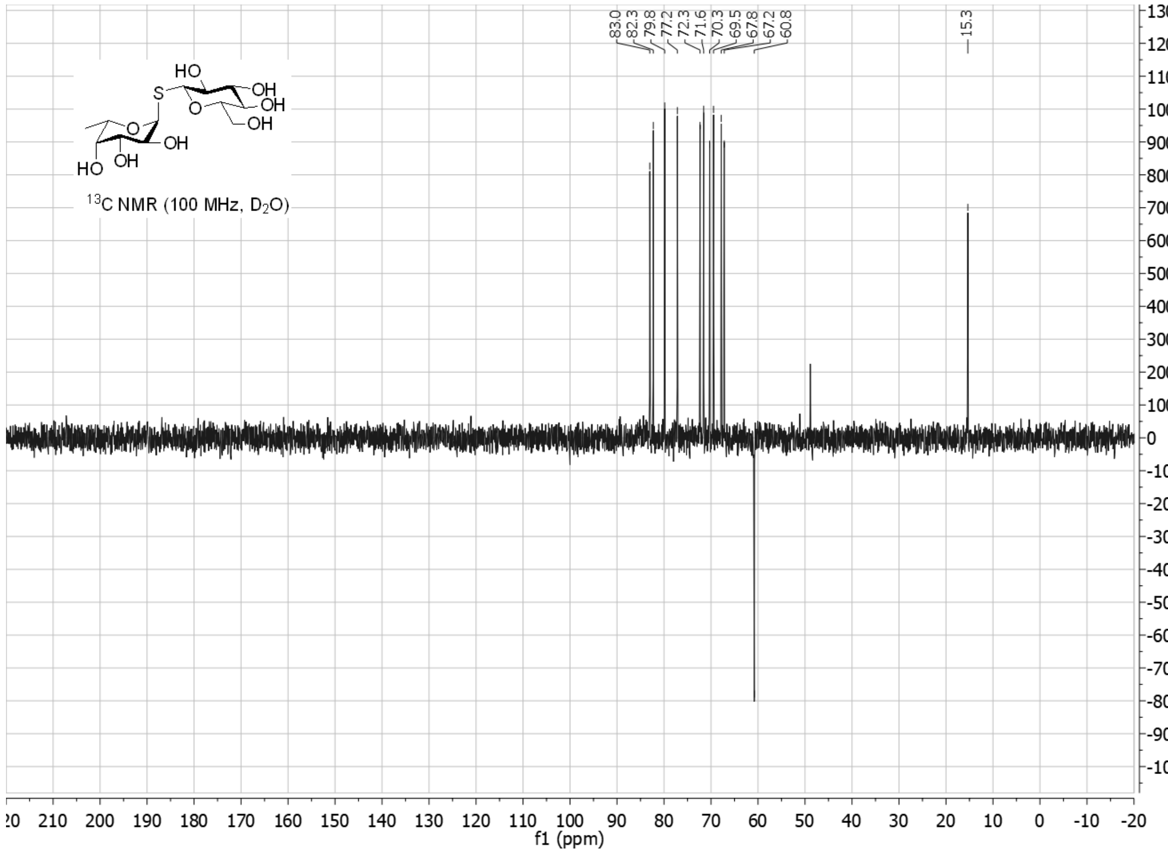

^{1}H and ^{13}C NMR spectra of compound **14**

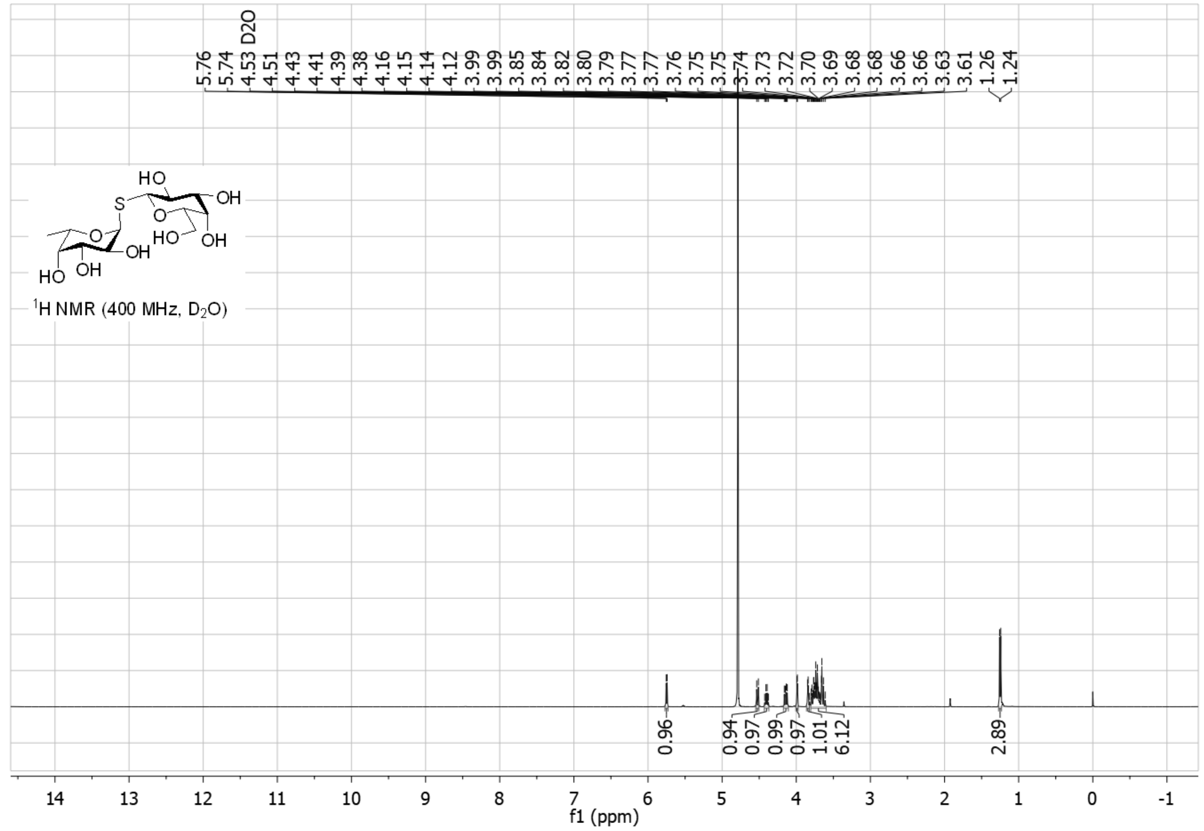

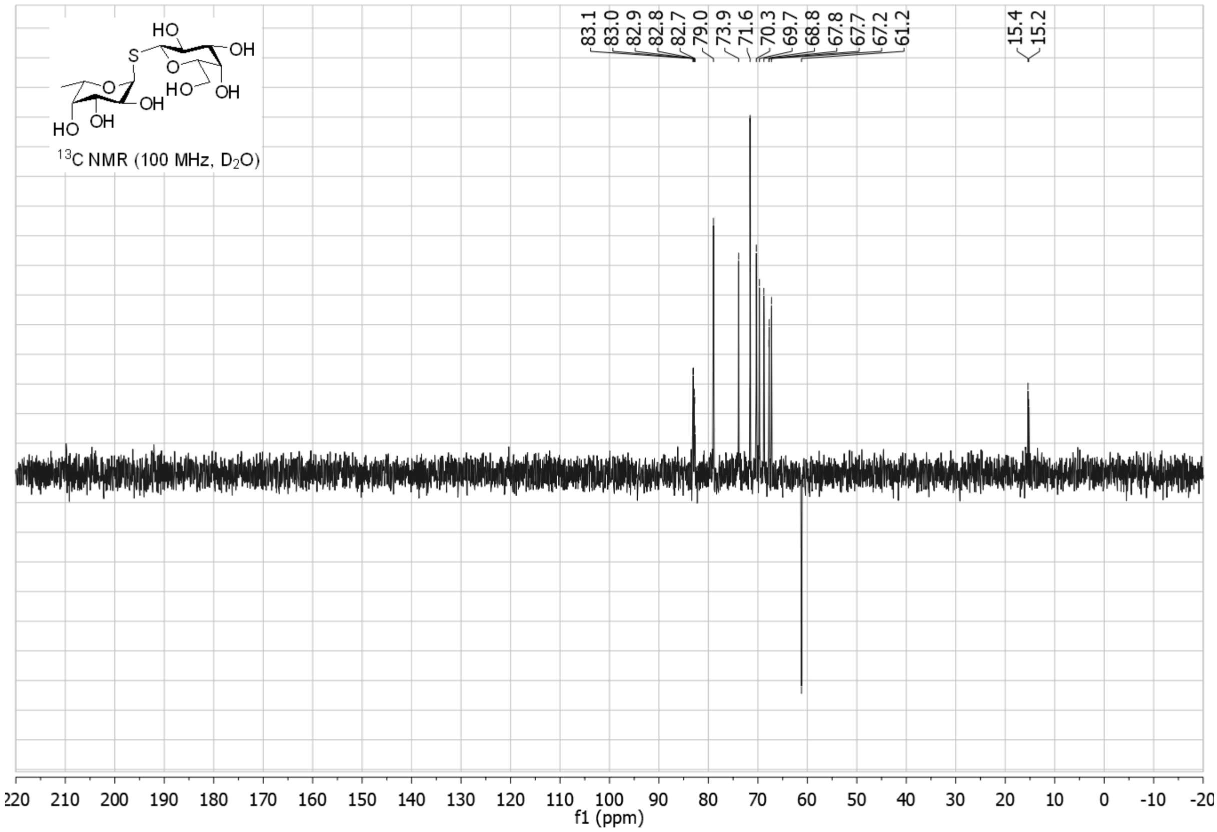

^{1}H and ^{13}C and ^{1}H-^{1}H COSY NMR spectra of compound **15**

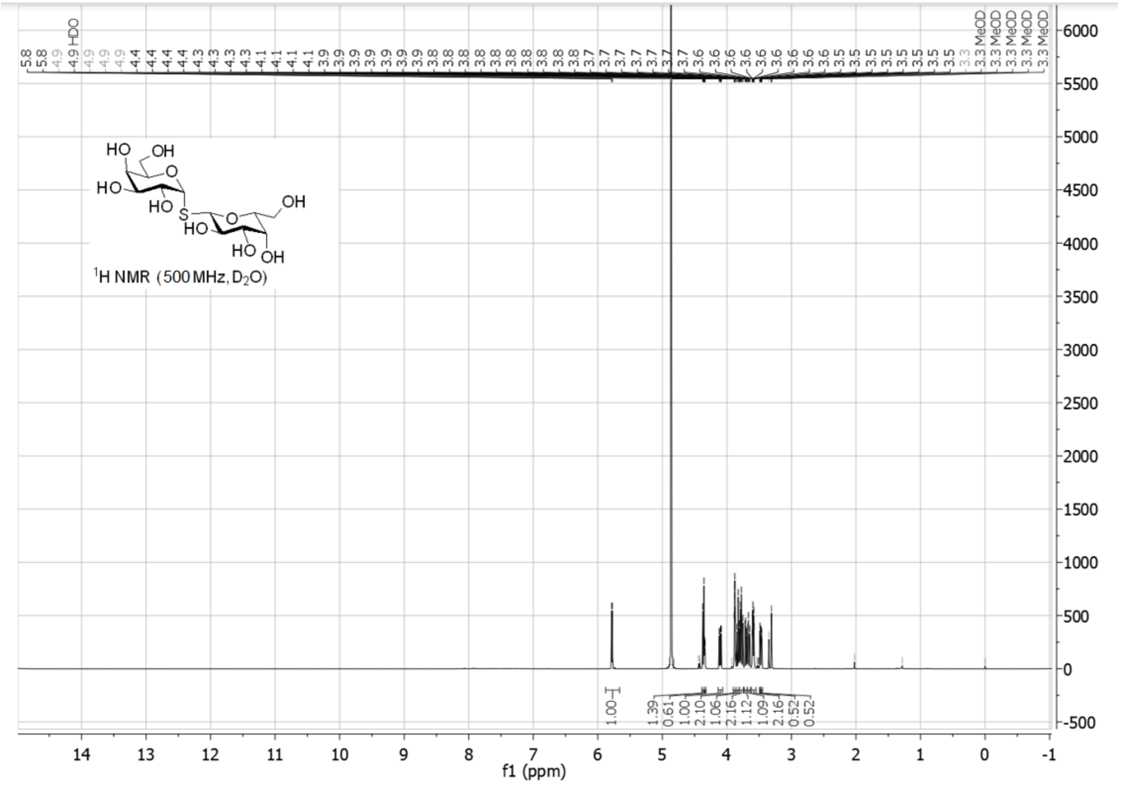

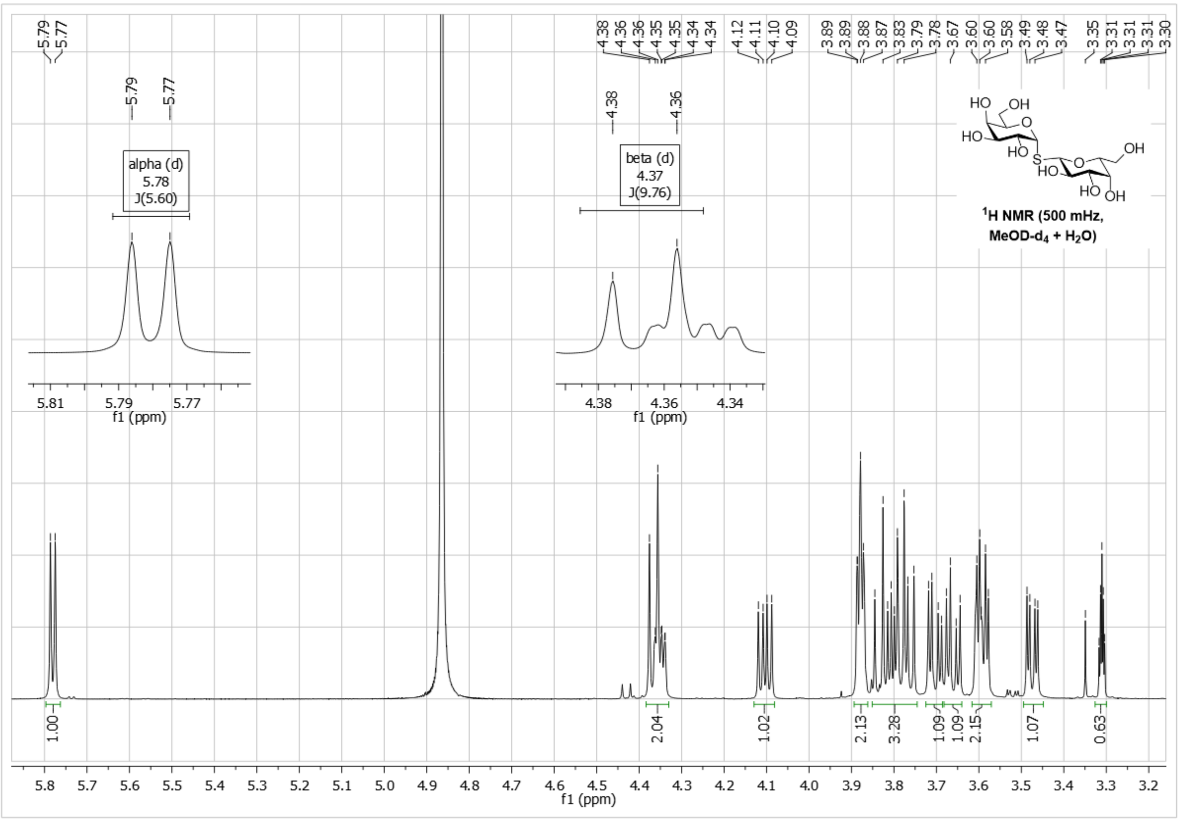

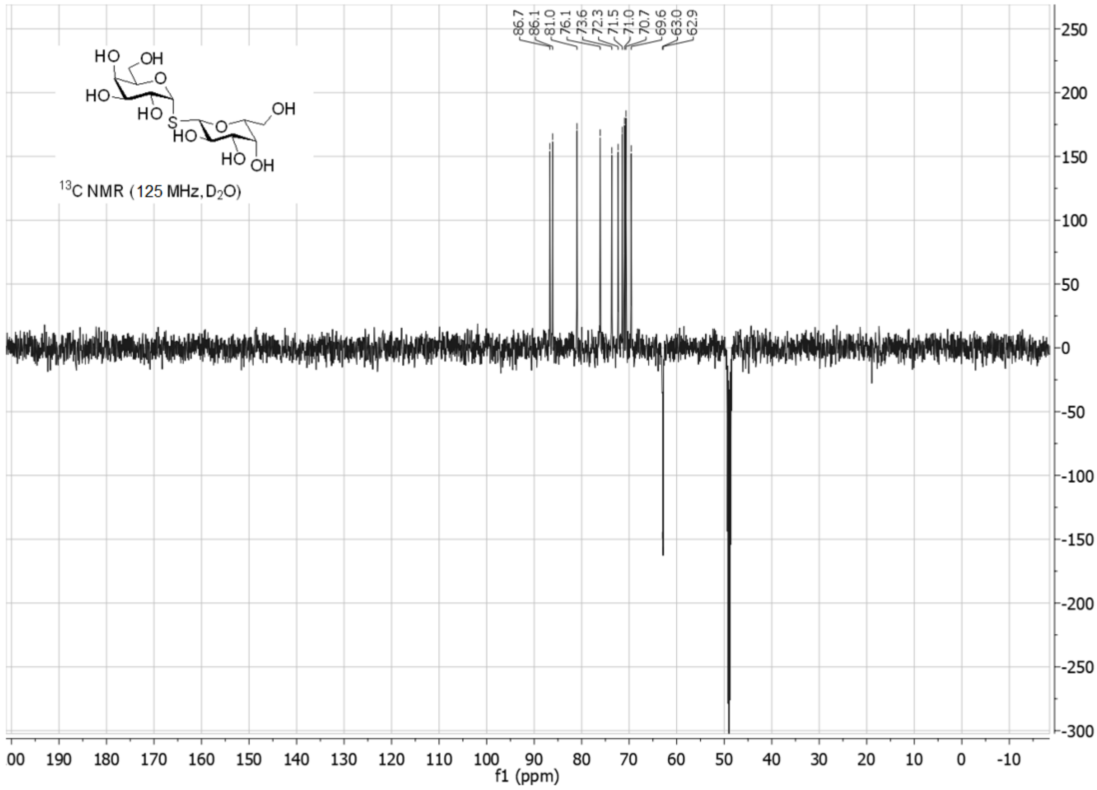
86.7
86.1
81.0
76.1
73.6
72.3
71.5
71.0
70.7
69.6
63.0
62.9
^{13}C NMR (125 MHz, D$_2$O)
f1 (ppm)

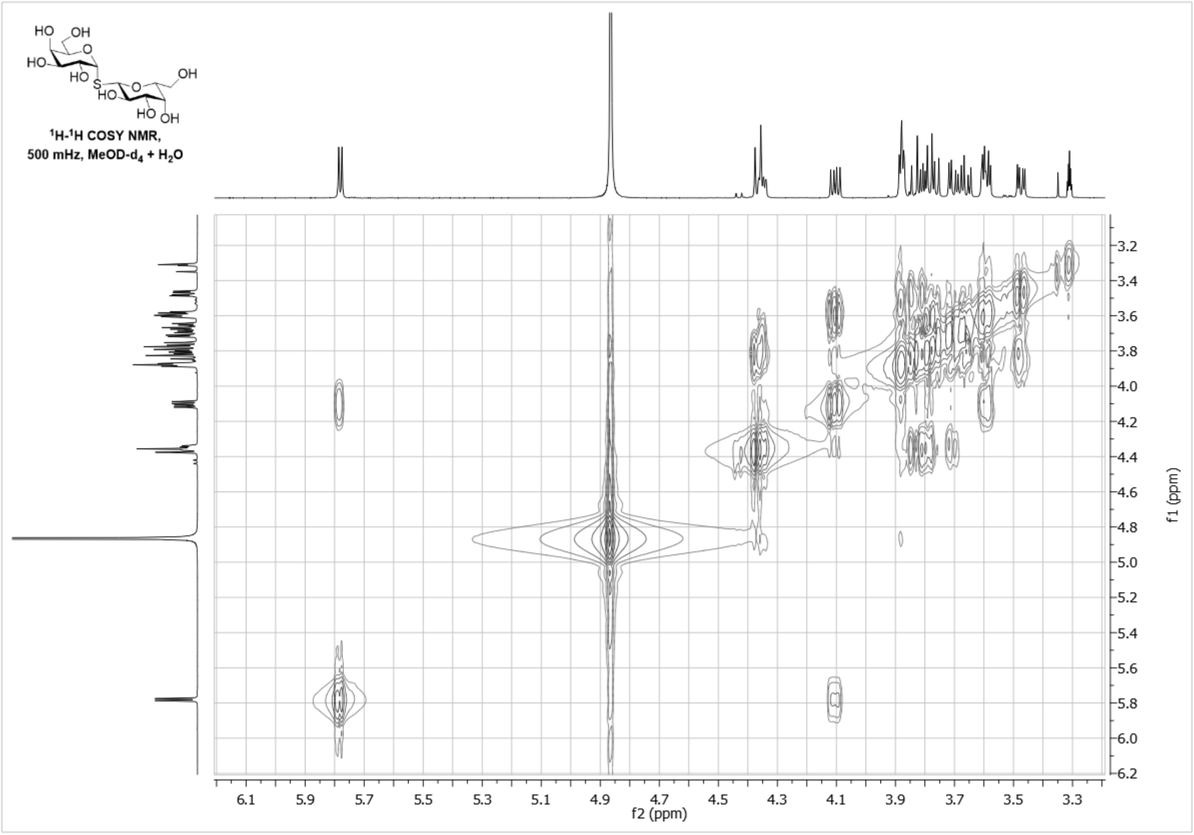
^{1}H-^{1}H COSY NMR,
500 mHz, MeOD-d$_4$ + H$_2$O
f2 (ppm)
f1 (ppm)

^1H and ^{13}C NMR spectra of compound **16-SAc**

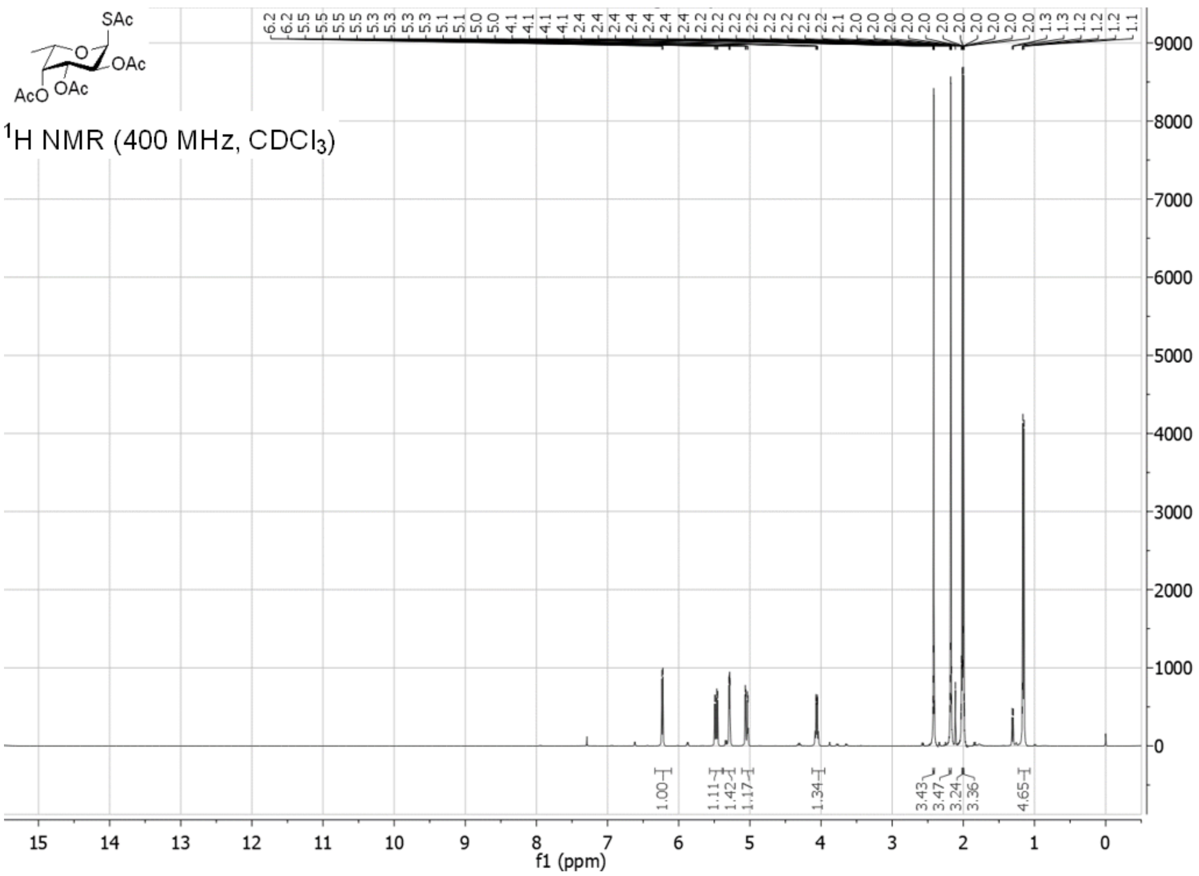

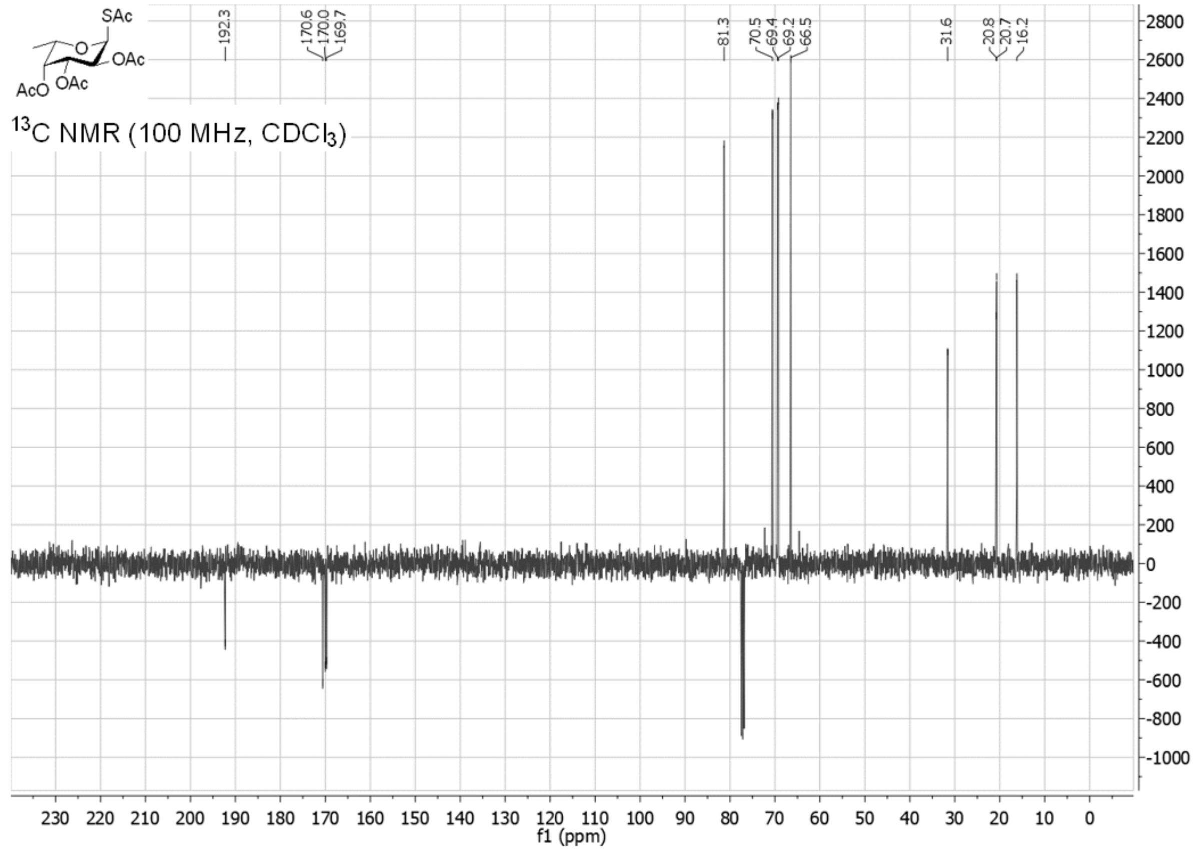

^{1}H and ^{13}C NMR spectra of compound **17-SAc**

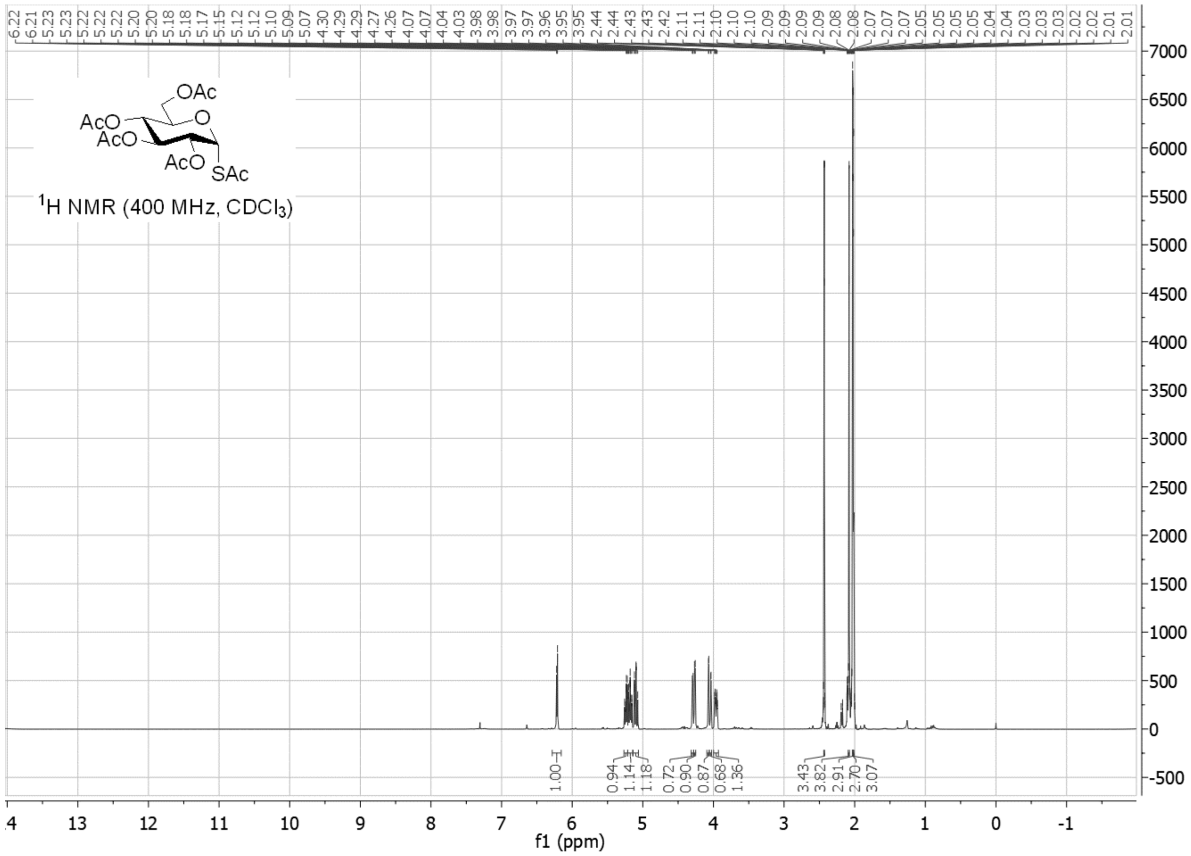

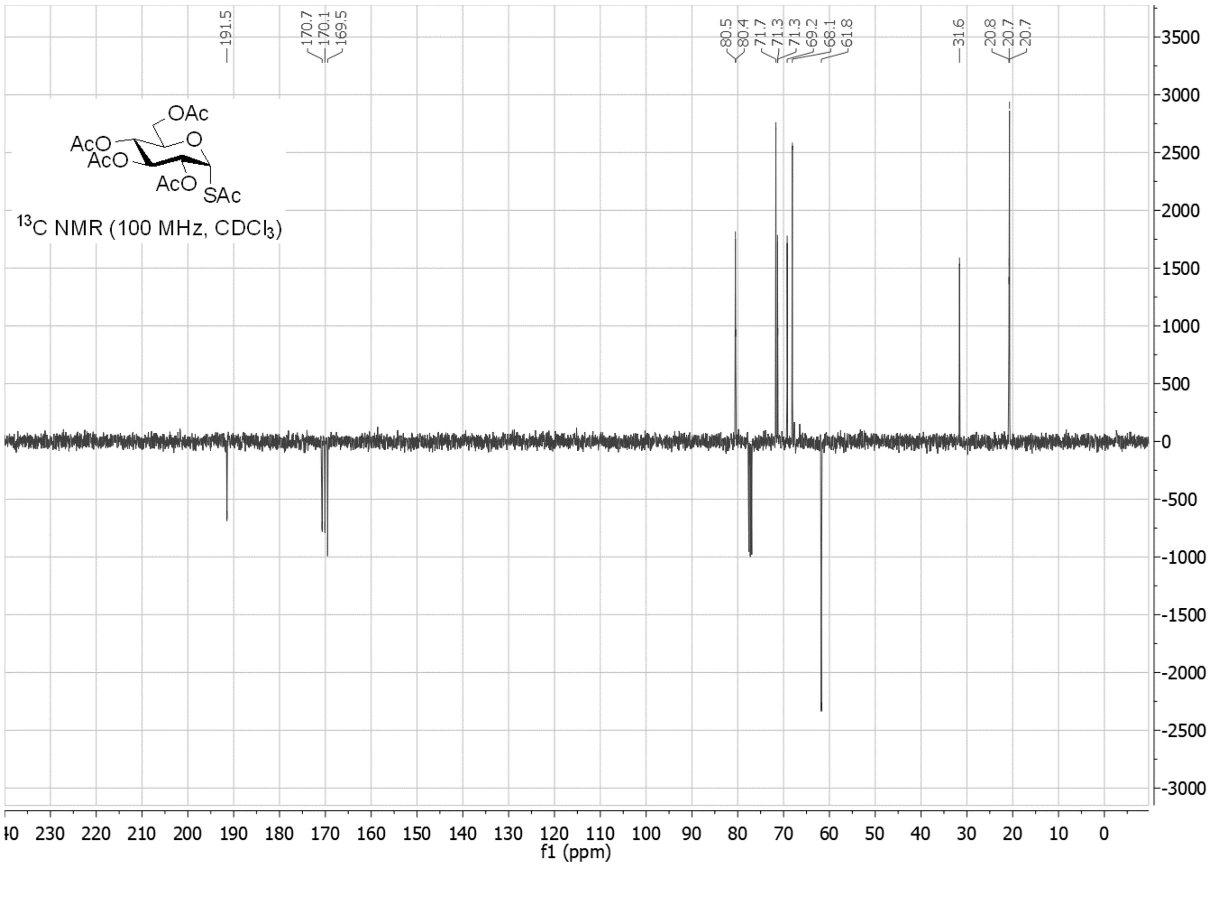

^{1}H and ^{13}C NMR spectra of compound **18-SAc**

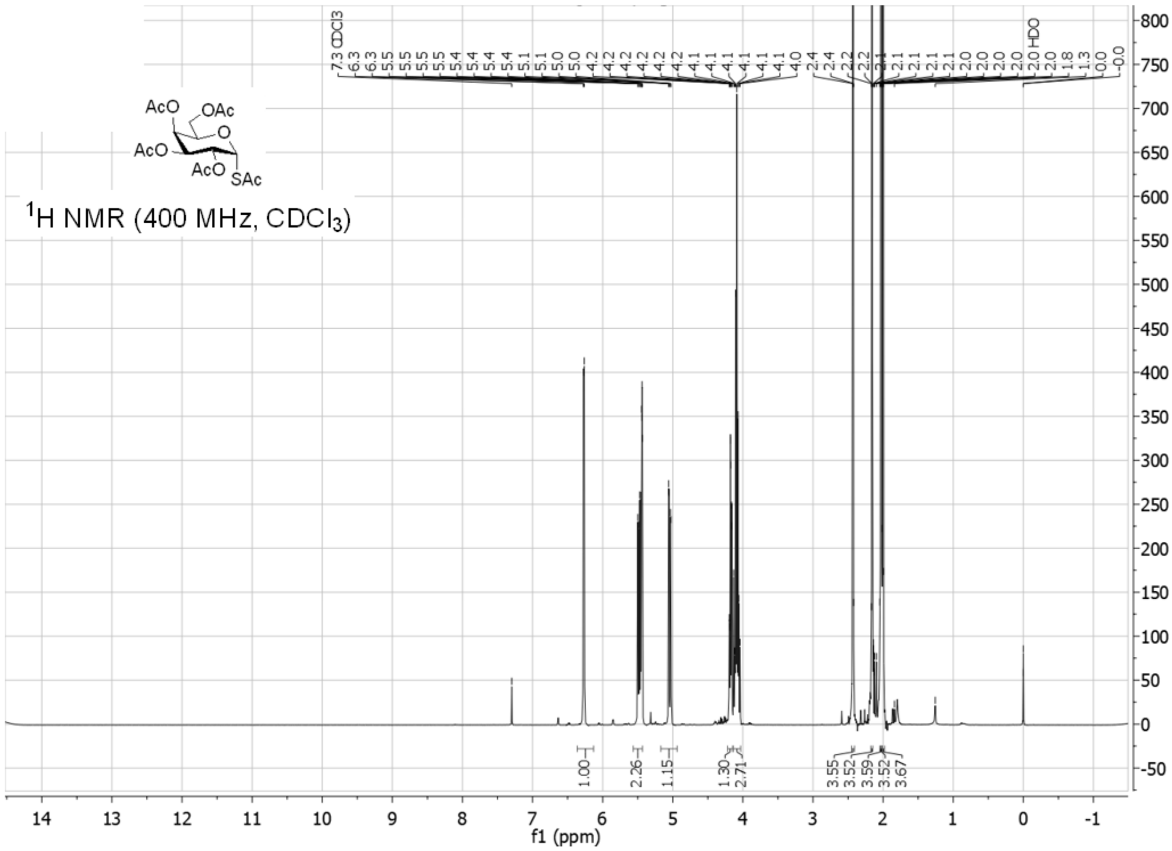

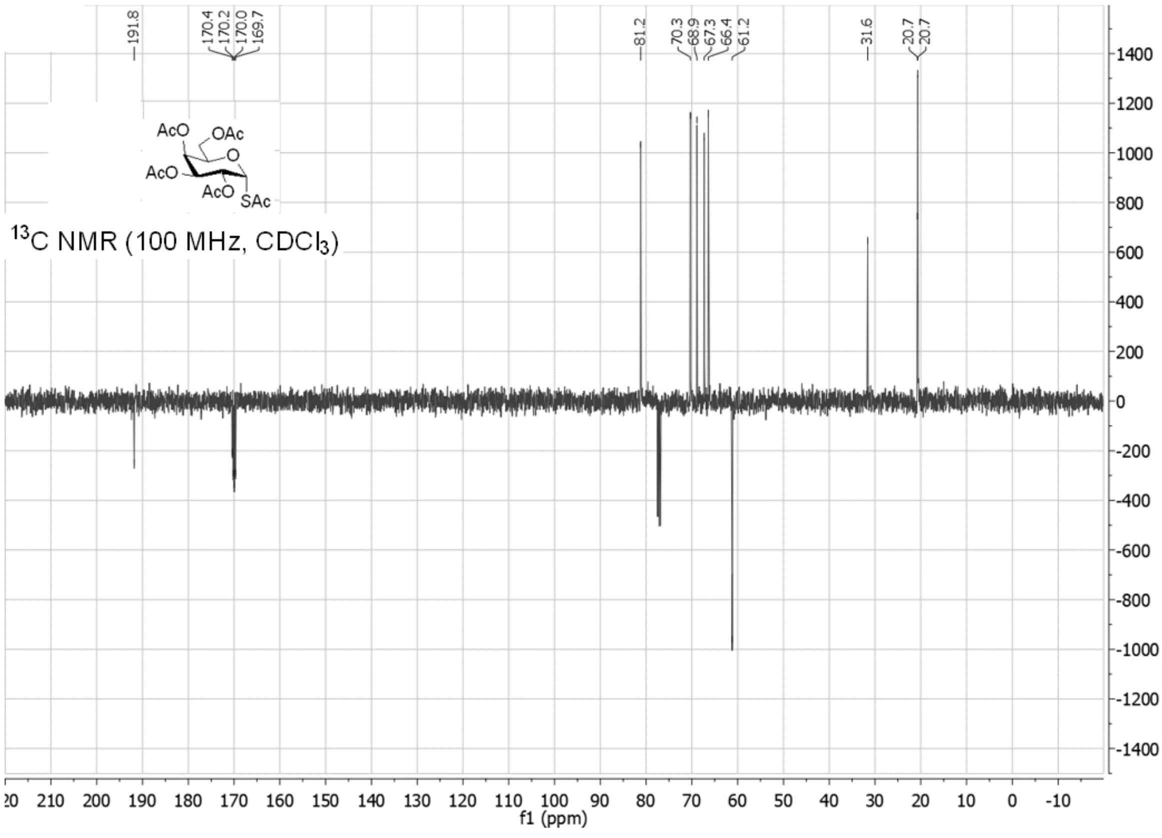

^{1}H and ^{13}C NMR spectra of compound **16**

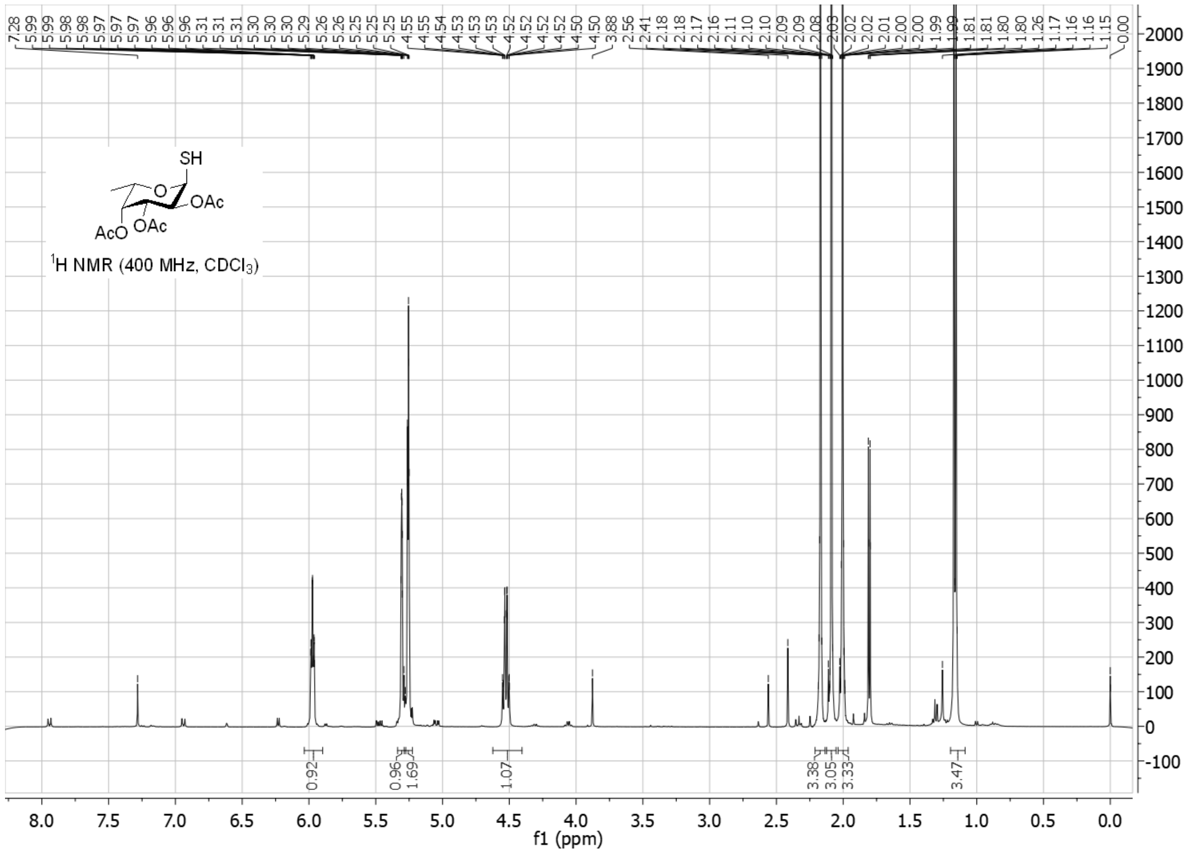

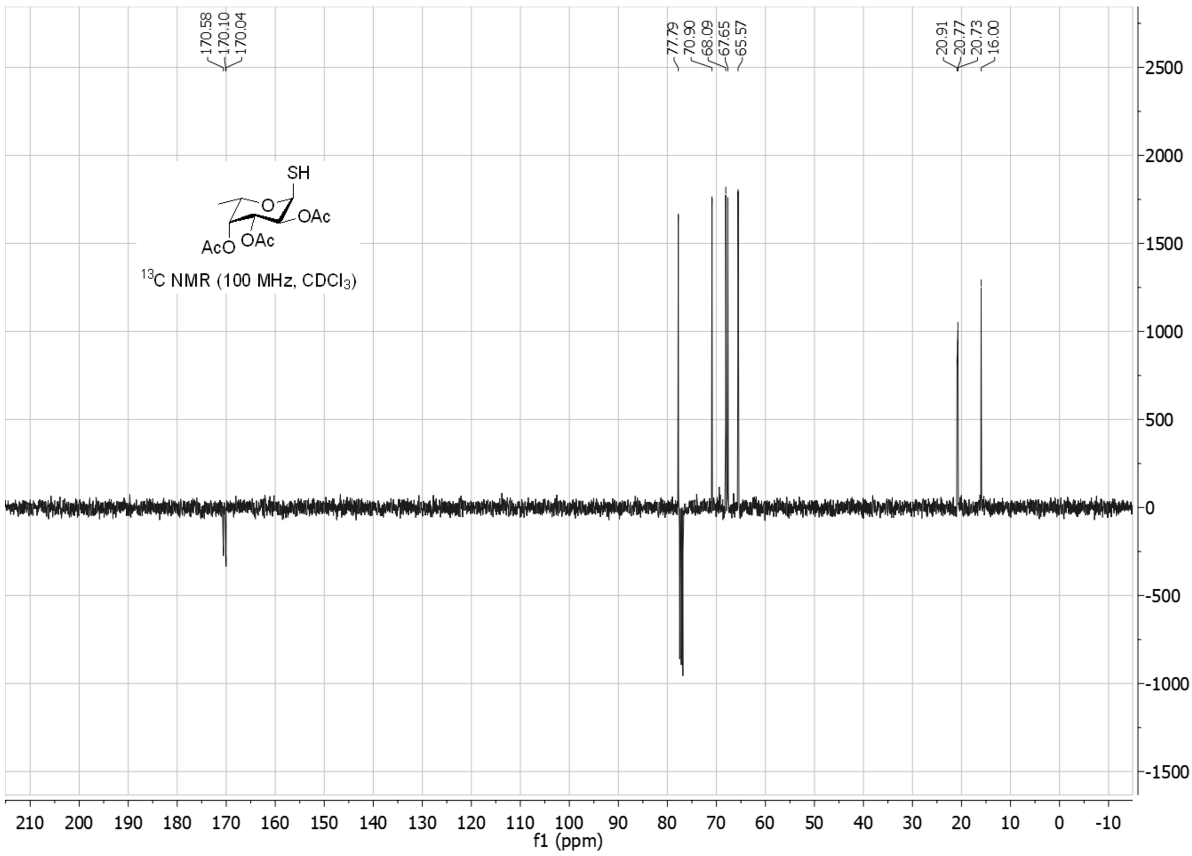

^{1}H and ^{13}C NMR spectra of compound **17**

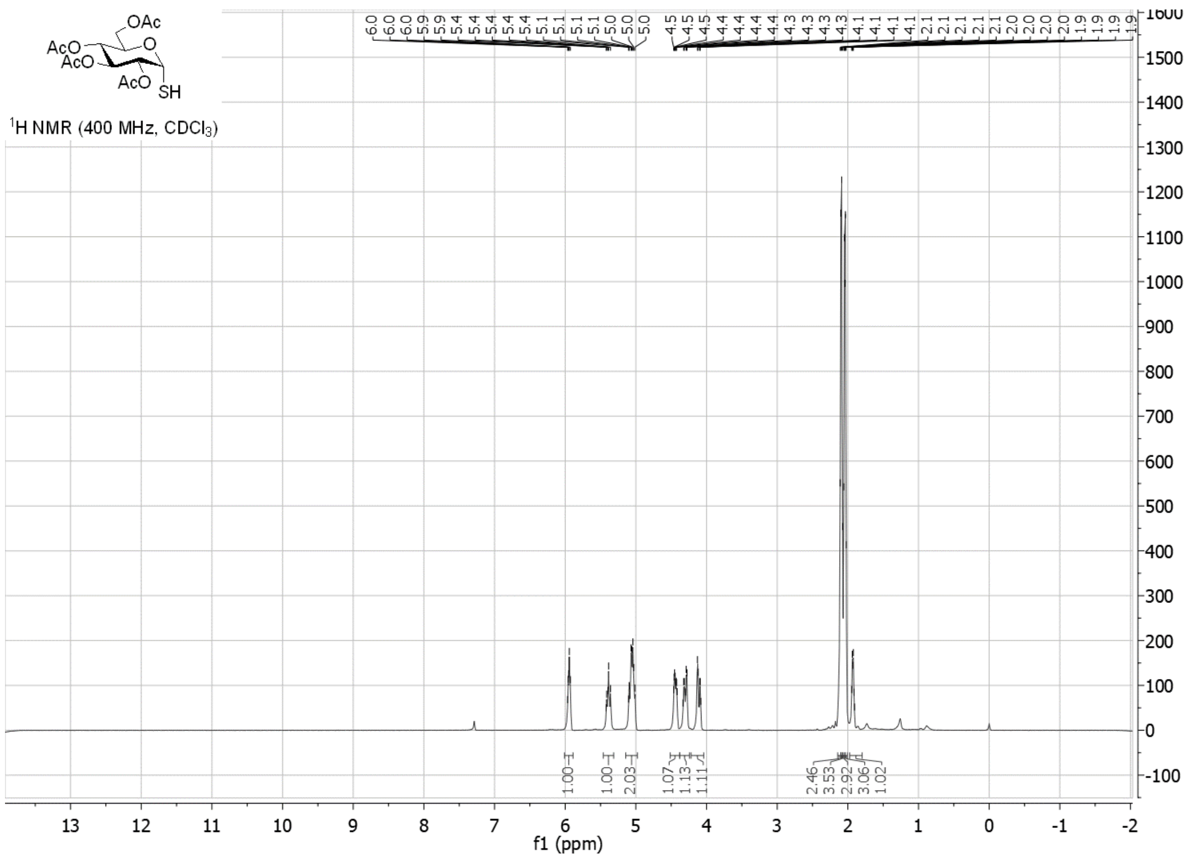

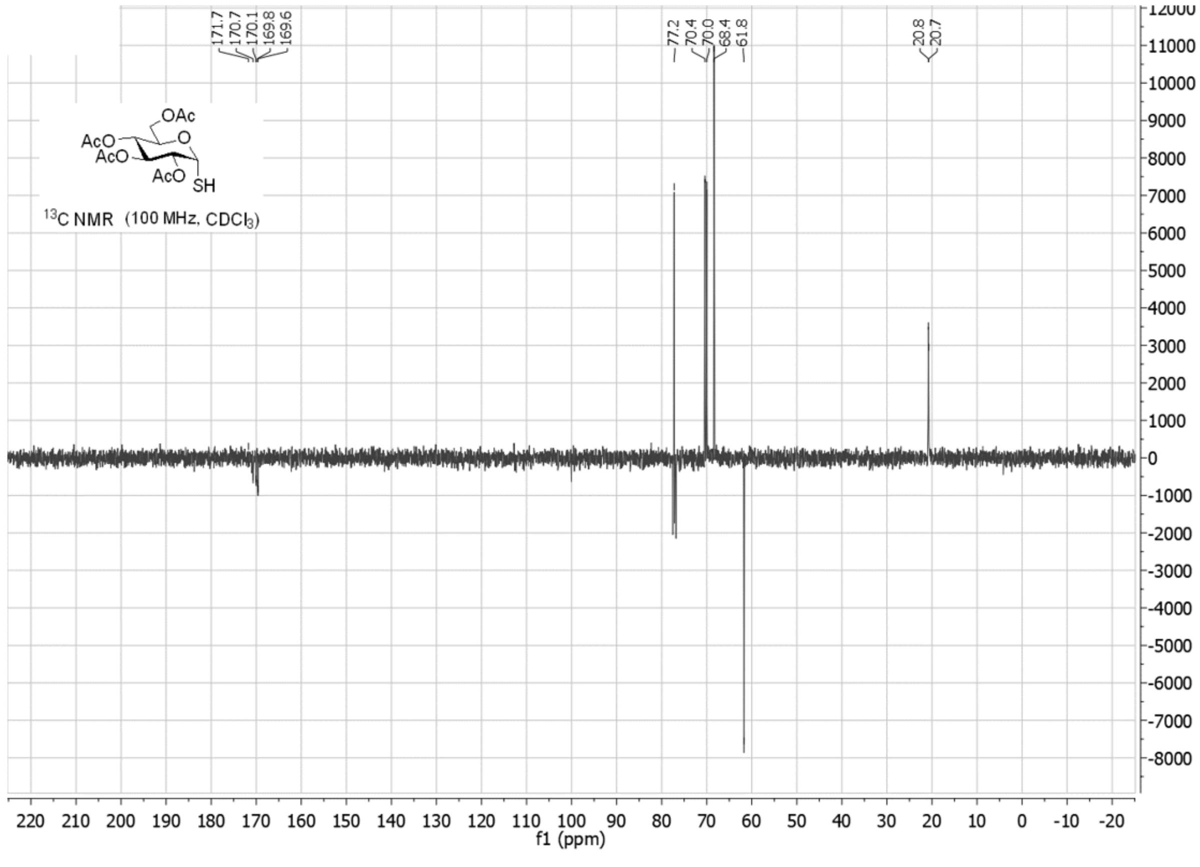

^1H and ^{13}C NMR spectra of compound **18**

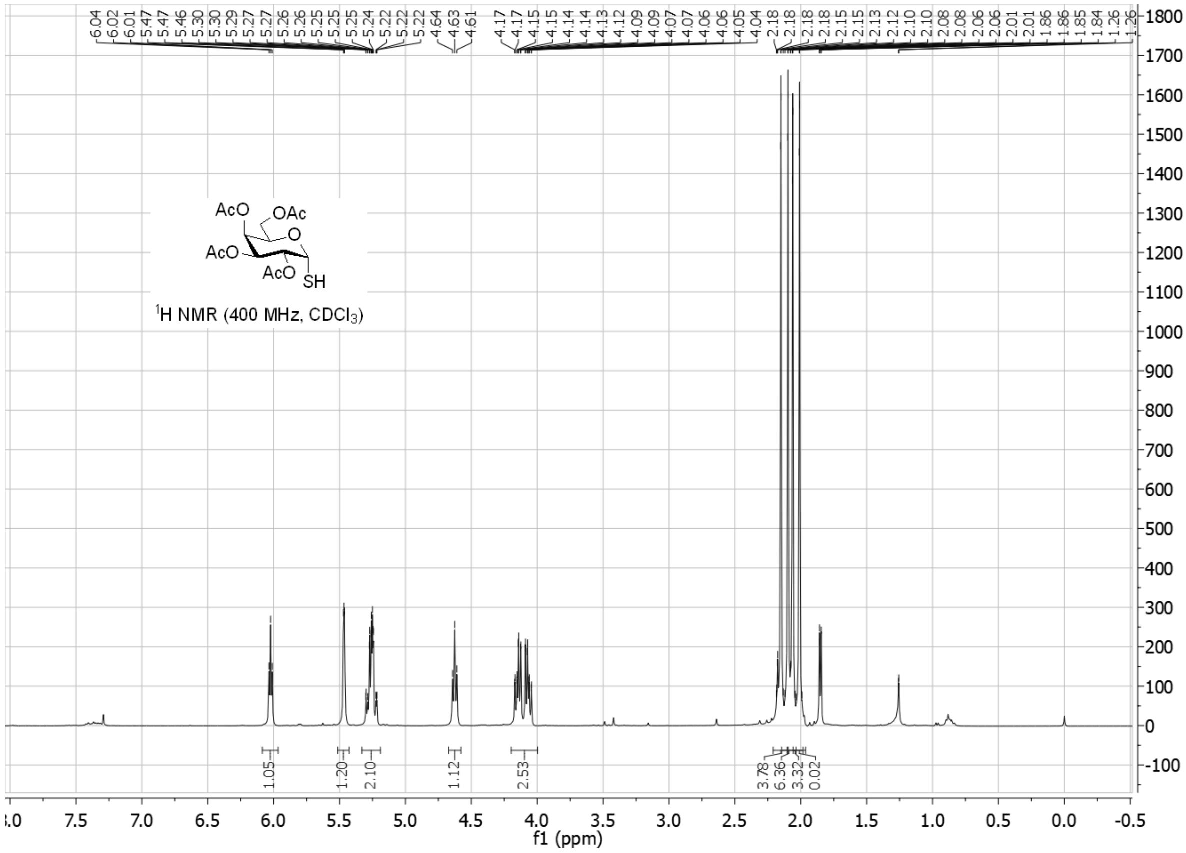

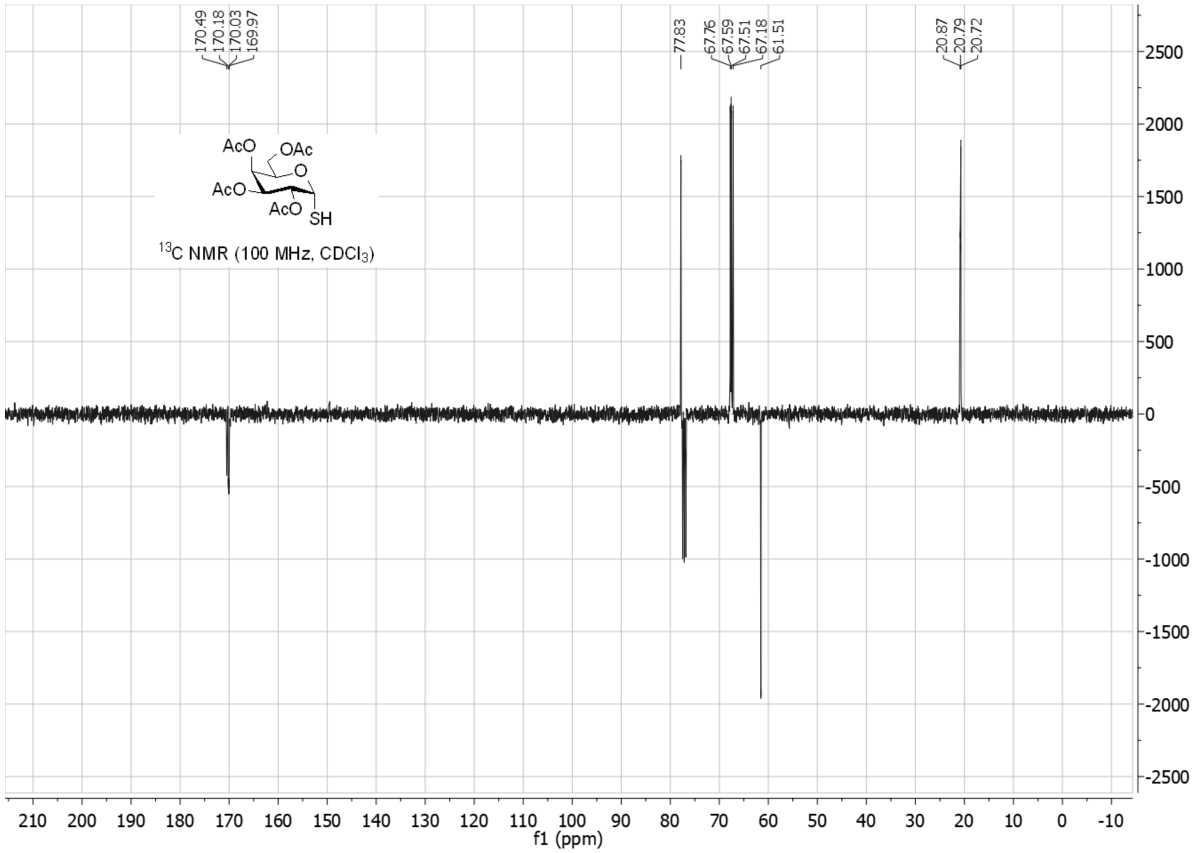

^{1}H and ^{13}C NMR spectra of compound **19**

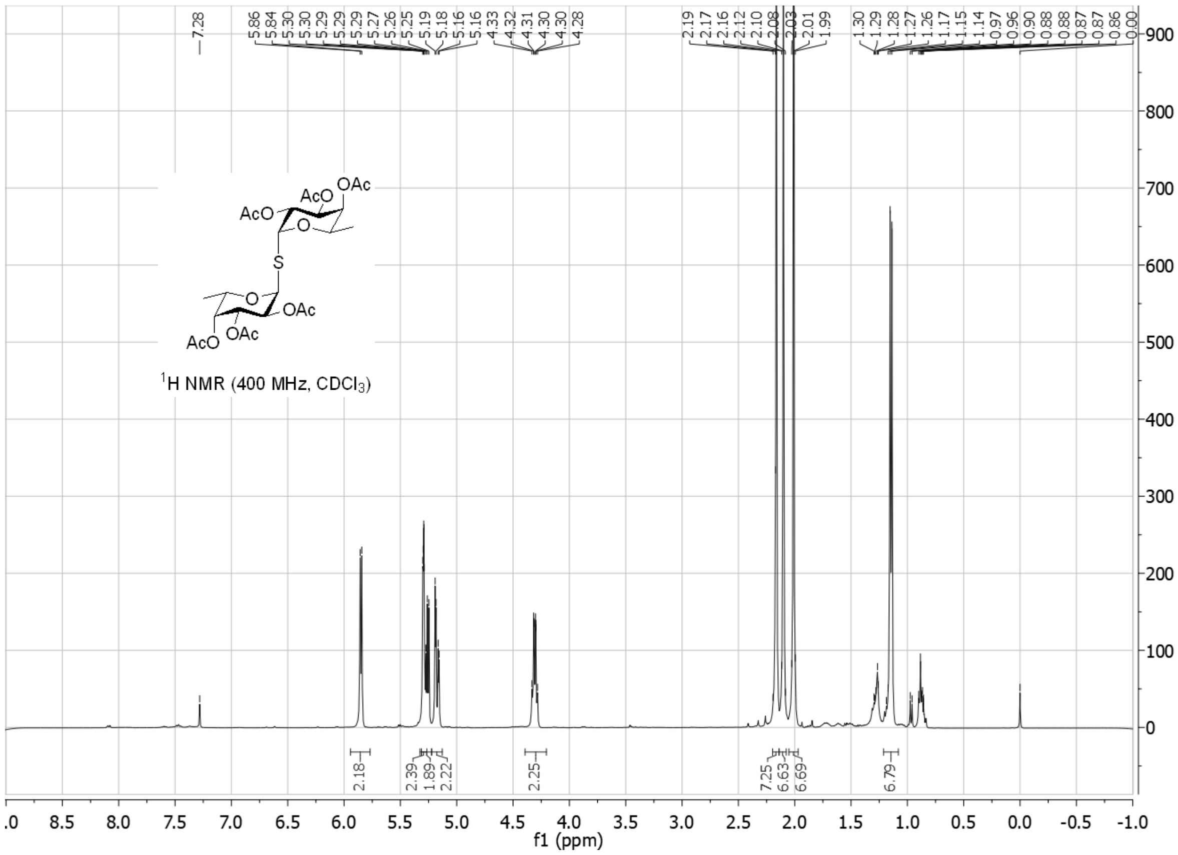

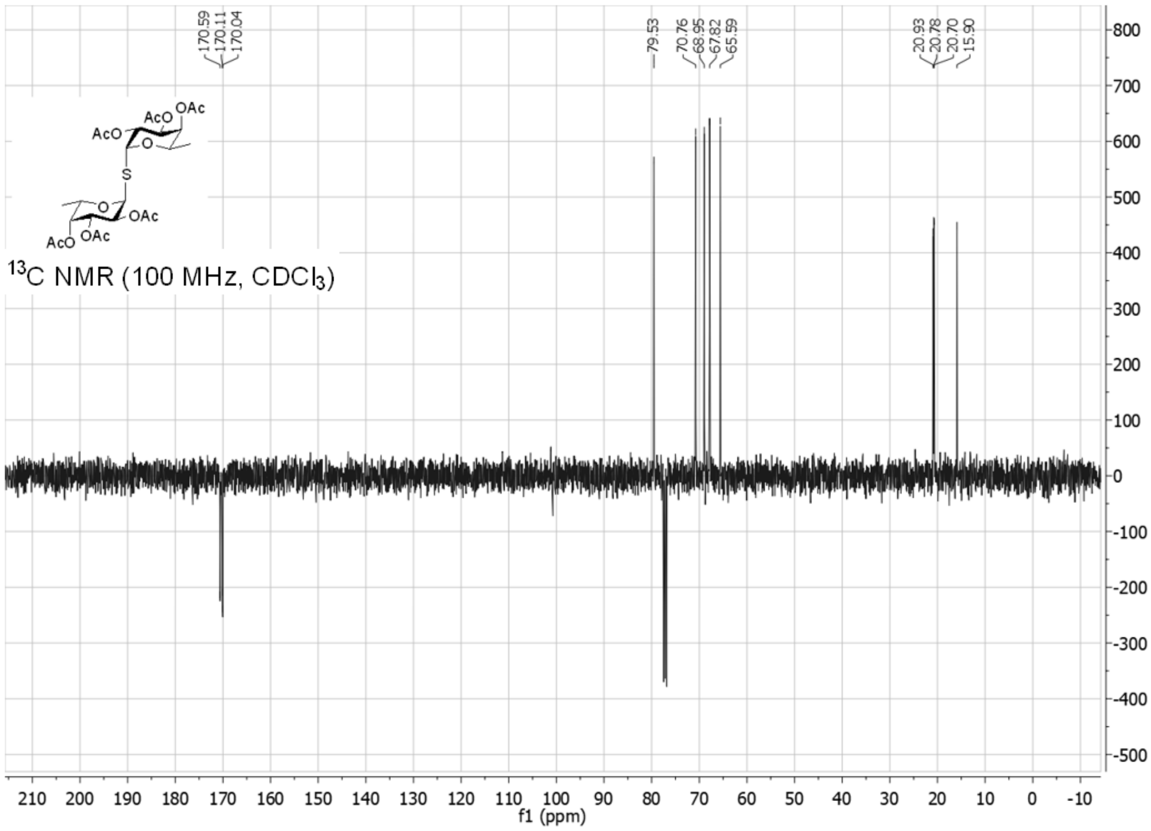

^{1}H and ^{13}C NMR spectra of compound **20**

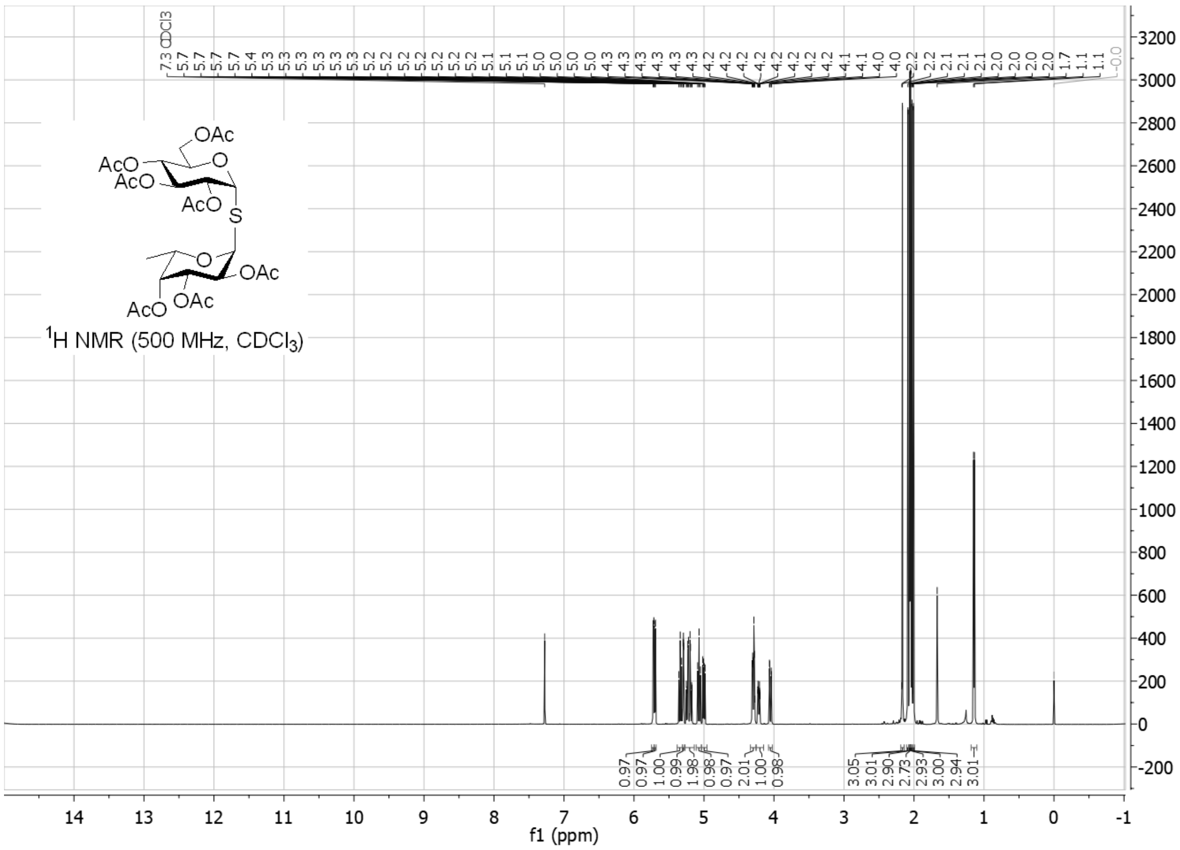

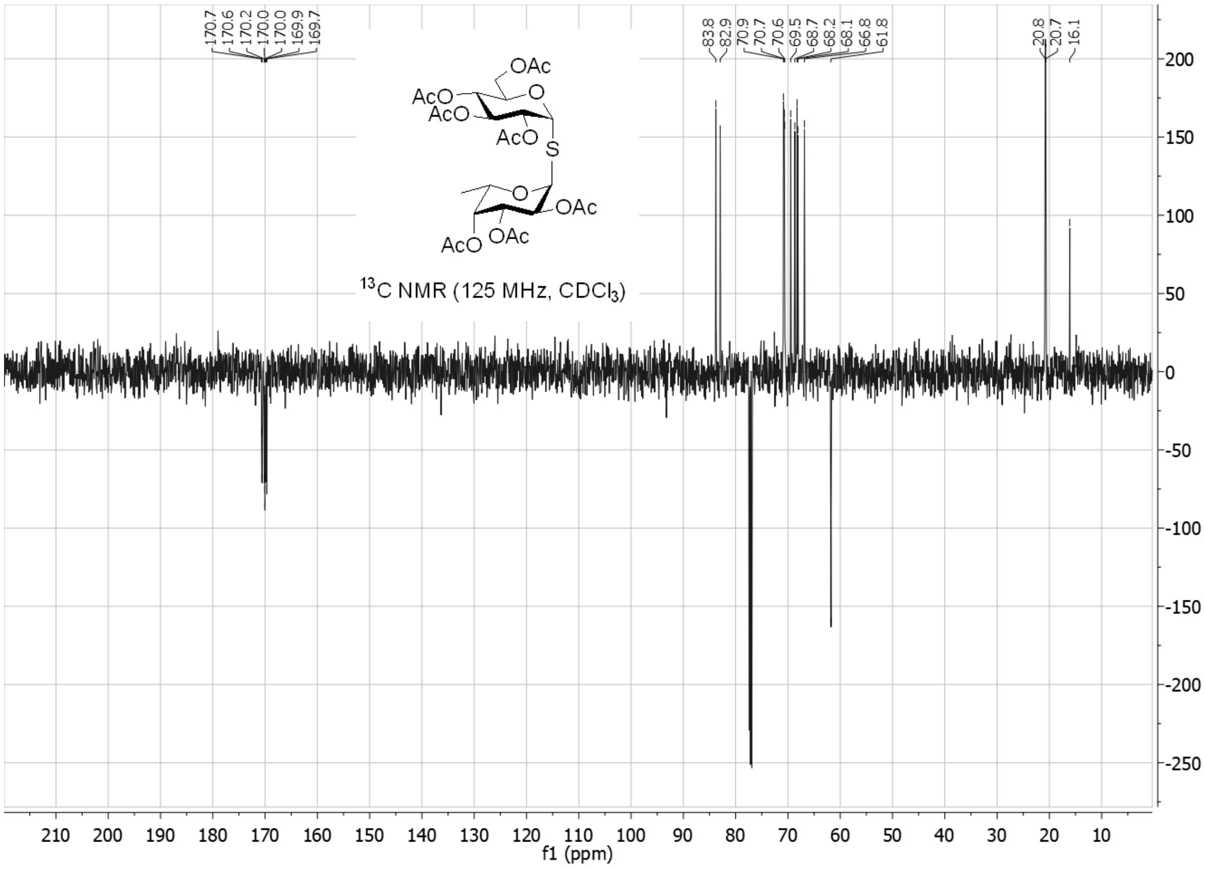

^{1}H and ^{13}C NMR spectra of compound **21**

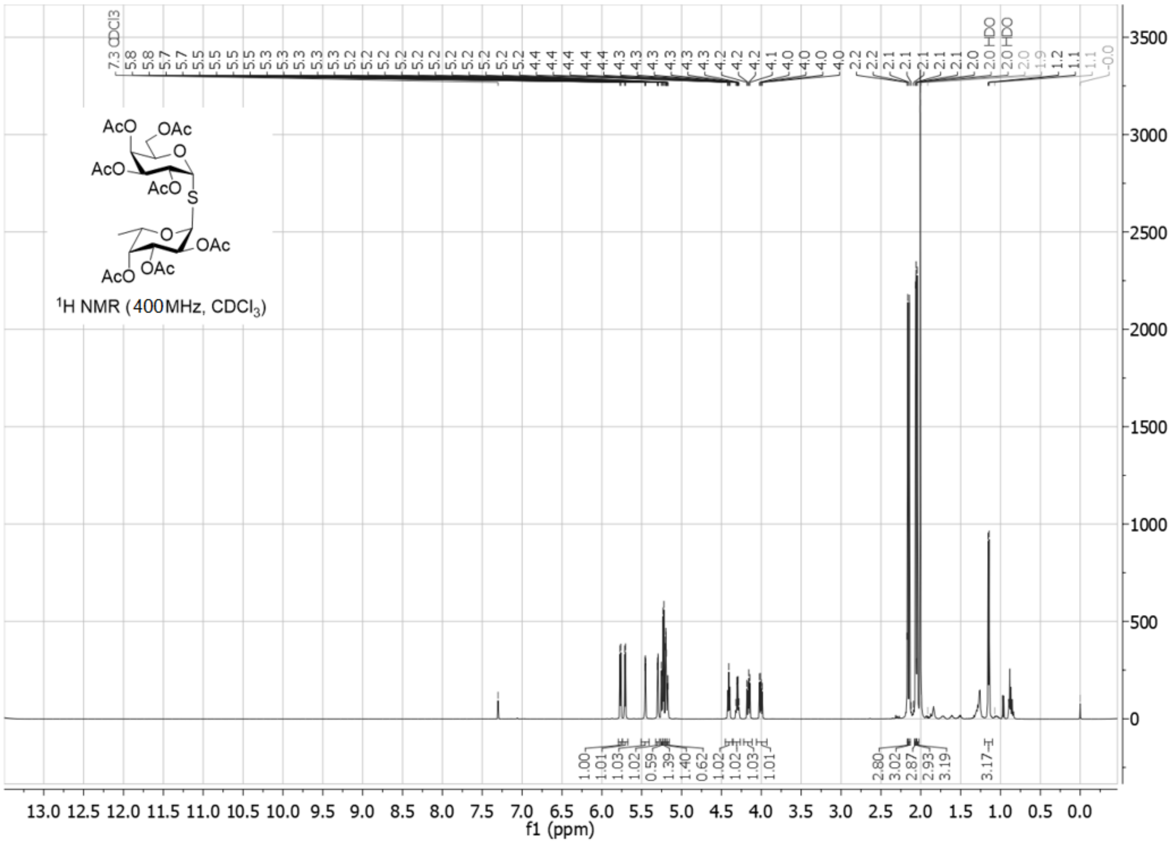

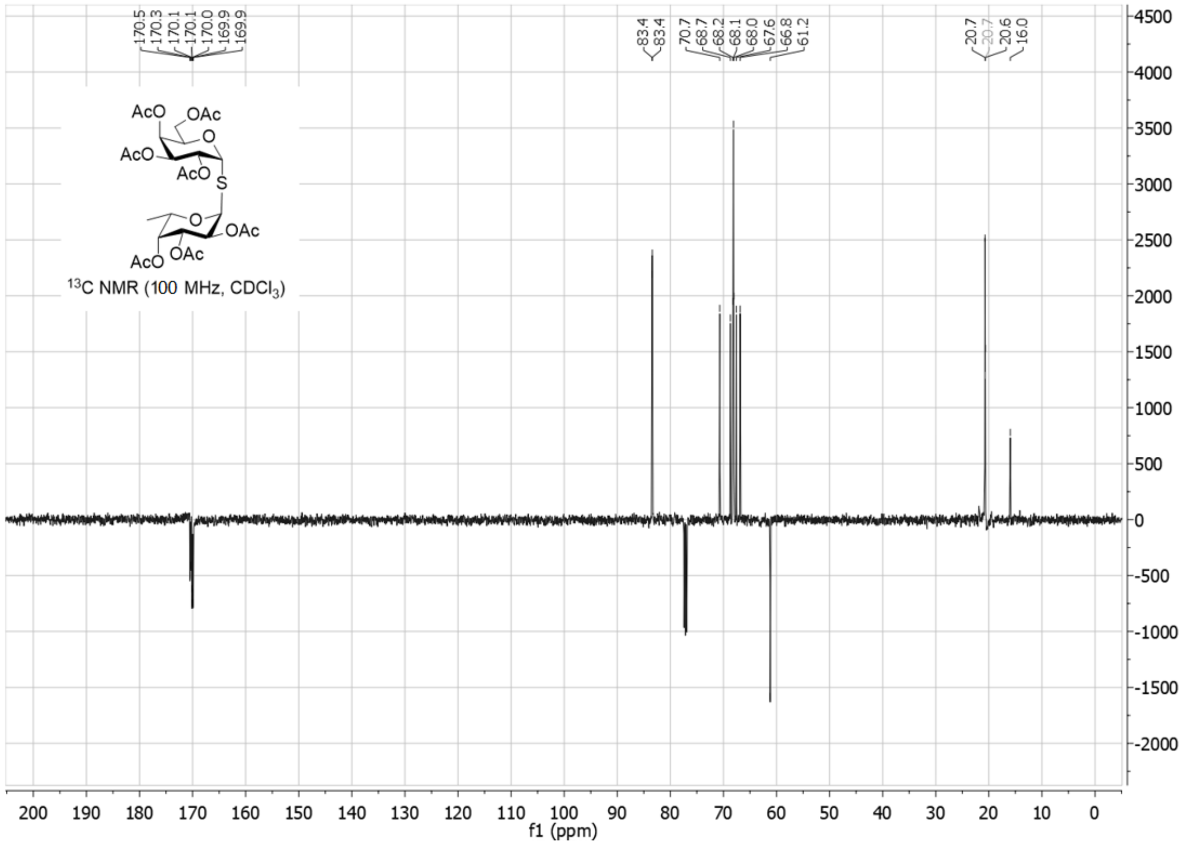

^{1}H and ^{13}C NMR spectra of compound **22**

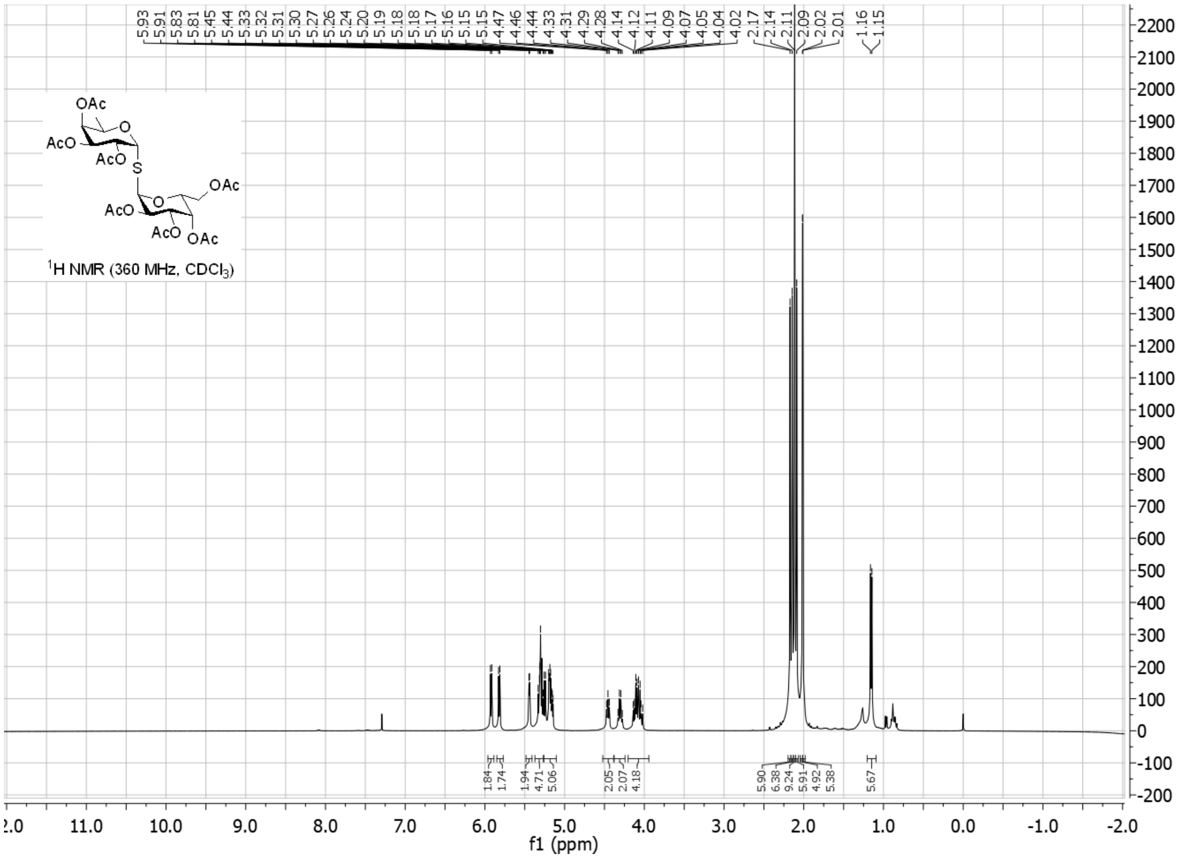

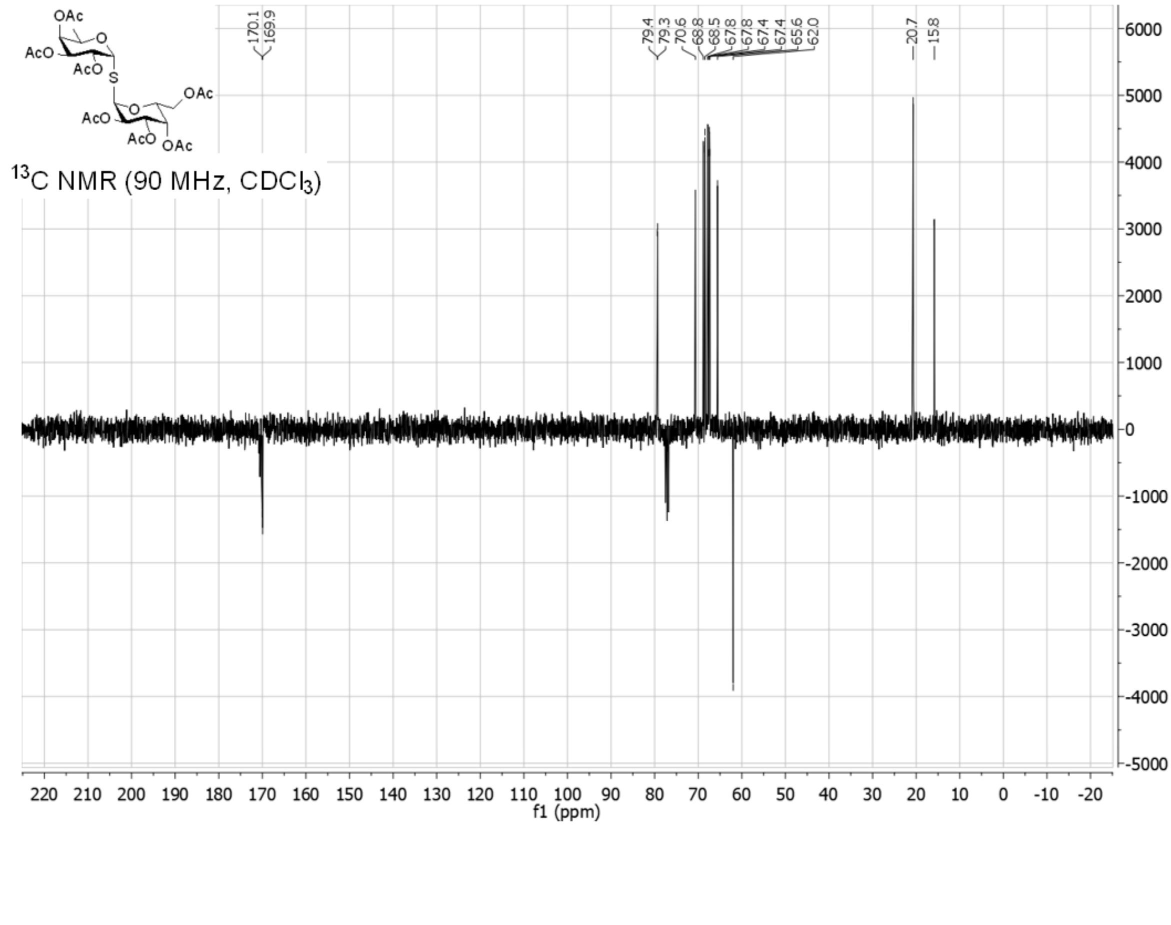

^{1}H and ^{13}C NMR spectra of compound **23**

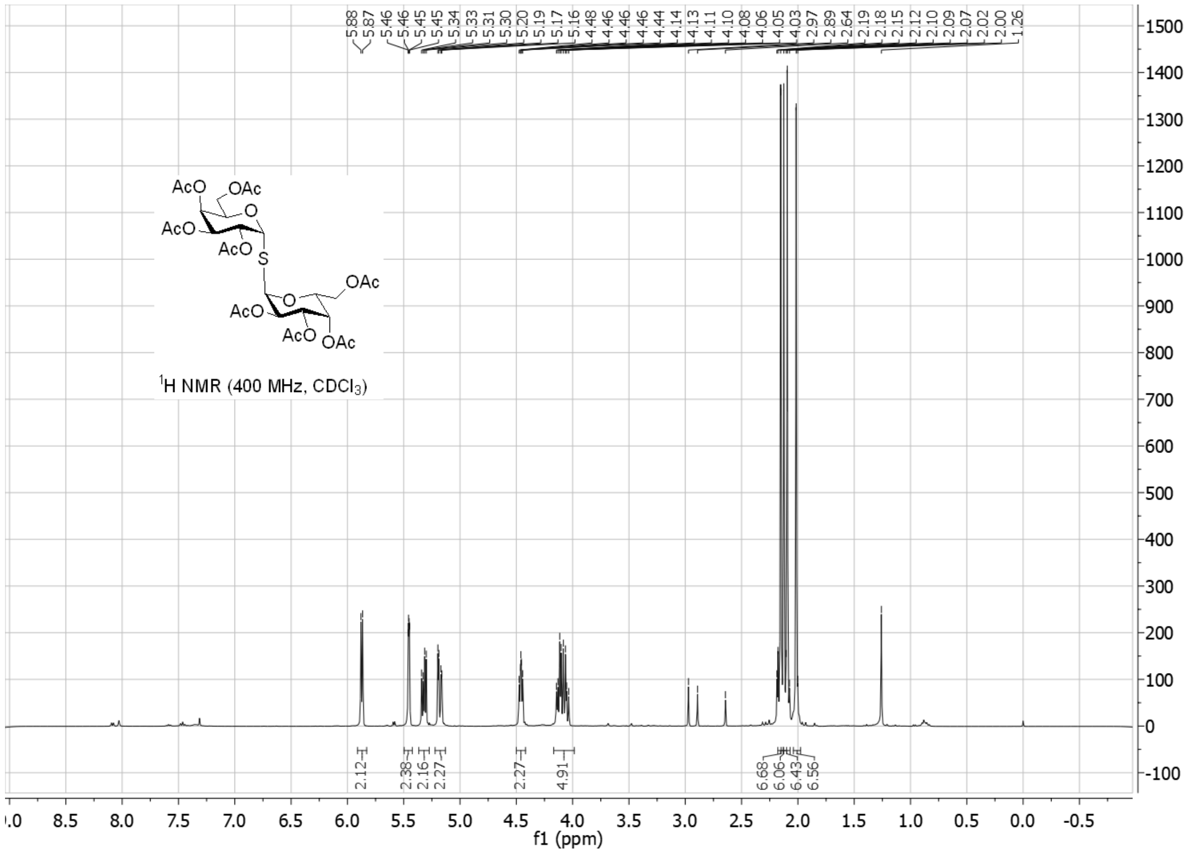

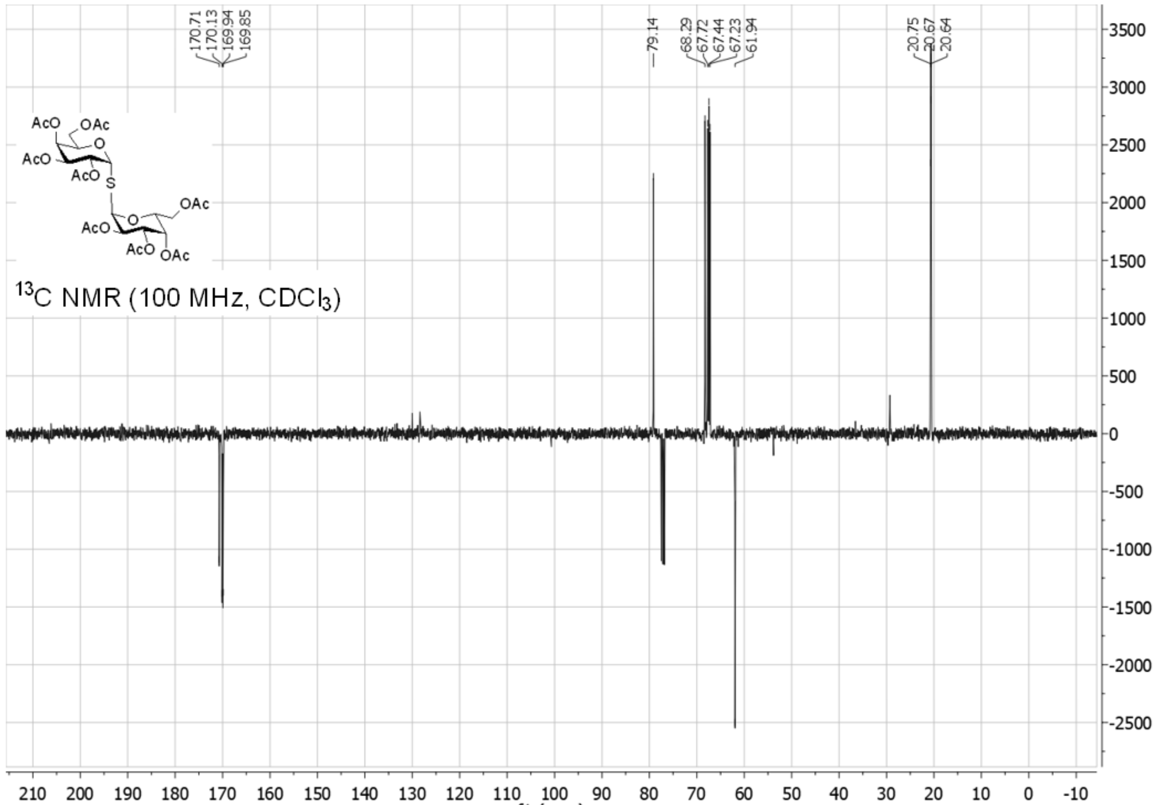

^{1}H and ^{13}C NMR spectra of compound **24**

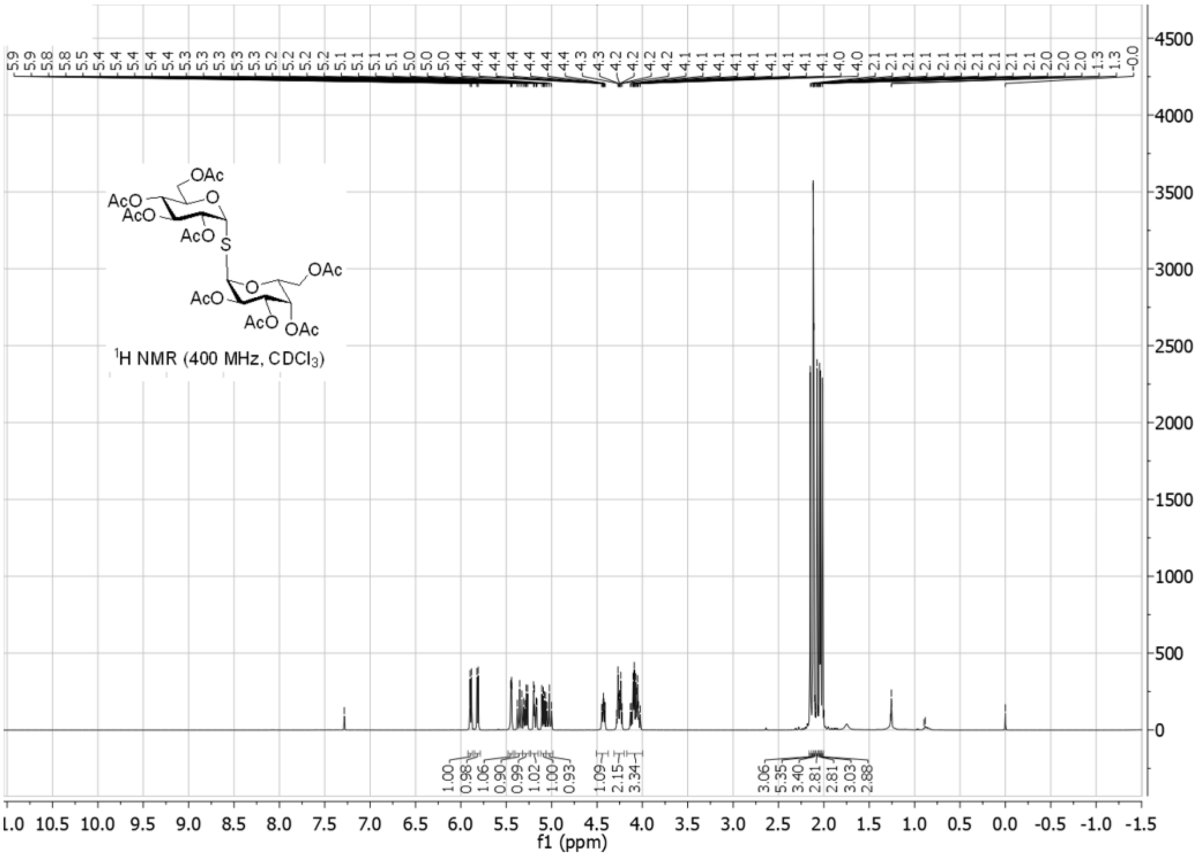

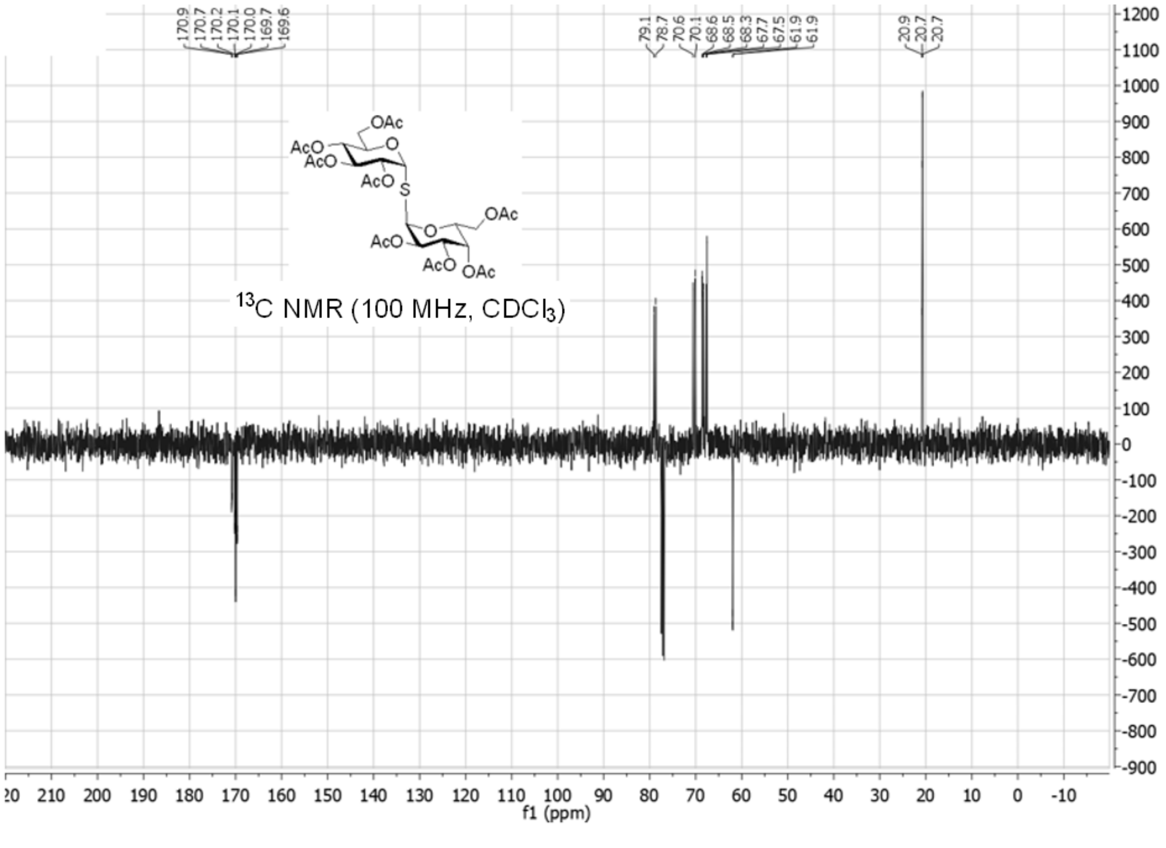

^{1}H and ^{13}C NMR spectra of compound **25**

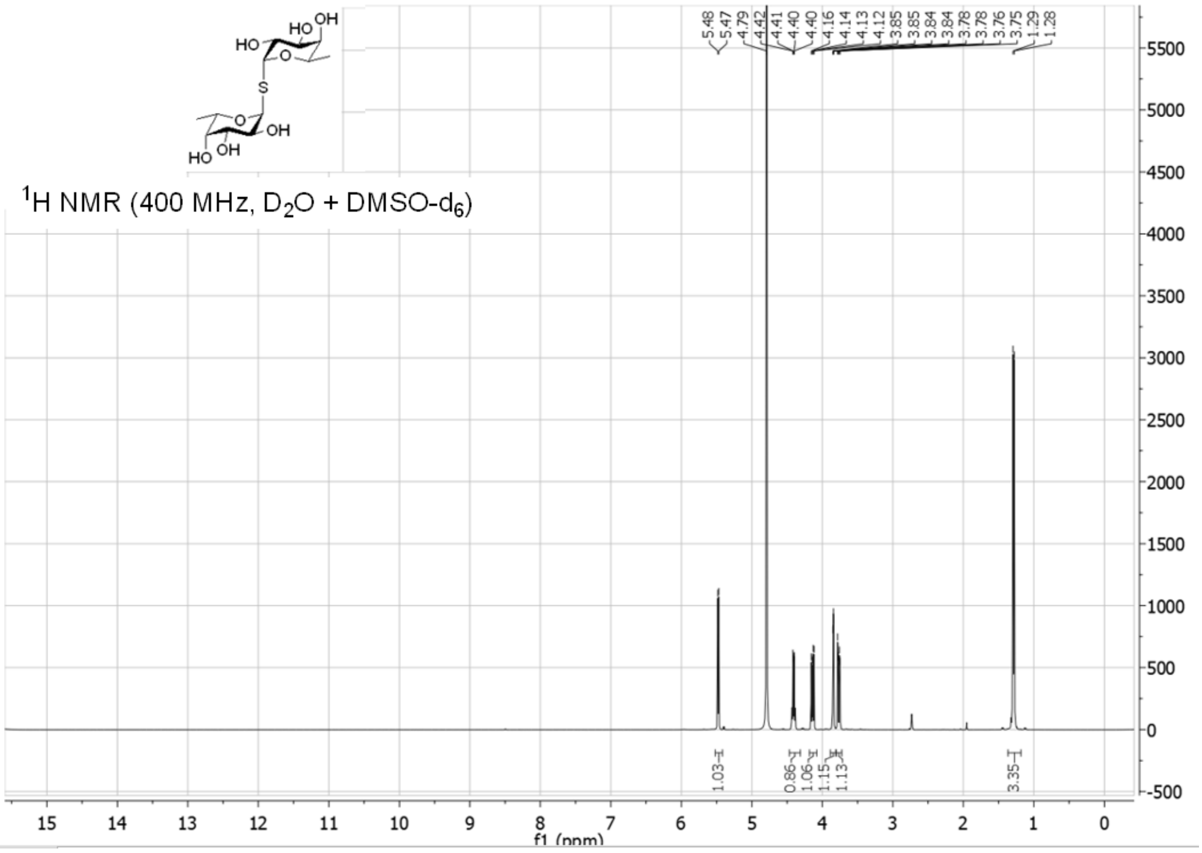

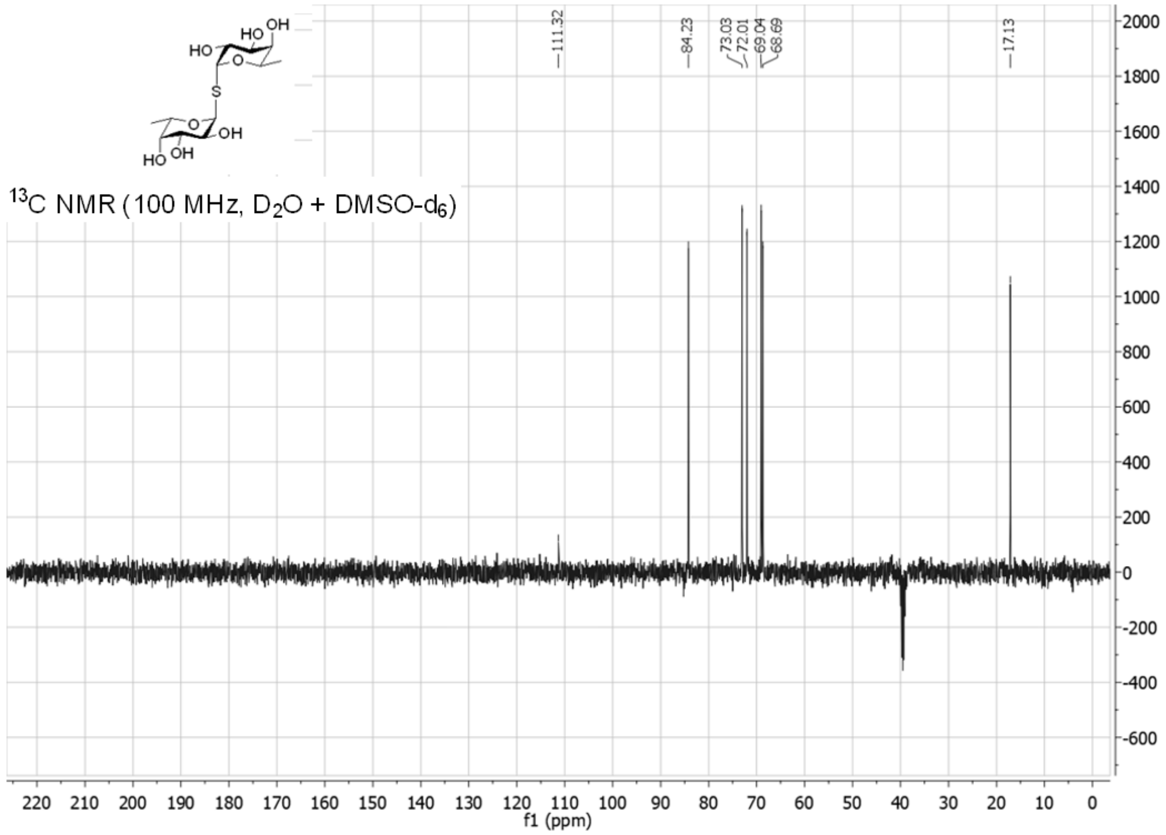

^{1}H and ^{13}C NMR spectra of compound **26**

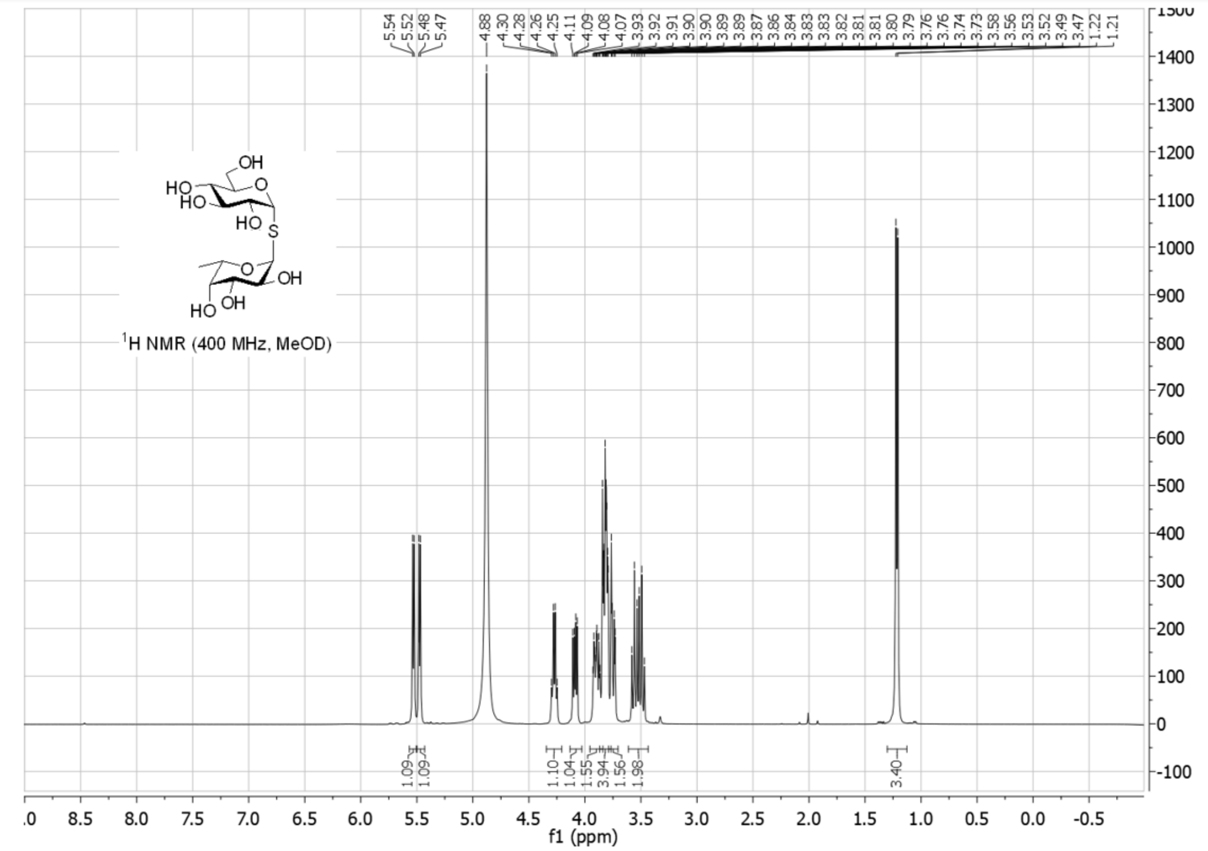

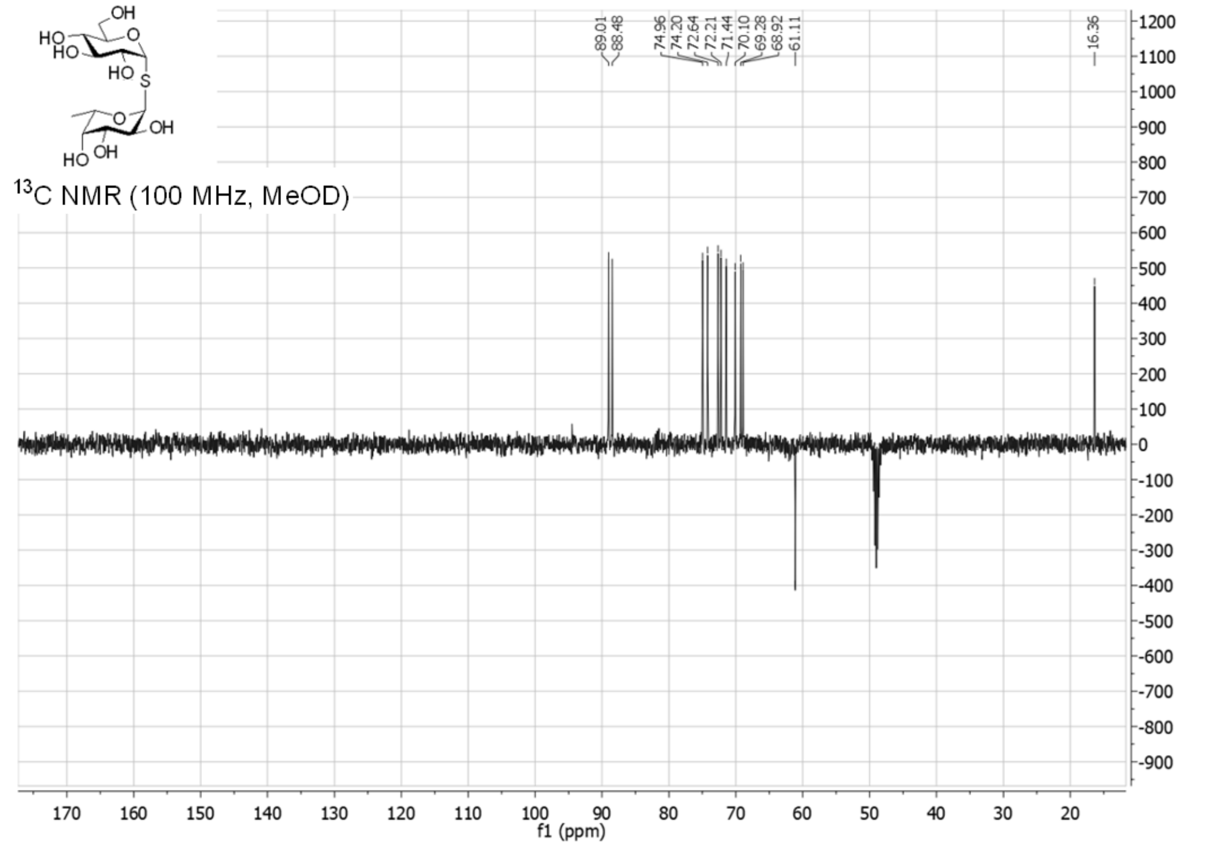

^{1}H and ^{13}C NMR spectra of compound **27**

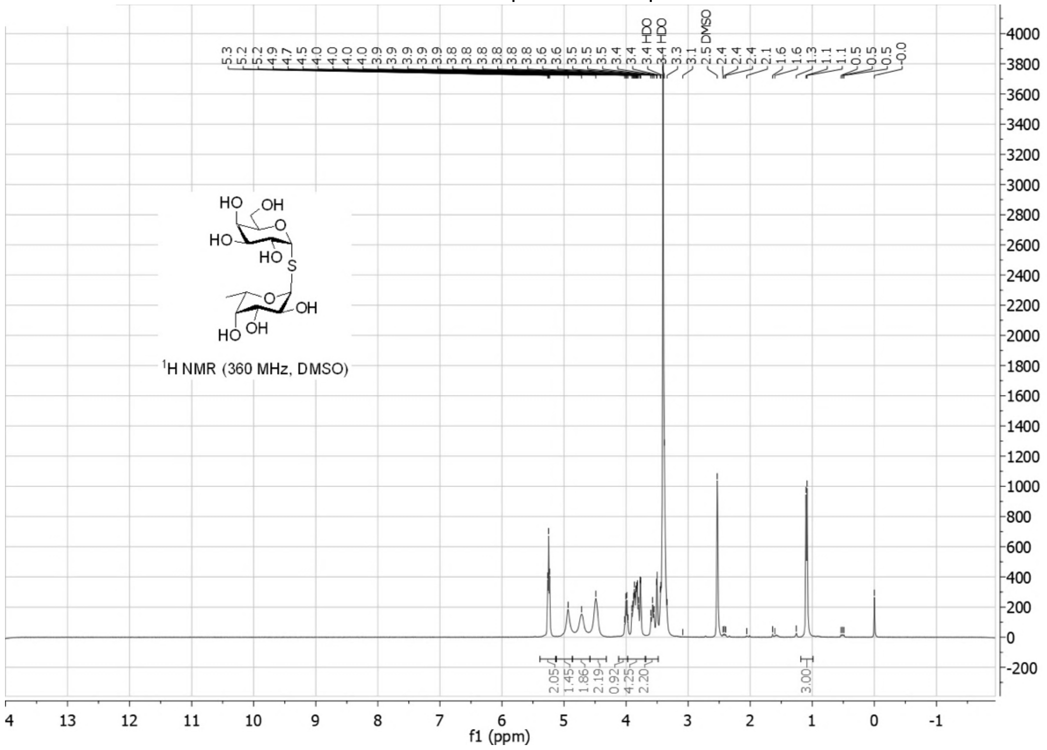

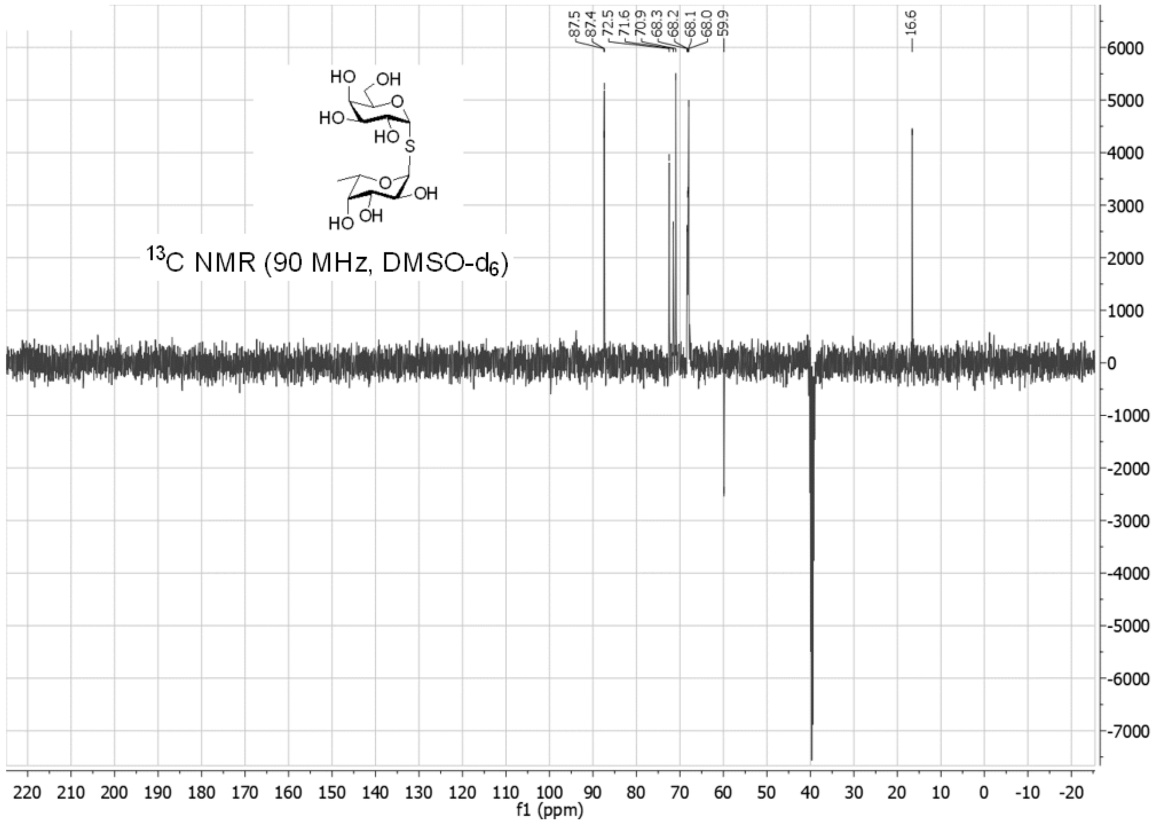

^{1}H and ^{13}C NMR spectra of compound **28**

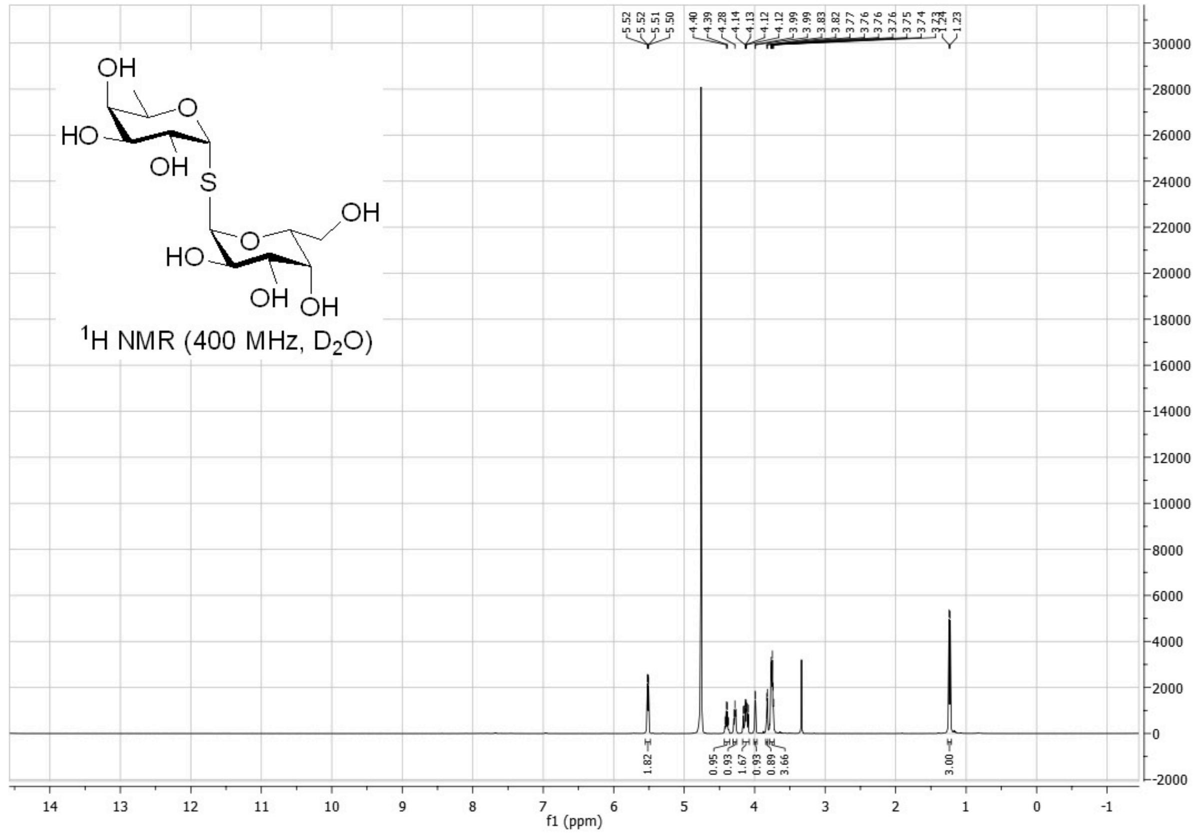

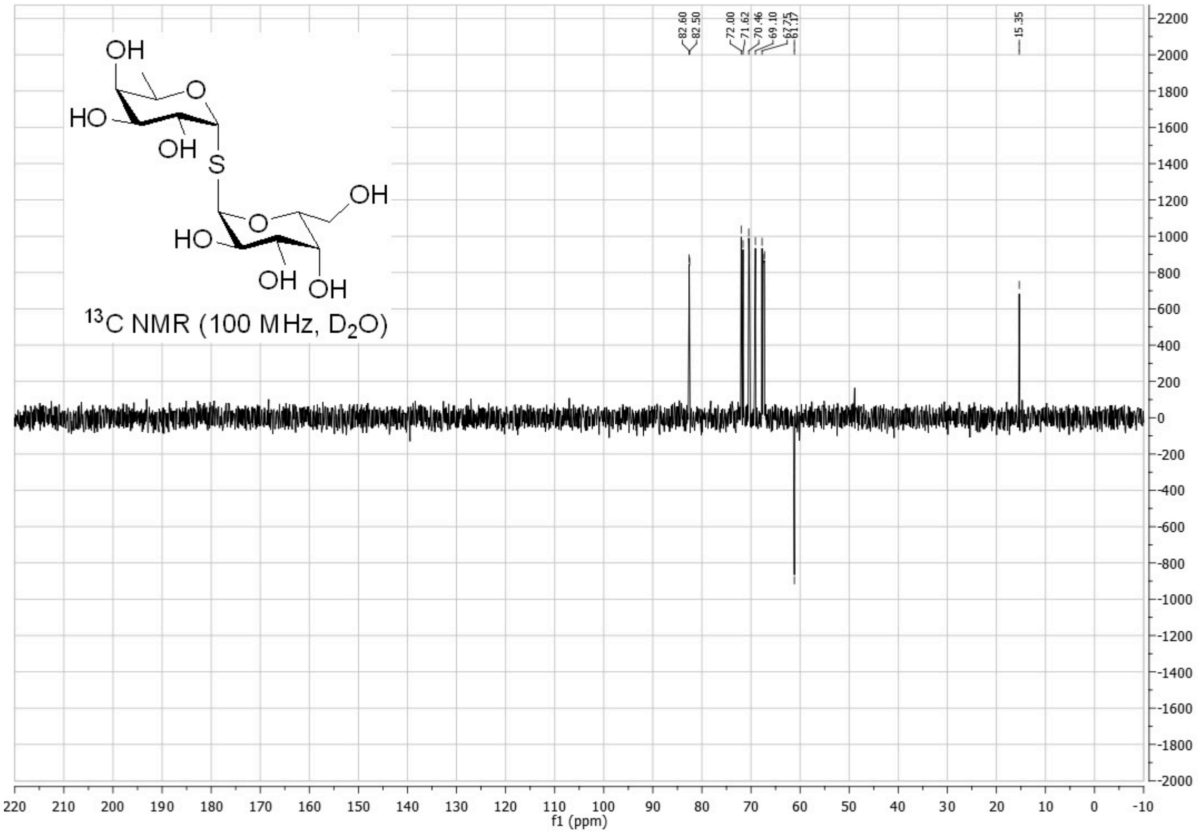

^{1}H and ^{13}C NMR spectra of compound **29**

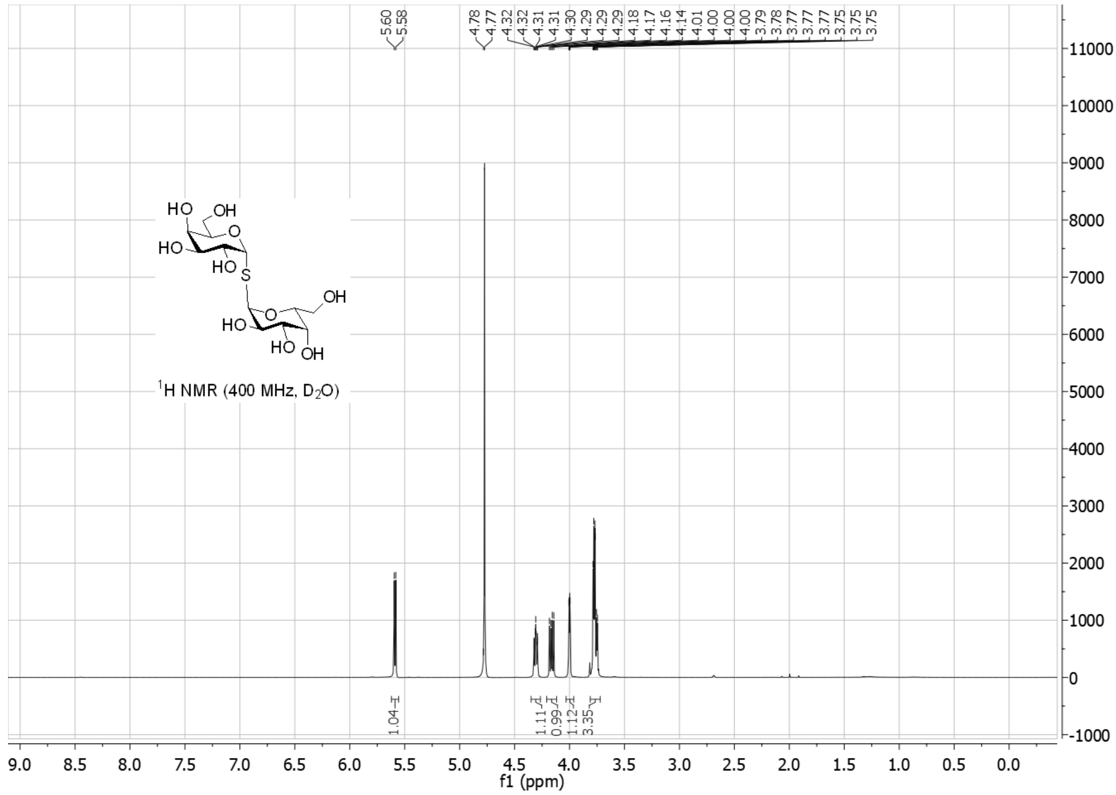

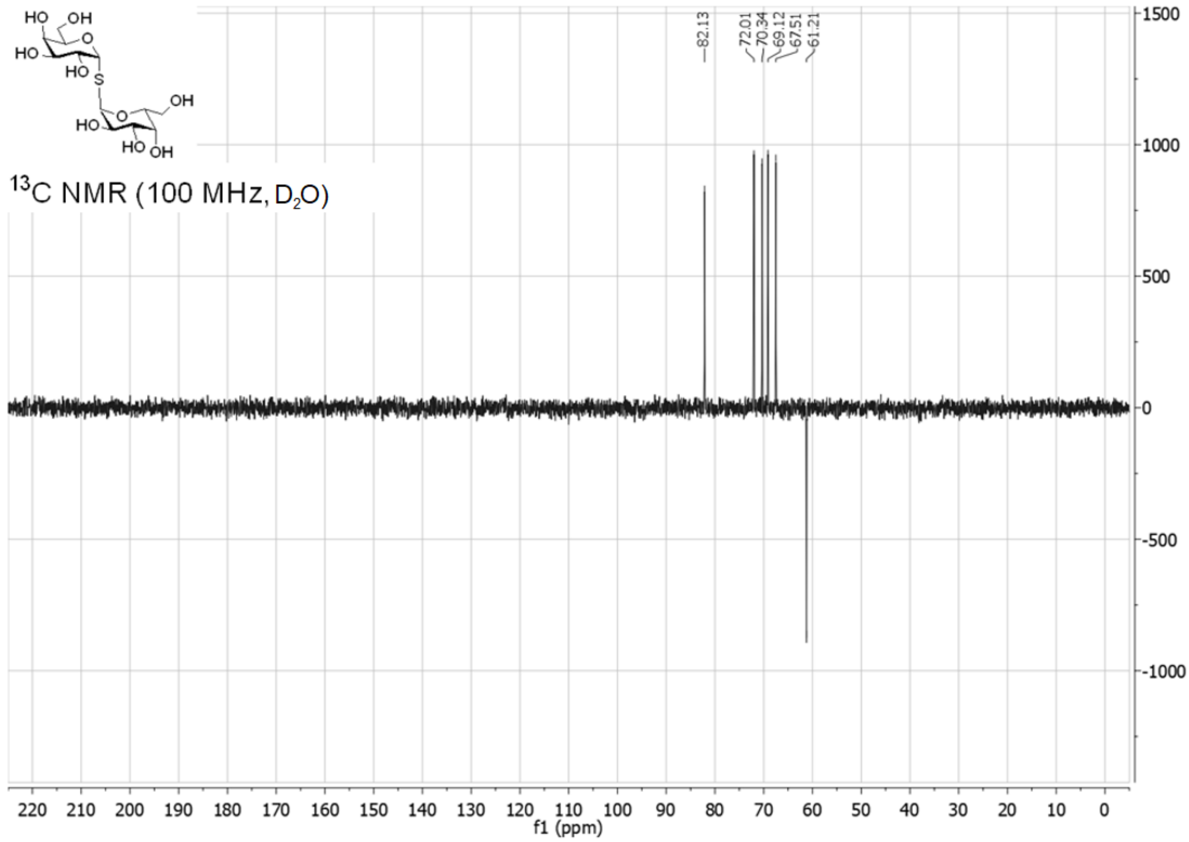

^{1}H and ^{13}C NMR spectra of compound **30**

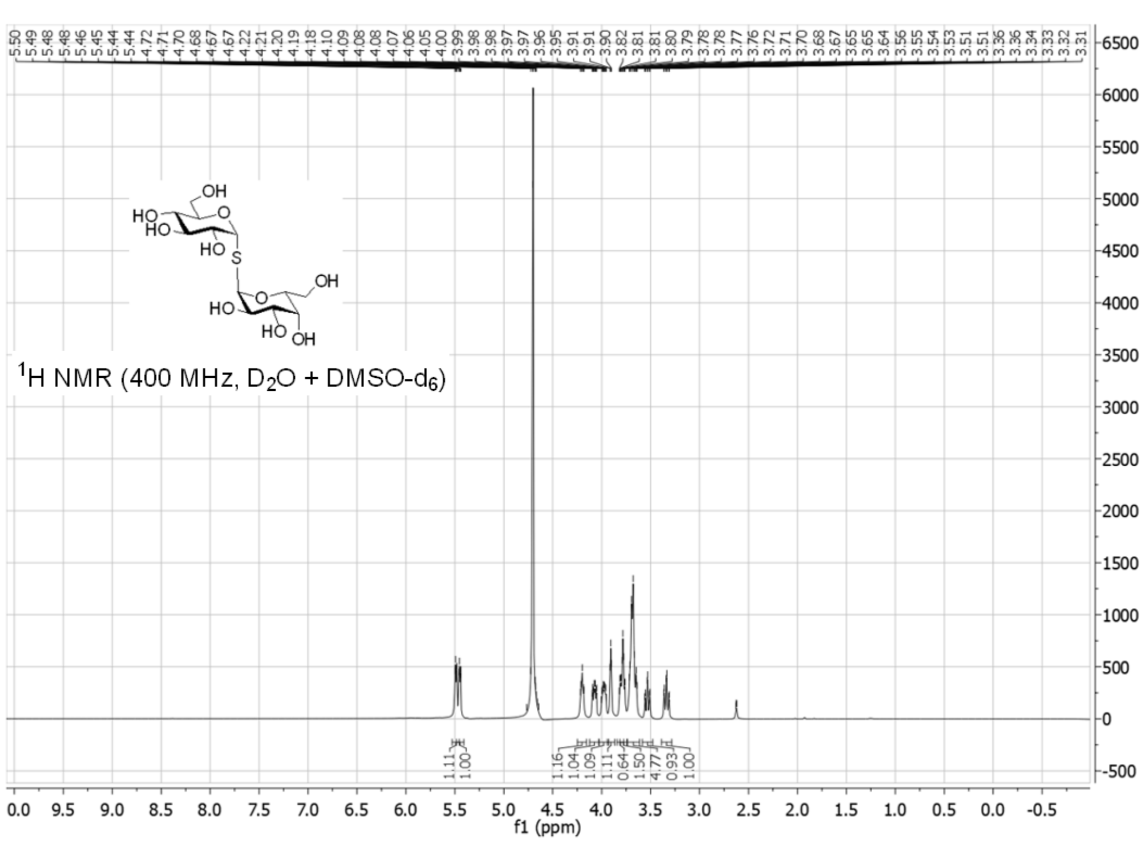

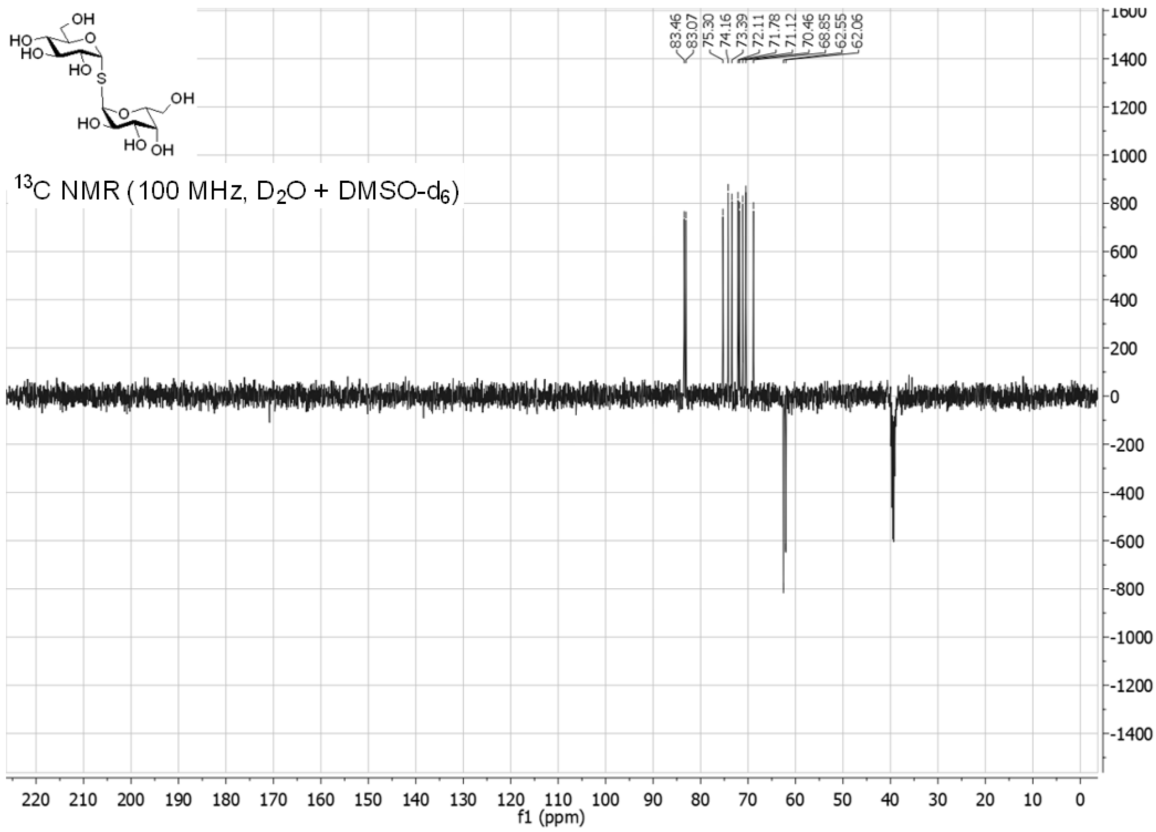

References

[1] C. Chemani, A. Imberty, S. De Bentzmann, M. Pierre, M. Wimmerová, B. P. Guery, K. Faure, *Infect Immun* **2009**, *77*, 2065–2075.

[2] A. M. Wu, J. H. Wu, T. Singh, J.-H. Liu, M.-S. Tsai, N. Gilboa-Garber, *Biochimie* **2006**, *88*, 1479–1492.

[3] G. J. L. Bernardes, D. P. Gamblin, B. G. Davis, *Angew Chem Int Ed* **2006**, *45*, 4007–4011.

[4] K. L. Matta., R. N. Girotra, J. F. Barlow, *Carbohydr. Res.* **1975**, *43*, 101–109.

[5] D. Horton, *Methods Carbohydr. Chem.* **1963**, *2*, 433–437.

[6] R. J. Ferrier, R. H. Furneaux, *Carbohydr. Res.* **1977**, *57*, 73–83.

[7] N. Floyd, B. Vijayakrishnan, J. R. Koeppe, B. G. Davis, *Angew Chem Int Ed* **2009**, *48*, 7798–7802.

[8] O. Varela, G. M. De Fina, R. M. De Lederkremer, *Carbohydr. Res.* **1987**, *167*, 187–196.

[9] D. R. Rao, L. M. Lerner, *Carbohydrate Research* **1972**, *22*, 345–350.

[10] V. Kelemen, M. Bege, N. Debreczeni, A. Borbás, *submitted* **n.d.**

[11] D. Eszenyi, V. Kelemen, F. Balogh, M. Bege, M. Csávás, P. Herczegh, A. Borbás, *Chemistry A European J* **2018**, *24*, 4532–4536.

[12] H. Hashimoto, K. Shimada, S. Horito, *Tetrahedron: Asymmetry* **1994**, *5*, 2351–2366.

[13] M. Sakata, M. Haga, S. Tejima, M. Akagi, *Chem. Pharm. Bull.* **1964**, *12*, 652–656.

[14] V. Kelemen, M. Bege, D. Eszenyi, N. Debreczeni, A. Bényei, T. Stürzer, P. Herczegh, A. Borbás, *Chemistry A European J* **2019**, *25*, 14555–14571.

[15] M. Blanc-Muesser, J. Defaye, H. Driguez, *Carbohydrate Research* **1978**, *67*, 305–328.

[16] L. M. Doyle, S. O'Sullivan, C. Di Salvo, M. McKinney, P. McArdle, P. V. Murphy, *Org. Lett.* **2017**, *19*, 5802–5805.

[17] L. Adamová, L. Malinovská, M. Wimmerová, *Microscopy Res & Technique* **2014**, *77*, 841–849.

[18] A. Novoa, T. Eierhoff, J. Topin, A. Varrot, S. Barluenga, A. Imberty, W. Römer, N. Winssinger, *Angew Chem Int Ed* **2014**, *53*, 8885–8889.

[19] P. Mała, E. Siebs, J. Meiers, K. Rox, A. Varrot, A. Imberty, A. Titz, *J. Med. Chem.* **2022**, *65*, 14180–14200.

[20] M. Cianci, G. Bourenkov, G. Pompidor, I. Karpics, J. Kallio, I. Bento, M. Roessle, F. Cipriani, S. Fiedler, T. R. Schneider, *J Synchrotron Rad* **2017**, *24*, 323–332.

[21] J. Gabadinho, A. Beteva, M. Guijarro, V. Rey-Bakaikoa, D. Spruce, M. W. Bowler, S. Brockhauser, D. Flot, E. J. Gordon, D. R. Hall, B. Lavault, A. A. McCarthy, J. McCarthy, E. Mitchell, S. Monaco, C. Mueller-Dieckmann, D. Nurizzo, R. B. G. Ravelli, X. Thibault, M. A. Walsh, G. A. Leonard, S. M. McSweeney, *J Synchrotron Rad* **2010**, *17*, 700–707.

[22] C. Vonrhein, C. Flensburg, P. Keller, A. Sharff, O. Smart, W. Paciorek, T. Womack, G. Bricogne, *Acta Crystallogr D Biol Crystallogr* **2011**, *67*, 293–302.

[23] P. Evans, *Acta Crystallogr D Biol Crystallogr* **2006**, *62*, 72–82.

[24] A. J. McCoy, R. W. Grosse-Kunstleve, P. D. Adams, M. D. Winn, L. C. Storoni, R. J. Read, *J Appl Crystallogr* **2007**, *40*, 658–674.

[25] G. N. Murshudov, P. Skubák, A. A. Lebedev, N. S. Pannu, R. A. Steiner, R. A. Nicholls, M. D. Winn, F. Long, A. A. Vagin, *Acta Crystallogr D Biol Crystallogr* **2011**, *67*, 355–367.

[26] P. Emsley, K. Cowtan, *Acta Crystallogr D Biol Crystallogr* **2004**, *60*, 2126–2132.

[27] A. A. Lebedev, P. Young, M. N. Isupov, O. V. Moroz, A. A. Vagin, G. N. Murshudov, *Acta Crystallogr D Biol Crystallogr* **2012**, *68*, 431–440.

[28] J. Agirre, M. Atanasova, H. Bagdonas, C. B. Ballard, A. Baslé, J. Beilsten-Edmands, R. J. Borges, D. G. Brown, J. J. Burgos-Mármol, J. M. Berrisford, P. S. Bond, I. Caballero, L. Catapano, G. Chojnowski, A. G. Cook, K. D. Cowtan, T. I. Croll, J. É. Debreczeni, N. E. Devenish, E. J. Dodson, T. R. Drevon, P. Emsley, G. Evans, P. R. Evans, M. Fando, J. Foadi, L. Fuentes-Montero, E. F. Garman, M. Gerstel, R. J. Gildea, K. Hatti, M. L. Hekkelman, P. Heuser, S. W. Hoh, M. A. Hough, H. T. Jenkins, E. Jiménez, R. P. Joosten, R. M. Keegan, N. Keep, E. B. Krissinel, P. Kolenko, O. Kovalevskiy, V. S. Lamzin, D. M. Lawson, A. A. Lebedev, A. G. W. Leslie, B. Lohkamp, F. Long, M. Malý, A. J. McCoy, S. J. McNicholas, A. Medina, C. Millán, J. W. Murray, G. N. Murshudov, R. A. Nicholls, M. E. M.

Noble, R. Oeffner, N. S. Pannu, J. M. Parkhurst, N. Pearce, J. Pereira, A. Perrakis, H. R. Powell, R. J. Read, D. J. Rigden, W. Rochira, M. Sammito, F. Sánchez Rodríguez, G. M. Sheldrick, K. L. Shelley, F. Simkovic, A. J. Simpkin, P. Skubak, E. Sobolev, R. A. Steiner, K. Stevenson, I. Tews, J. M. H. Thomas, A. Thorn, J. T. Valls, V. Uski, I. Usón, A. Vagin, S. Velankar, M. Vollmar, H. Walden, D. Waterman, K. S. Wilson, M. D. Winn, G. Winter, M. Wojdyr, K. Yamashita, *Acta Crystallogr D Struct Biol* **2023**, *79*, 449–461.

[29] C. A. Brautigam, *Meth. Enzymol.* **2015**, *562*, 109–133.
